# A first update on mapping the human genetic architecture of COVID-19

**DOI:** 10.1038/s41586-022-04826-7

**Published:** 2022-08-03

**Authors:** COVID-19 Host Genetics Initiative, Gita A. Pathak, Juha Karjalainen, Christine Stevens, Benjamin M. Neale, Mark Daly, Andrea Ganna, Gita A. Pathak, Andrea Ganna, Shea J. Andrews, Masahiro Kanai, Mattia Cordioli, Juha Karjalainen, Gita A. Pathak, Renato Polimanti, Shea J. Andrews, Nadia Harerimana, Mattia Cordioli, Matti Pirinen, Masahiro Kanai, Christine Stevens, Rachel G. Liao, Karolina Chwialkowska, Amy Trankiem, Mary K. Balaconis, Huy Nguyen, Matthew Solomonson, Kumar Veerapen, Brooke Wolford, Genevieve Roberts, Danny Park, Catherine A. Ball, Marie Coignet, Shannon McCurdy, Spencer Knight, Raghavendran Partha, Brooke Rhead, Miao Zhang, Nathan Berkowitz, Michael Gaddis, Keith Noto, Luong Ruiz, Milos Pavlovic, Eurie L. Hong, Kristin Rand, Ahna Girshick, Harendra Guturu, Asher Haug Baltzell, Mari E. K. Niemi, Souad Rahmouni, Julien Guntz, Yves Beguin, Mattia Cordioli, Sara Pigazzini, Lindokuhle Nkambule, Michel Georges, Michel Moutschen, Benoit Misset, Gilles Darcis, Julien Guiot, Samira Azarzar, Stéphanie Gofflot, Sabine Claassen, Olivier Malaise, Pascale Huynen, Christelle Meuris, Marie Thys, Jessica Jacques, Philippe Léonard, Frederic Frippiat, Jean-Baptiste Giot, Anne-Sophie Sauvage, Christian Von Frenckell, Yasmine Belhaj, Bernard Lambermont, Tomoko Nakanishi, David R. Morrison, Vincent Mooser, J. Brent Richards, Guillaume Butler-Laporte, Vincenzo Forgetta, Rui Li, Biswarup Ghosh, Laetitia Laurent, Alexandre Belisle, Danielle Henry, Tala Abdullah, Olumide Adeleye, Noor Mamlouk, Nofar Kimchi, Zaman Afrasiabi, Nardin Rezk, Branka Vulesevic, Meriem Bouab, Charlotte Guzman, Louis Petitjean, Chris Tselios, Xiaoqing Xue, Jonathan Afilalo, Marc Afilalo, Maureen Oliveira, Bluma Brenner, Nathalie Brassard, Madeleine Durand, Erwin Schurr, Pierre Lepage, Jiannis Ragoussis, Daniel Auld, Michaël Chassé, Daniel E. Kaufmann, G. Mark Lathrop, Darin Adra, Caroline Hayward, Joseph T. Glessner, Douglas M. Shaw, Archie Campbell, Marcela Morris, Hakon Hakonarson, David J. Porteous, Jennifer Below, Anne Richmond, Xiao Chang, Hannah Polikowski, Petty E. Lauren, Hung-Hsin Chen, Zhu Wanying, Chloe Fawns-Ritchie, Kari North, Joseph B. McCormick, Xiao Chang, Joseph R. Glessner, Hakon Hakonarson, Christopher R. Gignoux, Stephen J. Wicks, Kristy Crooks, Kathleen C. Barnes, Michelle Daya, Jonathan Shortt, Nicholas Rafaels, Sameer Chavan, Paul R. H. J. Timmers, James F. Wilson, Albert Tenesa, Shona M. Kerr, Kenton D’Mellow, Mari E. K. Niemi, Doaa Shahin, Yasser M. El-Sherbiny, Kathrin Aprile von Hohenstaufen, Ali Sobh, Madonna M. Eltoukhy, Mattia Cordioli, Lindokuhle Nkambul, Tamer A. Elhadidy, Mohamed S. Abd Elghafar, Jehan J. El-Jawhari, Attia A. S. Mohamed, Marwa H. Elnagdy, Amr Samir, Mahmoud Abdel-Aziz, Walid T. Khafaga, Walaa M. El-Lawaty, Mohamed S. Torky, Mohamed R. El-shanshory, Amr M. Yassen, Mohamed A. F. Hegazy, Kamal Okasha, Mohammed A. Eid, Hanteera S. Moahmed, Carolina Medina-Gomez, M. Arfan Ikram, Andre G. Uitterlinden, Reedik Mägi, Lili Milani, Andres Metspalu, Triin Laisk, Kristi Läll, Maarja Lepamets, Tõnu Esko, Ene Reimann, Paul Naaber, Edward Laane, Jaana Pesukova, Pärt Peterson, Kai Kisand, Jekaterina Tabri, Raili Allos, Kati Hensen, Joel Starkopf, Inge Ringmets, Anu Tamm, Anne Kallaste, Helene Alavere, Kristjan Metsalu, Mairo Puusepp, Chiara Batini, Martin D. Tobin, Laura D. Venn, Paul H. Lee, Nick Shrine, Alexander T. Williams, Anna L. Guyatt, Catherine John, Richard J. Packer, Altaf Ali, Robert C. Free, Xueyang Wang, Louise V. Wain, Edward J. Hollox, Catherine E. Bee, Emma L. Adams, Aarno Palotie, Samuli Ripatti, Sanni Ruotsalainen, Kati Kristiansson, Sami Koskelainen, Markus Perola, Kati Donner, Katja Kivinen, Aarno Palotie, Mari Kaunisto, Carlo Rivolta, Pierre-Yves Bochud, Stéphanie Bibert, Noémie Boillat, Semira Gonseth Nussle, Werner Albrich, Mathieu Quinodoz, Dhryata Kamdar, Noémie Suh, Dionysios Neofytos, Véronique Erard, Cathy Voide, Pierre-Yves Bochud, Carlo Rivolta, Stéphanie Bibert, Mathieu Quinodoz, Dhryata Kamdar, Dionysios Neofytos, Véronique Erard, Cathy Voide, R. Friolet, P. Vollenweider, J. L. Pagani, M. Oddo, F. Meyer zu Bentrup, A. Conen, O. Clerc, O. Marchetti, A. Guillet, C. Guyat-Jacques, S. Foucras, M. Rime, J. Chassot, M. Jaquet, R. Merlet Viollet, Y. Lannepoudenx, L. Portopena, P. Y. Bochud, P. Vollenweider, J. L. Pagani, F. Desgranges, P. Filippidis, B. Guéry, D. Haefliger, E. E. Kampouri, O. Manuel, A. Munting, M. Papadimitriou-Olivgeris, J. Regina, L. Rochat-Stettler, V. Suttels, E. Tadini, J. Tschopp, M. Van Singer, B. Viala, N. Boillat-Blanco, T. Brahier, O. Hügli, J. Y. Meuwly, O. Pantet, S. Gonseth Nussle, M. Bochud, V. D’Acremont, S. Estoppey Younes, W. C. Albrich, N. Suh, A. Cerny, L. O’Mahony, C. von Mering, P. Y. Bochud, M. Frischknecht, G-R. Kleger, M. Filipovic, C. R. Kahlert, H. Wozniak, T. Rochat Negro, J. Pugin, K. Bouras, C. Knapp, T. Egger, A. Perret, P. Montillier, C. di Bartolomeo, B. Barda, Rafael de Cid, Anna Carreras, Victor Moreno, Manolis Kogevinas, Iván Galván-Femenía, Natalia Blay, Xavier Farré, Lauro Sumoy, Beatriz Cortés, Josep Maria Mercader, Marta Guindo-Martinez, David Torrents, Judith Garcia-Aymerich, Gemma Castaño-Vinyals, Carlota Dobaño, Mari E. K. Niemi, Marco Gori, Alessandra Renieri, Francesca Mari, Mario Umberto Mondelli, Francesco Castelli, Massimo Vaghi, Stefano Rusconi, Francesca Montagnani, Elena Bargagli, Federico Franchi, Maria Antonietta Mazzei, Luca Cantarini, Danilo Tacconi, Marco Feri, Raffaele Scala, Genni Spargi, Cesira Nencioni, Maria Bandini, Gian Piero Caldarelli, Anna Canaccini, Agostino Ognibene, Antonella D’Arminio Monforte, Massimo Girardis, Andrea Antinori, Daniela Francisci, Elisabetta Schiaroli, Pier Giorgio Scotton, Sandro Panese, Renzo Scaggiante, Matteo Della Monica, Mario Capasso, Giuseppe Fiorentino, Marco Castori, Filippo Aucella, Antonio Di Biagio, Luca Masucci, Serafina Valente, Marco Mandalà, Patrizia Zucchi, Ferdinando Giannattasio, Domenico A. Coviello, Cristina Mussini, Luisa Tavecchia, Lia Crotti, Marco Rizzi, Maria Teresa La Rovere, Simona Sarzi-Braga, Maurizio Bussotti, Sabrina Ravaglia, Rosangela Artuso, Antonio Perrella, Davide Romani, Paola Bergomi, Emanuele Catena, Antonella Vincenti, Claudio Ferri, Davide Grassi, Gloria Pessina, Mario Tumbarello, Massimo Di Pietro, Ravaglia Sabrina, Sauro Luchi, Simone Furini, Simona Dei, Mattia Cordioli, Sara Pigazzini, Elisa Benetti, Nicola Picchiotti, Maurizio Sanarico, Stefano Ceri, Pietro Pinoli, Francesco Raimondi, Filippo Biscarini, Alessandra Stella, Kristina Zguro, Katia Capitani, Lindokuhle Nkambule, Marco Tanfoni, Chiara Fallerini, Sergio Daga, Margherita Baldassarri, Francesca Fava, Elisa Frullanti, Floriana Valentino, Gabriella Doddato, Annarita Giliberti, Rossella Tita, Sara Amitrano, Mirella Bruttini, Susanna Croci, Ilaria Meloni, Maria Antonietta Mencarelli, Caterina Lo Rizzo, Anna Maria Pinto, Giada Beligni, Andrea Tommasi, Laura Di Sarno, Maria Palmieri, Miriam Lucia Carriero, Diana Alaverdian, Stefano Busani, Raffaele Bruno, Marco Vecchia, Mary Ann Belli, Stefania Mantovani, Serena Ludovisi, Eugenia Quiros-Roldan, Melania Degli Antoni, Isabella Zanella, Matteo Siano, Arianna Emiliozzi, Massimiliano Fabbiani, Barbara Rossetti, Laura Bergantini, Miriana D’Alessandro, Paolo Cameli, David Bennett, Federico Anedda, Simona Marcantonio, Sabino Scolletta, Susanna Guerrini, Edoardo Conticini, Bruno Frediani, Chiara Spertilli, Alice Donati, Luca Guidelli, Marta Corridi, Leonardo Croci, Paolo Piacentini, Elena Desanctis, Silvia Cappelli, Agnese Verzuri, Valentina Anemoli, Alessandro Pancrazzi, Maria Lorubbio, Federica Gaia Miraglia, Sophie Venturelli, Andrea Cossarizza, Alessandra Vergori, Arianna Gabrieli, Agostino Riva, Francesco Paciosi, Francesca Andretta, Francesca Gatti, Saverio Giuseppe Parisi, Stefano Baratti, Carmelo Piscopo, Roberta Russo, Immacolata Andolfo, Achille Iolascon, Massimo Carella, Giuseppe Merla, Gabriella Maria Squeo, Pamela Raggi, Carmen Marciano, Rita Perna, Matteo Bassetti, Maurizio Sanguinetti, Alessia Giorli, Lorenzo Salerni, Pierpaolo Parravicini, Elisabetta Menatti, Tullio Trotta, Gabriella Coiro, Fabio Lena, Enrico Martinelli, Sandro Mancarella, Chiara Gabbi, Franco Maggiolo, Diego Ripamonti, Tiziana Bachetti, Claudia Suardi, Gianfranco Parati, Giordano Bottà, Paolo Di Domenico, Ilaria Rancan, Francesco Bianchi, Riccardo Colombo, Chiara Barbieri, Donatella Acquilini, Elena Andreucci, Francesco Vladimiro Segala, Giusy Tiseo, Marco Falcone, Mirjam Lista, Monica Poscente, Oreste De Vivo, Paola Petrocelli, Alessandra Guarnaccia, Silvia Baroni, Caroline Hayward, David J. Porteous, Chloe Fawns-Ritchie, Anne Richmond, Archie Campbell, David A. van Heel, Karen A. Hunt, Richard C. Trembath, Qin Qin Huang, Hilary C. Martin, Dan Mason, Bhavi Trivedi, John Wright, Sarah Finer, Shaheen Akhtar, Mohammad Anwar, Elena Arciero, Samina Ashraf, Gerome Breen, Raymond Chung, Charles J. Curtis, Maharun Chowdhury, Grainne Colligan, Panos Deloukas, Ceri Durham, Sarah Finer, Chris Griffiths, Qin Qin Huang, Matt Hurles, Karen A. Hunt, Shapna Hussain, Kamrul Islam, Ahsan Khan, Amara Khan, Cath Lavery, Sang Hyuck Lee, Robin Lerner, Daniel MacArthur, Bev MacLaughlin, Hilary Martin, Dan Mason, Shefa Miah, Bill Newman, Nishat Safa, Farah Tahmasebi, Richard C. Trembath, Bhavi Trivedi, David A. van Heel, John Wright, Christopher J. Griffiths, Albert V. Smith, Andrew P. Boughton, Kevin W. Li, Jonathon LeFaive, Aubrey Annis, Mari E. K. Niemi, Ahmadreza Niavarani, Rasoul Aliannejad, Mattia Cordioli, Lindokuhle Nkambul, Bahareh Sharififard, Ali Amirsavadkouhi, Zeinab Naderpour, Hengameh Ansari Tadi, Afshar Etemadi Aleagha, Saeideh Ahmadi, Seyed Behrooz Mohseni Moghaddam, Alireza Adamsara, Morteza Saeedi, Hamed Abdollahi, Abdolmajid Hosseini, Pajaree Chariyavilaskul, Watsamon Jantarabenjakul, Nattiya Hirankarn, Monpat Chamnanphon, Thitima B. Suttichet, Vorasuk Shotelersuk, Monnat Pongpanich, Chureerat Phokaew, Wanna Chetruengchai, Opass Putchareon, Pattama Torvorapanit, Thanyawee Puthanakit, Pintip Suchartlikitwong, Voraphoj Nilaratanakul, Pimpayao Sodsai, Ben M. Brumpton, Kristian Hveem, Cristen Willer, Brooke Wolford, Wei Zhou, Tormod Rogne, Erik Solligard, Bjørn Olav Åsvold, Lude Franke, Marike Boezen, Patrick Deelen, Annique Claringbould, Esteban Lopera, Robert Warmerdam, Judith. M. Vonk, Irene van Blokland, Pauline Lanting, Anil P. S. Ori, Yen-Chen Anne Feng, Josep Mercader, Scott T. Weiss, Elizabeth W. Karlson, Jordan W. Smoller, Shawn N. Murphy, James B. Meigs, Ann E. Woolley, Robert C. Green, Emma F. Perez, Brooke Wolford, Sebastian Zöllner, Jiongming Wang, Andrew Beck, Laura G. Sloofman, Steven Ascolillo, Robert P. Sebra, Brett L. Collins, Tess Levy, Joseph D. Buxbaum, Stuart C. Sealfon, Shea J. Andrews, Daniel M. Jordan, Ryan C. Thompson, Kyle Gettler, Kumardeep Chaudhary, Gillian M. Belbin, Michael Preuss, Clive Hoggart, Sam Choi, Slayton J. Underwood, Irene Salib, Bari Britvan, Katherine Keller, Lara Tang, Michael Peruggia, Liam L. Hiester, Kristi Niblo, Alexandra Aksentijevich, Alexander Labkowsky, Avrohom Karp, Menachem Zlatopolsky, Marissa Zyndorf, Alexander W. Charney, Noam D. Beckmann, Eric E. Schadt, Noura S. Abul-Husn, Judy H. Cho, Yuval Itan, Eimear E. Kenny, Ruth J. F. Loos, Girish N. Nadkarni, Ron Do, Paul O’Reilly, Laura M. Huckins, Manuel A. R. Ferreira, Goncalo R. Abecasis, Joseph B. Leader, Michael N. Cantor, Anne E. Justice, Dave J. Carey, Geetha Chittoor, Navya Shilpa Josyula, Jack A. Kosmicki, Julie E. Horowitz, Aris Baras, Matthew C. Gass, Ashish Yadav, Tooraj Mirshahi, Jouke Jan Hottenga, Meike Bartels, Eco E. J. C. de geus, Michel M. G. Nivard, Anurag Verma, Marylyn D. Ritchie, Daniel Rader, Binglan Li, Shefali S. Verma, Anastasia Lucas, Yuki Bradford, Malak Abedalthagafi, Manal Alaamery, Abdulraheem Alshareef, Mona Sawaji, Salam Massadeh, Abdulaziz AlMalik, Saleh Alqahtani, Dona Baraka, Fawz Al Harthi, Ebtehal Alsolm, Leen Abu Safieh, Albandary M. Alowayn, Fatimah Alqubaishi, Amal Al Mutairi, Serghei Mangul, Mansour Almutairi, Nora Aljawini, Nour Albesher, Yaseen M. Arabi, Ebrahim S. Mahmoud, Amin K. Khattab, Roaa T. Halawani, Ziab Z. Alahmadey, Jehad K. Albakri, Walaa A. Felemban, Bandar A. Suliman, Rana Hasanato, Laila Al-Awdah, Jahad Alghamdi, Deema AlZahrani, Sameera AlJohani, Hani Al-Afghani, Nouf AlDhawi, Hadeel AlBardis, Sarah Alkwai, Moneera Alswailm, Faisal Almalki, Maha Albeladi, Iman Almohammed, Eman Barhoush, Anoud Albader, Sara Alotaibi, Bader Alghamdi, Junghyun Jung, Mohammad S. fawzy, May Alrashed, Mari E. K. Niemi, Hugo Zeberg, Mattia Cordioli, Sara Pigazzini, Lindo Nkambul, Robert Frithiof, Michael Hultström, Miklos Lipcsey, Nicolas Tardif, Olav Rooyackers, Jonathan Grip, Tomislav Maricic, Øyvind Helgeland, Per Magnus, Lill-Iren S. Trogstad, Yunsung Lee, Jennifer R. Harris, Massimo Mangino, Tim D. Spector, Duncan Emma, Loukas Moutsianas, Mark J. Caulfield, Richard H. Scott, Athanasios Kousathanas, Dorota Pasko, Susan Walker, Alex Stuckey, Christopher A. Odhams, Daniel Rhodes, Tom Fowler, Augusto Rendon, Georgia Chan, Prabhu Arumugam, Tomoko Nakanishi, Konrad J. Karczewski, Alicia R. Martin, Daniel J. Wilson, Chris C. A. Spencer, Derrick W. Crook, David H. Wyllie, Anne Marie O’Connell, J. Brent Richards, Guillaume Butler-Laporte, Vincenzo Forgetta, Elizabeth G. Atkinson, Masahiro Kanai, Kristin Tsuo, Nikolas Baya, Patrick Turley, Rahul Gupta, Raymond K. Walters, Duncan S. Palmer, Gopal Sarma, Matthew Solomonson, Nathan Cheng, Wenhan Lu, Claire Churchhouse, Jacqueline I. Goldstein, Daniel King, Wei Zhou, Cotton Seed, Mark J. Daly, Benjamin M. Neale, Hilary Finucane, Sam Bryant, F. Kyle Satterstrom, Gavin Band, Sarah G. Earle, Shang-Kuan Lin, Nicolas Arning, Nils Koelling, Jacob Armstrong, Justine K. Rudkin, Shawneequa Callier, Sam Bryant, Caroline Cusick, Nicole Soranzo, Jing Hua Zhao, John Danesh, Emanuele Di Angelantonio, Adam S. Butterworth, Yan V. Sun, Jennifer E. Huffman, Kelly Cho, Christopher J. O’Donnell, Phil Tsao, J. Michael Gaziano, Gina Peloso, Yuk-Lam Ho, Sandra P. Smieszek, Christos Polymeropoulos, Vasilios Polymeropoulos, Mihael H. Polymeropoulos, Bartlomiej P. Przychodzen, Israel Fernandez-Cadenas, Anna M. Planas, Jordi Perez-Tur, Laia Llucià-Carol, Natalia Cullell, Elena Muiño, Jara Cárcel-Márquez, Marta L. DeDiego, Lara Lloret Iglesias, Alex Soriano, Veronica Rico, Daiana Agüero, Josep L. Bedini, Francisco Lozano, Carlos Domingo, Veronica Robles, Francisca Ruiz-Jaén, Leonardo Márquez, Juan Gomez, Eliecer Coto, Guillermo M. Albaiceta, Marta García-Clemente, David Dalmau, Maria J. Arranz, Beatriz Dietl, Alex Serra-Llovich, Pere Soler, Roger Colobrán, Andrea Martín-Nalda, Alba Parra Martínez, David Bernardo, Silvia Rojo, Aida Fiz-López, Elisa Arribas, Paloma de la Cal-Sabater, Tomás Segura, Esther González-Villa, Gemma Serrano-Heras, Joan Martí-Fàbregas, Elena Jiménez-Xarrié, Alicia de Felipe Mimbrera, Jaime Masjuan, Sebastian García-Madrona, Anna Domínguez-Mayoral, Joan Montaner Villalonga, Paloma Menéndez-Valladares, Daniel I. Chasman, Howard D. Sesso, JoAnn E. Manson, Julie E. Buring, Paul M. Ridker, Giulianini Franco, Andrea Ganna, Tomoko Nakanishi, J. Brent Richards, Marco Gori, Marike Boezen, Lea Davis, Sulggi Lee, James Priest, Vijay G. Sankaran, David van Heel, Les Biesecker, V. Eric Kerchberger, J. Kenneth Baillie, Benjamin M. Neale, Mark Daly, Andrea Ganna

**Affiliations:** 1 Yale University, New Haven, CT USA; 2 Institute for Molecular Medicine Finland (FIMM), Univerisity of Helsinki, Helsinki, Finland; 3 Broad Institute of MIT and Harvard, Cambridge, MA USA; 4 Massachusetts General Hospital, Broad Institute of MIT and Harvard, Cambridge, MA USA; 5 Analytic and Translational Genetics Unit, Massachusetts General Hospital, Boston, MA USA; 6 Icahn School of Medicine at Mount Sinai, New York, NY USA; 7 Institute for Molecular Medicine Finland (FIMM), University of Helsinki, Helsinki, Finland; 8 Icahn School of Medicine at Mount Sinai, Genetics and Genomic Sciences, York City, NY USA; 9 Centre for Bioinformatics and Data Analysis, Medical University of Bialystok, Bialystok, Poland; 10 University of Michigan, Ann Arbor, MI USA; 11 Ancestry, Lehi, UT USA; 12 Institute for Molecular Medicine Finland (FIMM), Helsinki, Finland; 13 University of Liege, GIGA-Institute, Liège, Belgium; 14 CHC Mont-Légia, Liège, Belgium; 15 5BHUL (Liège Biobank), CHU of Liège, Liège, Belgium; 16 Institute for Molecular Medicine Finland, University of Helsinki, Helsinki, Finland; 17 Analytic & Translational Genetics Unit, Massachusetts General Hospital, Boston, MA USA; 18 Stanley Center for Psychiatric Research, Broad Institute of MIT and Harvard, Cambridge, MA USA; 19 Program in Medical and Population Genetics, Broad Institute of MIT and Harvard, Cambridge, MA USA; 20 CHU of Liege, Liège, Belgium; 21 University of Liege, Liège, Belgium; 22 Department of Human Genetics, McGill University, Montreal, Quebec Canada; 23 Lady Davis Institute, Jewish General Hospital, McGill University, Montreal, Quebec Canada; 24 Kyoto-McGill International Collaborative School in Genomic Medicine, Graduate School of Medicine, Kyoto University, Kyoto, Japan; 25 Research Fellow, Japan Society for the Promotion of Science, Tokyo, Japan; 26 McGill Genome Centre and Department of Human Genetics, McGill University, Montreal, Quebec Canada; 27 Department of Human Genetics, Epidemiology, Biostatistics and Occupational Health, McGill University, Montreal, Quebec Canada; 28 Department of Twin Research, King’s College London, London, UK; 29 Department of Epidemiology, Biostatistics and Occupational Health, McGill University, Montréal, Québec Canada; 30 Department of Emergency Medicine, McGill University, Montreal, Quebec Canada; 31 Emergency Department, Jewish General Hospital, McGill University, Montreal, Quebec Canada; 32 McGill AIDS Centre, Department of Microbiology and Immunology, Lady Davis Institute for Medical Research, Jewish General Hospital, McGill University, Montreal, Quebec Canada; 33 McGill Centre for Viral Diseases, Lady Davis Institute, Department of Infectious Disease, Jewish General Hospital, Montreal, Quebec Canada; 34 Research Centre of the Centre Hospitalier de l’Université de Montréal, Montreal, Canada; 35 Department of Medicine, Research Centre of the Centre Hospitalier de l’Université de Montréal, Montreal, Canada; 36 Department of Medicine, Université de Montréal, Montreal, Canada; 37 Department of Medicine and Human Genetics, McGill University, Montreal, Quebec Canada; 38 Department of Intensive Care, Research Centre of the Centre Hospitalier de l’Université de Montréal, Montreal, Quebec Canada; 39 Division of Infectious Diseases, Research Centre of the Centre Hospitalier de l’Université de Montréal, Montreal, Quebec Canada; 40 MRC Human Genetics Unit, Institute of Genetics and Cancer, University of Edinburgh, Western General Hospital, Edinburgh, UK; 41 Center for Applied Genomics, Children’s Hospital of Philadelphia, Philadelphia, PA USA; 42 Department of Pediatrics, Perelman School of Medicine, University of Pennsylvania, Philadelphia, PA USA; 43 Vanderbilt University Medical Center, Nashville, TN USA; 44 Centre for Genomic and Experimental Medicine, Institute of Genetics and Cancer, University of Edinburgh, Western General Hospital, Edinburgh, UK; 45 Usher Institute, University of Edinburgh, Nine, Edinburgh Bioquarter, Edinburgh, UK; 46 University of Texas Health, Houston, TX USA; 47 Department of Psychology, University of Edinburgh, Edinburgh, UK; 48 University of North Carolina at Chapel Hill, Chapel Hill, NC USA; 49 Center for Applied Genomics, The Children’s Hospital of Philadelphia, Philadelphia, PA USA; 50 Division of Human Genetics, Department of Pediatrics, The Perelman School of Medicine, University of Pennsylvania, Philadelphia, PA USA; 51 Divisions of Human Genetics and Pulmonary Medicine, Department of Pediatrics, The Perelman School of Medicine, University of Pennsylvania, Philadelphia, PA USA; 52 Faculty of Medicine, University of Iceland, Reykjavik, Iceland; 53 University of Colorado - Anschutz Medical Campus, Aurora, CO USA; 54 MRC Human Genetics Unit, Institute of Genetics and Cancer, University of Edinburgh, Western General Hospital, Edinburgh, UK; 55 Centre for Global Health Research, Usher Institute, University of Edinburgh, Teviot Place, Edinburgh, UK; 56 The Roslin Institute, The Royal (Dick) School of Veterinary Studies, University of Edinburgh, Edinburgh, UK; 57 Department of Clinical Pathology, Faculty of Medicine, Mansoura University, Mansoura, Egypt; 58 Department of Biosciences, School of Science and Technology, Nottingham Trent University, Nottingham, UK; 59 Genolier Innovation Network and Hub, Swiss Medical Network, Genolier Healthcare Campus, Genolier, Switzerland; 60 Department of Pediatrics, Faculty of Medicine, Mansoura University, Mansoura, Egypt; 61 Department of Clinical Pathology, Faculty of Medicine, Tanta University, Tanta, Egypt; 62 Stanley Center for Psychiatric Research & Program in Medical and Population Genetics, Broad Institute of MIT and Harvard, Cambridge, MA USA; 63 Chest Department, Faculty of Medicine, Mansoura University, Mansoura, Egypt; 64 Anesthesia, Surgical Intensive Care & Pain Management Department, Faculty of Medicine, Tanta University, Tanta, Egypt; 65 Department of Medical Biochemistry, Faculty of Medicine, Mansoura University, Mansoura, Egypt; 66 Department of Surgery, Faculty of Medicine, Mansoura University, Mansoura, Egypt; 67 Department of Tropical Medicine, Faculty of Medicine, Mansoura University, Mansoura, Egypt; 68 pediatric and neonatology, Kafr Elzayat General Hospital, Kafr El-Zayat, Egypt; 69 Chest Department, Faculty of Medicine, Tanta University, Tanta, Egypt; 70 Pediatrics Department, Faculty of Medicine, Tanta University, Tanta, Egypt; 71 Department of Anaethesia and Critical Care, Faculty of Medicine, Mansoura University, Mansoura, Egypt; 72 Department of Internal Medicine, Faculty of Medicine, Tanta University, Tanta, Egypt; 73 Faculty of Science, Tanta University, Tanta, Egypt; 74 Department of Internal Medicine, Erasmus MC Rotterdam, Rotterdam, The Netherlands; 75 Department of Epidemiology, Erasmus MC Rotterdam, Rotterdam, The Netherlands; 76 Estonian Genome Centre, Institute of Genomics, University of Tartu, Tartu, Estonia; 77 SYNLAB Estonia, University of Tartu, Tartu, Estonia; 78 Kuressaare Hospital, Kuressaare, Estonia; 79 University of Tartu, Tartu, Estonia; 80 Institute of Biomedicine and Translational Medicine, University of Tartu, Tartu, Estonia; 81 West Tallinn Central Hospital, Tallinn, Estonia; 82 University of Tartu, Tartu University Hospital, Tartu, Estonia; 83 Estonian Health Insurance Fund, Tallinn, Estonia; 84 Tartu University Hospital, Tartu, Estonia; 85 Department of Health Sciences, University of Leicester, Leicester, UK; 86 Leicester NIHR Biomedical Research Centre, Leicester, UK; 87 Department of Respiratory Sciences, University of Leicester, Leicester, UK; 88 Department of Genetics and Genome Biology, University of Leicester, Leicester, UK; 89 FinnGen, Helsinki, Finland; 90 Institute for Molecular Medicine Finland (FIMM), HiLIFE, University of Helsinki, Helsinki, Finland; 91 Public Health, Faculty of Medicine, University of Helsinki, Helsinki, Finland; 92 Finnish Institute for Health and Welfare (THL), Helsinki, Finland; 93 University of Helsinki, Faculty of Medicine, Clinical and Molecular Metabolism Research Program, Helsinki, Finland; 94 Institute of Molecular and Clinical Ophthalmology Basel (IOB), Basel, Switzerland; 95 Department of Ophthalmology, University of Basel, Basel, Switzerland; 96 Infectious Diseases Service, Department of Medicine, University Hospital and University of Lausanne, Lausanne, Switzerland; 97 Infectious Diseases Service, Department of Medicine, University Hospital, University of Lausanne, Lausanne, Switzerland; 98 Centre for Primary Care and Public Health, University of Lausanne, Lausanne, Switzerland; 99 Division of Infectious Diseases and Hospital Epidemiology, Cantonal Hospital St Gallen, St Gallen, Switzerland; 100 Division of Intensive Care, Geneva University Hospitals and the University of Geneva Faculty of Medicine, Geneva, Switzerland; 101 Infectious Disease Service, Department of Internal Medicine, Geneva University Hospital, Geneva, Switzerland; 102 Clinique de Médecine et spécialités, Infectiologie, HFR-Fribourg, Fribourg, Switzerland; 103 Infectious Diseases Division, University Hospital Centre of the Canton of Vaud, Hospital of Valais, Sion, Switzerland; 104 Functional Host Genomics of Infectious Diseases, University Hospital and University of Lausanne, Lausanne, Switzerland; 105 Registry COVID, University Hospital and University of Lausanne, Lausanne, Switzerland; 106 Pneumonia prediction using lung ultrasound, University Hospital and University of Lausanne, Lausanne, Switzerland; 107 Center for Primary Care and Public Health (Unisanté), University of Lausanne, Lausanne, Switzerland; 108 Covid-19 Risk Prediction in Swiss ICUs-Trial, Division of Infectious Diseases and Hospital Epidemiology, Cantonal Hospital St Gallen, St Gallen, Switzerland; 109 GCAT-Genomes for Life, Germans Trias i Pujol Health Sciences Research Institute (IGTP), Badalona, Spain; 110 Catalan Institute of Oncology, Bellvitge Biomedical Research Institute, Consortium for Biomedical Research in Epidemiology and Public Health and University of Barcelona, Barcelona, Spain; 111 ISGlobal, Barcelona, Spain; 112 IMIM (Hospital del Mar Medical Research Institute), Barcelona, Spain; 113 Universitat Pompeu Fabra (UPF), Barcelona, Spain; 114 CIBER Epidemiología y Salud Pública (CIBERESP), Madrid, Spain; 115 Barcelona Supercomputing Center, Centro Nacional de Supercomputación (BSC-CNS), Life & Medical Sciences, Barcelona, Spain; 116 Diabetes Unit and Center for Genomic Medicine, Massachusetts General Hospital, Boston, MA USA; 117 Harvard Medical School, Boston, Massachusetts USA; 118 Barcelona Supercomputing Center, Centro Nacional de Supercomputación (BSC-CNS), Life & Medical Sciences, Barcelona, Spain; 119 University of Siena, DIISM-SAILAB, Siena, Italy; 120 Université Côte d’Azur, Inria, CNRS, I3S, Maasai, Nice, France; 121 Medical Genetics, University of Siena, Siena, Italy; 122 Genetica Medica, Azienda Ospedaliero-Universitaria Senese, Siena, Italy; 123 Med Biotech Hub and Competence Center, Department of Medical Biotechnologies, University of Siena, Siena, Italy; 124 Division of Infectious Diseases and Immunology, Department of Medical Sciences and Infectious Diseases, Fondazione IRCCS Policlinico San Matteo, Pavia, Italy; 125 Department of Internal Medicine and Therapeutics, University of Pavia, Pavia, Italy; 126 Department of Infectious and Tropical Diseases, University of Brescia and ASST Spedali Civili Hospital, Brescia, Italy; 127 Chirurgia Vascolare, Ospedale Maggiore di Crema, Crema, Italy; 128 III Infectious Diseases Unit, ASST-FBF-Sacco, Milan, Italy; 129 Department of Biomedical and Clinical Sciences Luigi Sacco, University of Milan, Milan, Italy; 130 Dept of Specialized and Internal Medicine, Tropical and Infectious Diseases Unit, Azienda Ospedaliera Universitaria Senese, Siena, Italy; 131 Unit of Respiratory Diseases and Lung Transplantation, Department of Internal and Specialist Medicine, University of Siena, Siena, Italy; 132 Dept of Emergency and Urgency, Medicine, Surgery and Neurosciences, Unit of Intensive Care Medicine, Siena University Hospital, Siena, Italy; 133 Department of Medical, Surgical and Neuro Sciences and Radiological Sciences, Unit of Diagnostic Imaging, University of Siena, Siena, Italy; 134 Rheumatology Unit, Department of Medicine, Surgery and Neurosciences, University of Siena, Policlinico Le Scotte, Siena, Italy; 135 Department of Specialized and Internal Medicine, Infectious Diseases Unit, San Donato Hospital Arezzo, Arezzo, Italy; 136 Dept of Emergency, Anesthesia Unit, San Donato Hospital, Arezzo, Italy; 137 Department of Specialized and Internal Medicine, Pneumology Unit and UTIP, San Donato Hospital, Arezzo, Italy; 138 Department of Emergency, Anesthesia Unit, Misericordia Hospital, Grosseto, Italy; 139 Department of Specialized and Internal Medicine, Infectious Diseases Unit, Misericordia Hospital, Grosseto, Italy; 140 Department of Preventive Medicine, Azienda USL Toscana Sud Est, Arezzo, Italy; 141 Clinical Chemical Analysis Laboratory, Misericordia Hospital, Grosseto, Italy; 142 Territorial Scientific Technician Department, Azienda USL Toscana Sud Est, Arezzo, Italy; 143 Clinical Chemical Analysis Laboratory, San Donato Hospital, Arezzo, Italy; 144 Department of Health Sciences, Clinic of Infectious Diseases, ASST Santi Paolo e Carlo, University of Milan, Milan, Italy; 145 Department of Anesthesia and Intensive Care, University of Modena and Reggio Emilia, Modena, Italy; 146 HIV/AIDS Department, National Institute for Infectious Diseases, IRCCS, Lazzaro Spallanzani, Rome, Italy; 147 Infectious Diseases Clinic, Department of Medicine 2, Azienda Ospedaliera di Perugia and University of Perugia, Santa Maria Hospital, Perugia, Italy; 148 Infectious Diseases Clinic, “Santa Maria” Hospital, University of Perugia, Perugia, Italy; 149 Department of Infectious Diseases, Treviso Hospital, Treviso, Italy; 150 Clinical Infectious Diseases, Mestre Hospital, Venezia, Italy; 151 Infectious Diseases Clinic, Belluno Hospital, Viale Europa, Belluno, Italy; 152 Medical Genetics and Laboratory of Medical Genetics Unit, A.O.R.N. “Antonio Cardarelli”, Naples, Italy; 153 Department of Molecular Medicine and Medical Biotechnology, University of Naples Federico II, Naples, Italy; 154 CEINGE Biotecnologie Avanzate, Naples, Italy; 155 IRCCS SDN, Naples, Italy; 156 Unit of Respiratory Physiopathology, AORN dei Colli, Monaldi Hospital, Naples, Italy; 157 Division of Medical Genetics, Fondazione IRCCS Casa Sollievo della Sofferenza Hospital, San Giovanni Rotondo, Italy; 158 Department of Medical Sciences, Fondazione IRCCS Casa Sollievo della Sofferenza Hospital, San Giovanni Rotondo, Italy; 159 Infectious Diseases Clinic, Policlinico San Martino Hospital, IRCCS for Cancer Research, Genova, Italy; 160 Microbiology, Fondazione Policlinico Universitario Agostino Gemelli IRCCS, Catholic University of Medicine, Rome, Italy; 161 Department of Laboratory Sciences and Infectious Diseases, Fondazione Policlinico Universitario A. Gemelli IRCCS, Rome, Italy; 162 Department of Cardiovascular Diseases, University of Siena, Siena, Italy; 163 Otolaryngology Unit, University of Siena, Siena, Italy; 164 Department of Internal Medicine, ASST Valtellina e Alto Lario, Sondrio, Italy; 165 First Aid Department, Luigi Curto Hospital, Polla, Salerno, Italy; 166 U.O.C. Laboratorio di Genetica Umana, IRCCS Istituto G. Gaslini, Genova, Italy; 167 Infectious Diseases Clinics, University of Modena and Reggio Emilia, Modena, Italy; 168 U.O.C. Medicina, ASST Nord Milano, Ospedale Bassini, Cinisello Balsamo, Milan, Italy; 169 Department of Cardiovascular, Neural and Metabolic Sciences, Istituto Auxologico Italiano, IRCCS, San Luca Hospital, Milan, Italy; 170 Department of Medicine and Surgery, University of Milano-Bicocca, Milan, Italy; 171 Istituto Auxologico Italiano, IRCCS, Center for Cardiac Arrhythmias of Genetic Origin, Milan, Italy; 172 Istituto Auxologico Italiano, IRCCS, Laboratory of Cardiovascular Genetics, Milan, Italy; 173 Unit of Infectious Diseases, ASST Papa Giovanni XXIII Hospital, Bergamo, Italy; 174 Department of Cardiology, Istituti Clinici Scientifici Maugeri IRCCS, Institute of Montescano, Pavia, Italy; 175 Istituti Clinici Scientifici Maugeri, IRCCS, Department of Cardiac Rehabilitation, Institute of Tradate (VA), Pavia, Italy; 176 Cardiac Rehabilitation Unit, Fondazione Salvatore Maugeri, IRCCS, Scientific Institute of Milan, Milan, Italy; 177 IRCCS C. Mondino Foundation, Pavia, Italy; 178 Medical Genetics Unit, Meyer Children’s University Hospital, Florence, Italy; 179 Department of Medicine, Pneumology Unit, Misericordia Hospital, Grosseto, Italy; 180 Department of Preventive Medicine, Azienda USL Toscana Sud Est, Tuscany, Italy; 181 Department of Anesthesia and Intensive Care Unit, ASST Fatebenefratelli Sacco, Luigi Sacco Hospital, Polo Universitario, University of Milan, Milan, Italy; 182 Infectious Disease Unit, Hospital of Massa, Massa, Italy; 183 Department of Clinical Medicine, Public Health, Life and Environment Sciences, University of L’Aquila, L’Aquila, Italy; 184 UOSD Laboratorio di Genetica Medica - ASL Viterbo, San Lorenzo, Italy; 185 Department of Medical Sciences, Infectious and Tropical Diseases Unit, Azienda Ospedaliera Universitaria Senese, Siena, Italy; 186 Unit of Infectious Diseases, S.M. Annunziata Hospital, Florence, Italy; 187 IRCCS Mondino Foundation, Pavia, Italy; 188 Infectious Disease Unit, Hospital of Lucca, Lucca, Italy; 189 Health Management, Azienda USL Toscana Sudest, Tuscany, Italy; 190 Department of Mathematics, University of Pavia, Pavia, Italy; 191 Independent Researcher, Milan, Italy; 192 Department of Electronics, Information and Bioengineering (DEIB), Politecnico di Milano, Milano, Italy; 193 Scuola Normale Superiore, Pisa, Italy; 194 CNR-Consiglio Nazionale delle Ricerche, Istituto di Biologia e Biotecnologia Agraria (IBBA), Milano, Italy; 195 Core Research Laboratory, ISPRO, Florence, Italy; 196 Division of Infectious Diseases and Immunology, Fondazione IRCCS Policlinico San Matteo, Pavia, Italy; 197 Fondazione IRCCS Ca’ Granda Ospedale Maggiore Policlinico, Milano, Italy; 198 Department of Molecular and Translational Medicine, University of Brescia, Brescia, Italy; 199 Clinical Chemistry Laboratory, Cytogenetics and Molecular Genetics Section, Diagnostic Department, ASST Spedali Civili di Brescia, Brescia, Italy; 200 Department of Medical and Surgical Sciences for Children and Adults, University of Modena and Reggio Emilia, Modena, Italy; 201 Department of Molecular Medicine, University of Padova, Padua, Italy; 202 Laboratory of Regulatory and Functional Genomics, Fondazione IRCCS Casa Sollievo della Sofferenza, San Giovanni Rotondo (Foggia), Foggia, Italy; 203 Clinical Trial Office, Fondazione IRCCS Casa Sollievo della Sofferenza Hospital, San Giovanni Rotondo, Italy; 204 Department of Health Sciences, University of Genova, Genova, Italy; 205 Oncologia Medica e Ufficio Flussi Sondrio, Sondrio, Italy; 206 Local Health Unit-Pharmaceutical Department of Grosseto, Toscana Sud Est Local Health Unit, Grosseto, Italy; 207 Department of Respiratory Diseases, Azienda Ospedaliera di Cremona, Cremona, Italy; 208 Direzione Scientifica, Istituti Clinici Scientifici Maugeri IRCCS, Pavia, Italy; 209 Fondazione per la ricerca Ospedale di Bergamo, Bergamo, Italy; 210 Allelica Inc, New York, NY USA; 211 Department of Clinical and Experimental Medicine, Infectious Diseases Unit, University of Pisa, Pisa, Italy; 212 Infectious Disease Unit, Santo Stefano Hospital, AUSL Toscana Centro, Prato, Italy; 213 Clinic of Infectious Diseases, Catholic University of the Sacred Heart, Rome, Italy; 214 Medical Genetics, University of Siena, Siena, Italy; 215 Infectious Disease Unit, Hospital of Lucca, Lucca, Italy; 216 Department of Diagnostic and Laboratory Medicine, Institute of Biochemistry and Clinical Biochemistry, Fondazione Policlinico Universitario A. Gemelli IRCCS, Catholic University of the Sacred Heart, Rome, Italy; 217 MRC Human Genetics Unit, IGC, University of Edinburgh, Edinburgh, UK; 218 Medical Genetics Section, Centre for Genomic and Experimental Medicine, IGC, University of Edinburgh, Edinburgh, UK; 219 Generation Scotland, Centre for Genomic and Experimental Medicine, IGC, University of Edinburgh, Edinburgh, UK; 220 Blizard Institute, Queen Mary University of London, London, UK; 221 School of Basic and Medical Biosciences, Faculty of Life Sciences and Medicine, King’s College London, London, UK; 222 Medical and Population Genomics, Wellcome Sanger Institute, Hinxton, UK; 223 Bradford Institute for Health Research, Bradford Teaching Hospitals National Health Service (NHS) Foundation Trust, Bradford, UK; 224 Blizard Institute, Queen Mary University of London, London, UK; 225 Institute of Population Health Sciences, Queen Mary University of London, London, UK; 226 Genes & Health, Blizard Institute, Queen Mary University of London, London, UK; 227 Institute of Population Health Sciences, Queen Mary University of London, London, UK; 228 Department of Biostatistics, University of Michigan, Ann Arbor, MI USA; 229 Digestive Oncology Research Center, Digestive Disease Research Institute, Shariati Hospital, Tehran University of Medical Sciences, Tehran, Iran; 230 Department of Pulmonology, School of Medicine, Shariati Hospital, Tehran University of Medical Sciences, Tehran, Iran; 231 Department of Critical Care Medicine, Noorafshar Hospital, Tehran, Iran; 232 Department of Emergency Intensive Care Unit, School of Medicine, Shariati Hospital, Tehran University of Medical Sciences, Tehran, Iran; 233 Department of Anesthesiology, School of Medicine, Amir Alam Hospital, Tehran University of Medical Sciences, Tehran, Iran; 234 Department of Pulmonology, School of Medicine, Tehran University of Medical Sciences, Tehran, Iran; 235 Department of Pathology, Parseh Pathobiology and Genetics Laboratory, Tehran, Iran; 236 Department of Microbiology, Health and Family Research Center, NIOC Hospital, Tehran, Iran; 237 Department of Emergency Medicine, School of Medicine, Shariati Hospital, Tehran University of Medical Sciences, Tehran, Iran; 238 Department of Anesthesiology, School of Medicine, Tehran University of Medical Sciences, Tehran, Iran; 239 Department of Pathology, Faculty of Medicine, Tehran Azad University, Tehran, Iran; 240 Clinical Pharmacokinetics and Pharmacogenomics Research Unit, Faculty of Medicine, Chulalongkorn University, Bangkok, Thailand; 241 Department of Pharmacology, Faculty of Medicine, Chulalongkorn University, Bangkok, Thailand; 242 Thai Red Cross Emerging Infectious Diseases Clinical Centre, King Chulalongkorn Memorial Hospital, Bangkok, Thailand; 243 Department of Pediatrics, Faculty of Medicine, Chulalongkorn University, Bangkok, Thailand; 244 Immunology Division, Department of Microbiology, Faculty of Medicine, Chulalongkorn University, Bangkok, Thailand; 245 Center of Excellence in Immunology and Immune-mediated Diseases, Department of Microbiology, Faculty of Medicine, Chulalongkorn University, Bangkok, Thailand; 246 Clinical Pharmacokinetics and Pharmacogenomics Research Unit, Faculty of Medicine, Chulalongkorn University, Bangkok, Thailand; 247 Department of Pathology, Faculty of Medicine, Nakornnayok, Srinakharinwirot University, Bangkok, Thailand; 248 Center of Excellence for Medical Genomics, Medical Genomics Cluster, Department of Pediatrics, Faculty of Medicine, Chulalongkorn University, Bangkok, Thailand; 249 Excellence Center for Genomics and Precision Medicine, King Chulalongkorn Memorial Hospital, The Thai Red Cross Society, Bangkok, Thailand; 250 Department of Mathematics and Computer Science, Faculty of Science, Chulalongkorn University, Bangkok, Thailand; 251 Omics Sciences and Bioinfomatics Center, Faculty of Science, Chulalongkorn University, Bangkok, Thailand; 252 Research Affairs, Faculty of Medicine, Chulalongkorn University, Bangkok, Thailand; 253 Center of Excellence for Medical Genomics, Medical Genomics Cluster, Faculty of Medicine, Chulalongkorn University, Bangkok, Thailand; 254 Division of Infectious Disease, Department of Medicine, Faculty of Medicine, Chulalongkorn University, Bangkok, Thailand; 255 Center of Excellence in Pediatric Infectious Diseases and Vaccines, Chulalongkorn University, Bangkok, Thailand; 256 Department of Microbiology, Faculty of Medicine, Chulalongkorn University, Bangkok, Thailand; 257 Division of Infectious Diseases, Department of Medicine, Faculty of Medicine, Chulalongkorn University, Bangkok, Thailand; 258 Healthcare-associated Infection Research Group STAR (Special Task Force for Activating Research), Chulalongkorn University, Bangkok, Thailand; 259 K.G. Jebsen Center for Genetic Epidemiology, Department of Public Health and Nursing, NTNU, Norwegian University of Science and Technology, Trondheim, Norway; 260 HUNT Research Center, Department of Public Health and Nursing, NTNU, Norwegian University of Science and Technology, Levanger, Norway; 261 Clinic of Medicine, St. Olavs Hospital, Trondheim University Hospital, Trondheim, Norway; 262 Division of Cardiovascular Medicine, Department of Internal Medicine, University of Michigan, Ann Arbor, MI USA; 263 Department of Computational Medicine and Bioinformatics, University of Michigan, Ann Arbor, MI USA; 264 Department of Human Genetics, University of Michigan, Ann Arbor, MI USA; 265 Analytic and Translational Genetics Unit, Massachusetts General Hospital, Boston, Massachusetts USA; 266 Program in Medical and Population Genetics, Broad Institute of Harvard and MIT, Cambridge, Massachusetts USA; 267 Gemini Center for Sepsis Research, Department of Circulation and Medical Imaging, NTNU, Norwegian University of Science and Technology, Trondheim, Norway; 268 Department of Chronic Disease Epidemiology and Center for Perinatal, Pediatric and Environmental Epidemiology, Yale School of Public Health, New Haven, CT USA; 269 Clinic of Anaesthesia and Intensive Care, St. Olavs Hospital, Trondheim University Hospital, Trondheim, Norway; 270 Department of Genetics, University Medical Centre Groningen, University of Groningen, Groningen, Netherlands; 271 Department of Epidemiology, University Medical Centre Groningen, University of Groningen, Groningen, Netherlands; 272 Department of Genetics, University Medical Centre Groningen, University of Groningen / Department of Genetics, University Medical Centre Utrecht, Utrecht, The Netherlands; 273 Department of Epidemiology, University of Groningen, University Medical Center Groningen, Groningen, The Netherlands; 274 University of Groningen, University Medical Center Groningen, Department of Genetics, Groningen, The Netherlands; 275 Department of Genetics, University Medical Center Groningen, Groningen, The Netherlands; 276 Department of Psychiatry, University Medical Center Groningen, Groningen, The Netherlands; 277 Center for Genomic Medicine, Massachusetts General Hospital, Boston, MA USA; 278 Programs in Metabolism and Medical and Population Genetics, Broad Institute of MIT and Harvard, Cambridge, MA USA; 279 Channing Division of Network Medicine, Department of Medicine, Brigham and Women’s Hospital, Boston, MA USA; 280 Brigham and Women’s Hospital, Boston, MA USA; 281 Psychiatric and Neurodevelopmental Genetics Unit, Center for Genomic Medicine, Massachusetts General Hospital, Boston, MA USA; 282 Department of Neurology, Massachusetts General Hospital, Boston, MA USA; 283 Division of General Internal Medicine, Massachusetts General Hospital and Dpeartment of Medicine, Harvard Medical School and Program in Medical and Population Genetics, Broad Institute, Boston, MA USA; 284 Division of Genetics, Department of Medicine, Brigham and Women’s Hospital, Broad Institute of MIT and Harvard, Harvard Medical School, Boston, MA USA; 285 Division of Genetics, Department of Medicine, Brigham and Women’s Hospital, Boston, MA USA; 286 Seaver Autism Center for Research and Treatment, York City, NY USA; 287 Department of Psychiatry, Icahn School of Medicine, New York, NY USA; 288 Mount Sinai Clinical Intelligence Center, Department of Genetics and Genomic Sciences, Icahn School of Medicine at Mount Sinai, New York, NY USA; 289 Department of Genetics and Genomic Sciences, Icahn School of Medicine at Mount Sinai, New York, NY USA; 290 Sema4, a Mount Sinai venture, Stamford, CT USA; 291 Seaver Autism Center for Research and Treatment, Department of Psychiatry, Icahn School of Medicine at Mount Sinai, New York, NY USA; 292 Mount Sinai Clnical Intelligence Center, Charles Bronfman Institute for Personalized Medicine, New York, NY USA; 293 Department of Genetics & Genomic Sciences, Icahn School of Medicine at Mount Sinai, New York, NY USA; 294 Icahn Institute of Data Science and Genomics Technology, New York, NY USA; 295 Mount Sinai Clinical Intelligence Center, New York, NY USA; 296 Charles Bronfman Institute for Personalized Medicine, Icahn School of Medicine at Mount Sinai, New York, NY USA; 297 Institute for Genomic Health, Icahn School of Medicine at Mount Sinai, New York, NY USA; 298 The Mindich Child Health and Development Institute, Icahn School of Medicine at Mount Sinai, New York, NY USA; 299 Pamela Sklar Division of Psychiatric Genomics, Department of Psychiatry, Department of Genetic and Genomic Sciences, New York, NY USA; 300 Pamela Sklar Division of Psychiatric Genomics, Department of Psychiatry, Department of Genetic and Genomic Sciences, Icahn School of Medicine at Mount Sinai, New York, NY USA; 301 Seaver Autism Center for Research and Treatment, Department of Psychiatry, New York, NY USA; 302 Mount Sinai Clinical Intelligence Center, Department of Psychiatry, Department of Genetic and Genomic Sciences, Icahn School of Medicine at Mount Sinai, Mount Sinai, NY USA; 303 Department of Environmental Medicine and Public Health, Icahn School of Medicine at Mount Sinai, New York, NY USA; 304 The Hasso Plattner Institute of Digital Health at Mount Sinai, New York, NY USA; 305 BioMe Phenomics Center, Icahn School of Medicine at Mount Sinai, New York, NY USA; 306 Department of Medicine, Icahn School of Medicine at Mount Sinai, New York, NY USA; 307 Pamela Sklar Division of Psychiatric Genomics, Seaver Autism Center for Research and Treatment, Department of Psychiatry, Department of Genetic and Genomic Sciences, Icahn School of Medicine at Mount Sinai, New York, NY USA; 308 Regeneron Genetics Center, Tarrytown, NY USA; 309 Phenomic Analytics & Clinical Data Core, Geisinger Health System, Danville, PA USA; 310 Department of Population Health Sciences, Geisinger Health System, Danville, PA USA; 311 Department of Molecular and Functional Genomics, Geisinger Health System, Danville, PA USA; 312 Vrije Universiteit Amsterdam, Amsterdam, UK; 313 Department of Genetics, University of Pennsylvania Perelman School of Medicine, Philadelphia, PA USA; 314 Department of Biomedical Data Science, Stanford University, Stanford, CA USA; 315 Genomics Research Department, Saudi Human Genome Project, King Fahad Medical City and King Abdulaziz City for Science and Technology, Riyadh, Saudi Arabia; 316 Developmental Medicine Department, King Abdullah International Medical Research Center, King Saud Bin Abdulaziz University for Health Sciences, Ministry of National Guard- Health Affairs, Riyadh, Saudi Arabia; 317 Saudi Human Genome Project (SHGP), King Abdulaziz City for Science and Technology (KACST), Satellite Lab at King Abdulaziz Medical City (KAMC), Ministry of National Guard Health Affairs (MNG-HA), Riyadh, Saudi Arabia; 318 College of Applied Medical Sciences, Taibah University, Madina, Saudi Arabia; 319 Developmental Medicine Department, King Abdullah International Medical Research Center, King Saud Bin Abdulaziz University for Health Sciences, Ministry of National Guard Health Affairs, Riyadh, Saudi Arabia; 320 Life Science and environmental institute, King Abdulaziz City for Science and Technology, Riyadh, Saudi Arabia; 321 The Liver Transplant Unit, King Faisal Specialist Hospital and Research Centre, Riyadh, Saudi Arabia; 322 The Division of Gastroenterology and Hepatology, Johns Hopkins University, Baltimore, MD USA; 323 Department of Pathology, College of Medicine, King Saud University, Riyadh, Saudi Arabia; 324 Titus Family Department of Clinical Pharmacy, USC School of Pharmacy University of Southern California, Los Angeles, CA USA; 325 KACST-BWH Centre of Excellence for Biomedicine, Joint Centers of Excellence Program, King Abdulaziz City for Science and Technology (KACST), Riyadh, Saudi Arabia; 326 Ministry of the National Guard Health Affairs, King Abdullah International Medical Research Center and King Saud Bin Abdulaziz University for Health Sciences, Riyadh, Saudi Arabia; 327 Ohud Hospital, Ministry of Health, Madinah, Saudi Arabia; 328 Pediatric Infectious Diseases, Children’s Specialized Hospital, King Fahad Medical City, Riyadh, Saudi Arabia; 329 The Saudi Biobank, King Abdullah International Medical Research Center, King Saud bin Abdulaziz University for Health Sciences, Ministry of National Guard Health Affairs, Riyadh, Saudi Arabia; 330 Developmental Medicine Department, King Abdullah International Medical Research Center, King Saud Bin Abdulaziz University for Health Sciences, King AbdulAziz Medical City, Ministry of National Guard Health Affairs, Riyadh, Saudi Arabia; 331 Department of Pathology and Laboratory Medicine, King Abdulaziz Medical City, Ministry of National Guard Health Affairs, King Saud Bin Abdulaziz University for Health Sciences and King Abdullah International Medical Research Center, Riyadh, Saudi Arabia; 332 Laboratory Department, Security Forces Hospital, General Directorate of Medical Services, Ministry of Interior, Riyadh, Saudi Arabia; 333 King Abdulaziz City for Science and Technology (KACST), Riyadh, Saudi Arabia; 334 Department of Developmental Medicine, King Abdullah International Medical Research Center, King Saud Bin Abdulaziz University for Health Sciences, King Abdulaziz Medical City, Ministry of National Guard Health Affairs, Riyadh, Saudi Arabia; 335 Titus Family Department of Clinical Pharmacy, USC School of Pharmacy, Los Angeles, CA USA; 336 Department of Clinical Laboratory Sciences, College of Applied Medical Sciences, King Saud University, Riyadh, Saudi Arabia; 337 Department of Neuroscience, Karolinska Institutet, Stockholm, Sweden; 338 Max Planck Institute for Evolutionary Anthropology, Leipzig, Germany; 339 Stanley Center for Psychiatric Research & Program in Medical and Population Genetics, Cambridge, MA USA; 340 Anaesthesiology and Intensive Care Medicine, Department of Surgical Sciences, Uppsala University, Uppsala, Sweden; 341 Integrative Physiology, Department of Medical Cell Biology, Uppsala University, Uppsala, Sweden; 342 Hedenstierna Laboratory, CIRRUS, Anaesthesiology and Intensive Care Medicine, Department of Surgical Sciences, Uppsala University, Uppsala, Sweden; 343 Division Anesthesiology and Intensive Care, CLINTEC, Karolinska Institutet, Stockholm, Sweden; 344 Department of Genetics and Bioinformatics, Norwegian Institute of Public Health, Oslo, Norway; 345 Centre for Fertility and Health, Norwegian Institute of Public Health, Oslo, Norway; 346 Department of Method Development and Analytics, Norwegian Institute of Public Health, Oslo, Norway; 347 King’s College London, Department of Twin Research and Genetic Epidemiology, London, UK; 348 NIHR Biomedical Research Centre at Guy’s and St Thomas’ Foundation Trust, London, UK; 349 Genomics England, London, UK; 350 Queen Mary University, London, UK; 351 William Harvey Research Institute, Barts and the London School of Medicine and Dentistry, Queen Mary University of London, London, UK; 352 UCL Great Ormond Street Institute of Child Health, London, UK; 353 Genomics England, London, UK; 354 University of Cambridge, London, United Kingdom; 355 Big Data Institute, Nuffield Department of Population Health, University of Oxford, Li Ka Shing Centre for Health Information and Discovery, Old Road Campus, Oxford, UK; 356 Genomics PLC, King Charles House, Oxford, UK; 357 Nuffield Department of Medicine, Experimental Medicine Division, University of Oxford, John Radcliffe Hospital, Oxford, UK; 358 Public Health England, Field Service, Addenbrooke’s Hospital, Cambridge, UK; 359 Public Health England, Data and Analytical Services, National Infection Service, London, UK; 360 Program in Bioinformatics and Integrative Genomics, Harvard Medical School, Boston, MA USA; 361 Program in Biological and Biomedical Sciences, Harvard Medical School, Boston, MA USA; 362 Wellcome Centre for Human Genetics, University of Oxford, Roosevelt Drive, Oxford, UK; 363 Department of Clinical Research and Leadership, George Washington University, Washington, DC USA; 364 Department of Human Genetics, The Wellcome Sanger Institute, Wellcome Genome Campus, Hinxton, Cambridge, UK; 365 The National Institute for Health Research Blood and Transplant Unit in Donor Health and Genomics, University of Cambridge, Strangeways Research Laboratory, Wort’s Causeway, Cambridge, UK; 366 Department of Haematology, University of Cambridge, Cambridge Biomedical Campus, Long Road, Cambridge, UK; 367 British Heart Foundation Cardiovascular Epidemiology Unit, Department of Public Health and Primary Care, University of Cambridge, Cambridge, UK; 368 British Heart Foundation Centre of Research Excellence, University of Cambridge, Cambridge, UK; 369 National Institute for Health Research Blood and Transplant Research Unit in Donor Health and Genomics, University of Cambridge, Cambridge, UK; 370 Health Data Research UK Cambridge, Wellcome Genome Campus and University of Cambridge, Cambridge, UK; 371 Department of Human Genetics, Wellcome Sanger Institute, Hinxton, UK; 372 Department of Epidemiology, Emory University Rollins School of Public Health, Atlanta, GA USA; 373 Atlanta CA Health Care System, North Druid Hills, GA USA; 374 Center for Population Genomics, MAVERIC, VA Boston Healthcare System, Boston, MA USA; 375 MAVERIC, VA Boston Healthcare System, Boston, MA USA; 376 Stanford University, Stanford, CA USA; 377 Palo Alto VA Healthcare System, Stanford, CA USA; 378 Department of Biostatistics, Boston Univeristy School of Public Health, Boston, MA USA; 379 Vanda Pharmaceuticals Inc., Washington, DC USA; 380 Stroke Pharmacogenomics and Genetics, Biomedical Research Institute Sant Pau, Sant Pau Hospital, Barcelona, Spain; 381 Institute for Biomedical Researhc of Barcelona (IIBB), National Spanish Research Council (CSIC), Madrid, Spain; 382 Institut d’Investigacions Biomèdiques August Pi i Sunyer (IDIBAPS), Barcelona, Spain; 383 Institute of Biomedicine of Valencia (IBV), CSIC, València, Spain; 384 Network Center for Biomedical Research on Neurodegenerative Diseases (CIBERNED), València, Spain; 385 Neurology and Genetic Mixed Unit, La Fe Helath Research Institute, València, Spain; 386 Institute for Biomedical Research of Barcelona (IIBB), National Spanish Research Council (CSIC), Barcelona, Spain; 387 Department of Neurology, Hospital Universitari MútuaTerrassa, Fundació Docència i Recerca MútuaTerrassa, Terrassa, Spain; 388 Department of Molecular and Cell Biology, Centro Nacional de Biotecnología (CNB-CSIC), Campus Universidad Autónoma de Madrid, Madrid, Spain; 389 Instituto de Física de Cantabria (IFCA-CSIC), Santander, Spain; 390 Hospital Clínic, IDIBAPS, Barcelona, Spain; 391 Hospital Clínic, Barcelona, Spain; 392 Hospital Clínic, IDIBAPS, School of Medicine, University of Barcelona, Barcelona, Spain; 393 IDIBAPS, Barcelona, Spain; 394 IIBB-CSIC, Barcelona, Spain; 395 Servicio de Salud del Principado de Asturias, Oviedo, Spain; 396 Hospital Mutua de Terrassa, Terrassa, Spain; 397 Hospital Valle Hebrón, Barcelona, Spain; 398 Instituto de Biomedicina y Genética Molecular (IBGM), CSIC-Universidad de Valladolid, Valladolid, Spain; 399 Hospital Clínico Universitario de Valladolid (SACYL), Valladolid, Spain; 400 Department of Neurology, University Hospital of Albacete, Albacete, Spain; 401 Research Unit, University Hospital of Albacete, Albacete, Spain; 402 Department of Neurology, Biomedical Research Institute Sant Pau (IIB Sant Pau), Hospital de la Santa Creu i Sant Pau, Barcelona, Spain; 403 Hospital Universitario Ramón Y Cajal, IRYCIS, Madrid, Spain; 404 Institute de Biomedicine of Seville, IBiS/Hospital Universitario Virgen del Rocío/CSIC/University of Seville & Department of Neurology, Hospital Universitario Virgen Macarena, Seville, Spain; 405 Brigham and Women’s Hospital, Boston, USA; 406 Harvard Medical School, Boston, MA USA; 407 University of California San Francisco, San Francisco, CA USA; 408 Boston Children’s Hospital, Boston, MA USA; 409 National Institutes of Health, Bethesda, MD USA; 410 Intensive Care Unit, Royal Infirmary of Edinburgh, Edinburgh, UK

**Keywords:** Genome-wide association studies, Genetic variation

The COVID-19 pandemic continues to pose a major public health threat, especially in countries with low vaccination rates. To better understand the biological underpinnings of SARS-CoV-2 infection and COVID-19 severity, we formed the COVID-19 Host Genetics Initiative^1^. Here we present a genome-wide association study meta-analysis of up to 125,584 cases and over 2.5 million control individuals across 60 studies from 25 countries, adding 11 genome-wide significant loci compared with those previously identified^2^. Genes at new loci, including *SFTPD*, *MUC5B* and *ACE2*, reveal compelling insights regarding disease susceptibility and severity.

Here we present meta-analyses bringing together 60 studies from 25 countries (Fig. 1 and Supplementary Table 1) for three COVID-19-related phenotypes: (1) individuals critically ill with COVID-19 on the basis of requiring respiratory support in hospital or who died as a consequence of the disease (9,376 cases, of which 3,197 are new in this data release, and 1,776,645 control individuals); (2) individuals with moderate or severe COVID-19 defined as those hospitalized due to symptoms associated with the infection (25,027 cases, 11,386 new and 2,836,272 control individuals); and (3) all cases with reported SARS-CoV-2 infection regardless of symptoms (125,584 cases, 76,022 new and 2,575,347 control individuals). Most studies have reported results before the roll out of the COVID-19 vaccination campaign. An overview of the study design is provided in Supplementary Fig. 1. We found a total of 23 genome-wide significant loci (*P* < 5 × 10^−8^) of which 20 loci remain significant after correction for multiple testing (*P* < 1.67 × 10^−8^) to account for the number of phenotypes examined (Fig. 2, Supplementary Fig. 2 and Supplementary Table 2). We compared the effects of these loci between the previous^2^ and current analysis and found that only one locus did not replicate (rs72711165). All of the other loci showed the expected increase in statistical significance (Supplementary Fig. 3).

**Fig. 1:**
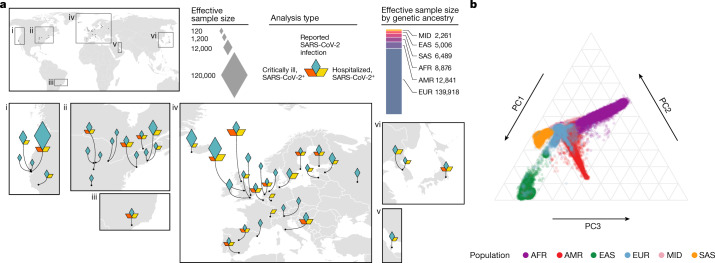
Overview of contributing studies in Host Genetics Initiative data freeze 6. **a**, Geographical overview of the contributing studies to the COVID-19 Host Genetics Initiative and composition by major continental ancestry groups. Ancestry groups are defined as Middle Eastern (MID), south Asian (SAS), east Asian (EAS), African (AFR), admixed American (AMR) and European (EUR). **b**, Principal components analysis highlighting the population structure and the sample ancestry of the individuals participating in the COVID-19 Host Genetics Initiative. This figure is reproduced from the original publication by the COVID-19 Host Genetics Initiative^2^ with modifications reflecting the updated analysis from data freeze 6.

**Fig. 2:**
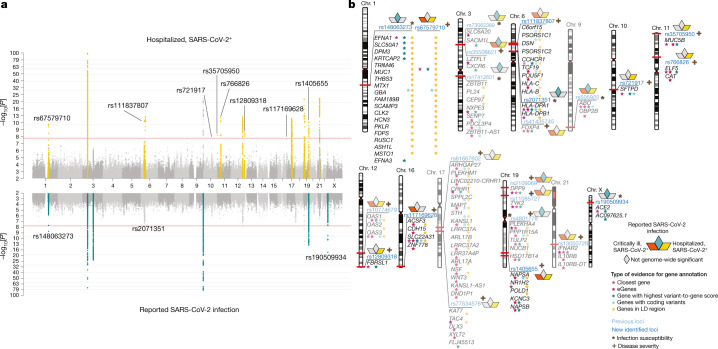
Genome-wide association results for COVID-19. **a**, The results of the genome-wide association study of hospitalized COVID-19 (*n* = 25,027 cases and *n* = 2,836,272 control individuals) (top), and the results of reported SARS-CoV-2 infection (*n* = 125,584 cases and *n* = 2,575,347 control individuals) (bottom). Loci highlighted in yellow (top) represent regions associated with the severity of COVID-19 manifestation. Loci highlighted in green (bottom) are regions associated with SARS-CoV-2-reported infection. Lead variants for the loci identified in this data release are annotated with their respective rs ID. Horizontal lines denote genome-wide significant thresholds. **b**, The results of gene prioritization using different evidence measures of gene annotation. Genes in regions of linkage disequilibrium (LD), genes with coding variants and eGenes (fine-mapped *cis*-eQTL variant PIP > 0.1 in GTEx Lung) are annotated if in linkage disequilibrium with a COVID-19 lead variant (*r*^2^ > 0.6). V2G denotes the highest gene prioritized by OpenTargetGenetics’ V2G score. The asterisk (*) indicates SARS-CoV-2 reported infection and the plus symbol (+) indicates COVID-19 severity. The transparent loci were reported in the previous freeze (data release 5), and loci in bright blue were identified in the current freeze (data release 6). This figure is reproduced from the original publication by the COVID-19 Host Genetics Initiative^2^ with modifications reflecting the updated analysis from data freeze 6.

Across the genome-wide significant loci, we observed clear patterns of association with the different phenotypes under study. We therefore developed a two-class Bayesian model for classifying loci based on the patterns of association across the two better-powered phenotypes (COVID-19 hospitalization and SARS-CoV-2 reported infection). Intuitively, loci that are associated with susceptibility will also be associated with severity as, to develop COVID-19, SARS-CoV-2 infection needs to first occur. By contrast, those genetic effects that solely modify the course of illness should be associated with severity of illness and not show any association with reported infection except through preferential ascertainment of hospitalized cases in a cohort (Supplementary Methods). We identified 16 loci that are substantially more likely (>99% posterior probability) to affect the risk of COVID-19 hospitalization and 7 loci that clearly influence susceptibility to SARS-CoV-2 infection (Supplementary Table 3 and Supplementary Fig. 4).

We observed that several loci had a significant heterogeneous effect across studies (6 out of 23 loci with a *P* value for heterogeneity of <2.2 × 10^−3^; Supplementary Table 2). Owing to an increased diversity in our study population (Supplementary Fig. 5), we were able to examine whether such heterogeneity was due to effect differences across continental ancestry groups. Only one locus (*FOXP4*) showed a significantly different effect across ancestries (*P* value heterogeneity of <7 × 10^−5^; Supplementary Table 4 and Supplementary Fig. 6), although even at this locus all of the ancestry groups showed a positive effect estimate. This confirms that factors related to between-study heterogeneity (such as variable definition of COVID-19 severity owing to different thresholds for testing, hospitalization and patient recruitment) rather than differences across ancestries are a more likely explanation for the observed heterogeneity in the effect sizes across studies.

For the 23 genome-wide significant loci, we examined candidate causal genes and performed a phenome-wide association study to better understand their potential biological mechanisms (Supplementary Tables 2, 5 and 6 and Supplementary Fig. 7). Several of these loci with previous and direct connections to lung disease and SARS-CoV-2 infection mechanisms are highlighted here.

Several loci involved in COVID-19 severity implicate lung surfactant biology. A missense variant rs721917:A>G (p.Met31Thr) in *SFTPD* (10q22.3) confers risk for hospitalization (odds ratio (OR) = 1.06, 95% confidence interval (CI) = 1.04–1.08, *P* = 1.7 × 10^–8^) and has been previously associated with increased risk of chronic obstructive pulmonary disease^3^ (OR = 1.08, *P* = 2.0 × 10^–8^) and decreased lung function^4^ (FEV1/FVC; *β* = –0.019; *P* = 2.0 × 10^–15^). *SFTPD* encodes surfactant protein D (SP-D), which participates in innate immune response, protecting the lungs against inhaled microorganisms. The recombinant fragment of SP-D binds to the S1 spike protein of SARS-CoV-2 and potentially inhibits binding to ACE2 receptor and SARS-CoV-2 infection^5^. Another missense variant rs117169628:G>A (p.Pro256Leu) in *SLC22A31* (16q24.3) also confers risk of hospitalization (OR = 1.09, 95% CI = 1.06–1.13, *P* = 2.6 × 10^–8^). *SLC22A31* belongs to the family of solute carrier proteins that facilitate transport across membranes^6^ and is co-regulated with other surfactant proteins^7^.

We found that the variant rs35705950:G>T located in the promoter of *MUC5B* (11p15.5) is protective against hospitalization (OR = 0.83, 95% CI = 0.86–0.93, *P* = 6.5 × 10^–9^). This well-studied promoter variant increases the expression of *MUC5B* in lung in GTEx (*P* = 6.7 × 10^–16^) and is the strongest known variant associated with an increased risk of developing idiopathic pulmonary fibrosis (IPF)^8,9^, but also improves survival in patients with IPF carrying this mutation^10^.

Finally, we found that rs190509934:T>C, which is located 69 bp upstream of *ACE2* (Xp22.2), is associated with decreased susceptibility risk (OR = 0.69, 95% CI = 0.63–0.75, *P* = 3.6 × 10^–18^). ACE2 is the SARS-CoV-2 receptor and functionally interacts with SLC6A19 and SLC6A20^11^, one of which also showed a significant association with susceptibility (rs73062389:G>A at *SLC6A20*; OR = 1.18, 95% CI = 1.16–1.20, *P* = 2.5 × 10^–74^). Notably, rs190509934 is ten times more common in south Asian populations (minor allele frequency (MAF) = 0.027) than in European populations (MAF = 0.0024), demonstrating the importance of diversity for variant discovery. Recent results have shown that the rs190509934:T>C variant lowers *ACE2* expression, which in turn confers protection against SARS-CoV-2 infection^12^.

We applied Mendelian randomization to infer potential causal relationships between COVID-19-related phenotypes and their genetically correlated traits (Supplementary Methods; Supplementary Tables 7–9 and Supplementary Fig. 8). A causal association was observed between genetic liability to type 2 diabetes and SARS-CoV-2 reported infection (OR = 1.02, 95% CI = 1.01–1.03, *P* = 1.6 × 10^−3^), and COVID-19 hospitalization (OR = 1.06, 95% CI = 1.03–1.1, *P* = 1.4 × 10^−4^). Multivariable Mendelian randomization was used to estimate the direct effect of liability to type 2 diabetes on COVID-19-related phenotypes that was not mediated through body mass index. This analysis indicated that the observed causal association of liability to type 2 diabetes on COVID-19 phenotypes is mediated by body mass index (Supplementary Table 10).

We have substantially expanded the genetic analysis of SARS-CoV-2 infection and COVID-19 severity by doubling the case size, identifying 11 loci. We developed an approach to systematically assign the 23 discovered loci to either disease susceptibility (7 loci) or disease severity (16 loci). Although distinguishing between the two phenotypes is challenging because progression to a severe form of the disease requires susceptibility to infection in the first place, it is now evident that the genetic mechanisms involved in these two aspects of the disease can be differentiated. Among the new loci associated with disease susceptibility, *ACE2* represents an expected, albeit interesting, finding. *MUC5B*, *SFTPD* and *SLC22A31* are the three most interesting new loci associated with COVID-19 severity. Their relationship with lung function and lung diseases is consistent with loci previously associated with disease severity. The surfactant proteins secreted by alveolar cells, representing an emerging biological mechanism, maintain healthy lung function and facilitate the clearance of pathogens^13^. The protective effect of the *MUC5B* variant is unexpected given the otherwise risk-increasing, concordant effect between IPF and COVID-19 observed for other variants^9^. Nonetheless, this result aligns with the *MUC5B* promoter variant association that shows a twofold higher survival rate among patients with IPF^10^. In mice, *Muc5b* seems to be essential for effective mucociliary clearance and for controlling infection^14^, which suggests that therapies to control mucin secretion may be beneficial in patients with COVID-19.

Expanding genomic research to include participants from around the world enabled us to test whether the effect of COVID-19-related genetic variants was markedly different across ancestry groups. We did not detect obvious heterogeneity between ancestry groups, and we attribute the observed heterogeneity in the effect of COVID-19-related genetic variants to the diverse inclusion criteria across studies in terms of COVID-19 severity. However, we also note that ascertainment differences across studies might mask true underlying differences in effect sizes between ancestry groups.

The biological insights gained by this expansion of the COVID-19 Host Genetic Initiative showed that increasing sample size and diversity remain a fruitful activity to better understand the human genetic architecture of COVID-19.

## Reporting summary

Further information on research design is available in the Nature Research Reporting Summary linked to this paper.

## Online content

Any methods, additional references, Nature Research reporting summaries, source data, extended data, supplementary information, acknowledgements, peer review information; details of author contributions and competing interests; and statements of data and code availability are available at 10.1038/s41586-022-04826-7.

## Supplementary information

Supplementary Information Supplementary Methods with additional references, and Supplementary Figs. 1–8.

Reporting Summary

Supplementary Tables 1–11

## Data Availability

Summary statistics generated by COVID-19 Host Genetics Initiative are available online (https://www.covid19hg.org/results/r6/). The analyses described here use the freeze 6 data. The COVID-19 Host Genetics Initiative continues to regularly release new data freezes. Summary statistics for samples from individuals of non-European ancestry are not currently available owing to the small individual sample sizes of these groups, but the results for 23 loci lead variants are reported in Supplementary Table 3. Individual-level data can be requested directly from the authors of the contributing studies, listed in Supplementary Table 1. We used publicly available data from GTEx (https://gtexportal.org/home/), the Neale laboratory (http://www.nealelab.is/uk-biobank/), the Finucane laboratory (https://www.finucanelab.org), the FinnGen Freeze 4 cohort (https://www.finngen.fi/en/access_results) and eQTL catalogue release 3 (http://www.ebi.ac.uk/eqtl/).

## Code availability

The code for summary statistics lift-over, the projection PCA pipeline including precomputed loadings and meta-analyses are available on GitHub (https://github.com/covid19-hg/), and the code for the Mendelian randomization and genetic correlation pipeline is available at GitHub (https://github.com/marcoralab/MRcovid). Codes for implementing the multivariable Mendelian randomization analysis and subtype analyses are available at GitHub (https://github.com/marcoralab/multivariate_MR and https://github.com/mjpirinen/covid19-hgi_subtypes).

## References

[1] The COVID-19 Host Genetics Initiative. The COVID-19 Host Genetics Initiative, a global initiative to elucidate the role of host genetic factors in susceptibility and severity of the SARS-CoV-2 virus pandemic. *Eu. J. Hum. Genet.* **28**, 715–718 (2020). 10.1038/s41431-020-0636-6 PMC7220587 32404885

[2] COVID-19 Host Genetics Initiative. Mapping the human genetic architecture of COVID-19. *Nature* **600**, 472–477 (2021). 10.1038/s41586-021-03767-x PMC8674144 34237774

[3] Hobbs, B. D. et al. Genetic loci associated with chronic obstructive pulmonary disease overlap with loci for lung function and pulmonary fibrosis. *Nat. Genet.* **49**, 426–432 (2017). 28166215 10.1038/ng.3752 PMC5381275

[4] Shrine, N. et al. New genetic signals for lung function highlight pathways and chronic obstructive pulmonary disease associations across multiple ancestries. *Nat. Genet.* **51**, 481–493 (2019). 30804560 10.1038/s41588-018-0321-7 PMC6397078

[5] Hsieh, M.-H. et al. Human surfactant protein D binds spike protein and acts as an entry inhibitor of SARS-CoV-2 pseudotyped viral particles. *Front. Immunol.* **12**, 641360 (2021). 34054808 10.3389/fimmu.2021.641360 PMC8161545

[6] Hediger, M. A. et al. The ABCs of solute carriers: physiological, pathological and therapeutic implications of human membrane transport proteins. *Pflugers Arch.* **447**, 465–468 (2004). 14624363 10.1007/s00424-003-1192-y

[7] Deelen, P. et al. Improving the diagnostic yield of exome-sequencing by predicting gene-phenotype associations using large-scale gene expression analysis. *Nat. Commun.* **10**, 2837 (2019). 31253775 10.1038/s41467-019-10649-4 PMC6599066

[8] Seibold, M. A. et al. A common MUC5B promoter polymorphism and pulmonary fibrosis. *N. Engl. J. Med.* **364**, 1503–1512 (2011). 21506741 10.1056/NEJMoa1013660 PMC3379886

[9] Fadista, J. et al. Shared genetic etiology between idiopathic pulmonary fibrosis and COVID-19 severity. *EBioMedicine* **65**, 103277 (2021). 33714028 10.1016/j.ebiom.2021.103277 PMC7946355

[10] Peljto, A. L. et al. Association between the MUC5B promoter polymorphism and survival in patients with idiopathic pulmonary fibrosis. *JAMA* **309**, 2232–2239 (2013). 23695349 10.1001/jama.2013.5827 PMC4545271

[11] Vuille-Dit-Bille, R. N. et al. Human intestine luminal ACE2 and amino acid transporter expression increased by ACE-inhibitors. *Amino Acids* **47**, 693–705 (2014). 25534429 10.1007/s00726-014-1889-6

[12] Horowitz, J. E. et al. Common genetic variants identify targets for COVID-19 and individuals at high risk of severe disease. Preprint at *medRxiv*10.1101/2020.12.14.20248176 (2021).

[13] Wright, J. R. Immunoregulatory functions of surfactant proteins. *Nat. Rev. Immunol.* **5**, 58–68 (2005). 15630429 10.1038/nri1528

[14] Roy, M. G. et al. Muc5b is required for airway defence. *Nature* **505**, 412–416 (2014). 24317696 10.1038/nature12807 PMC4001806

