# Supplementary material for: A first update on mapping the human genetic architecture of COVID-19
Source: Nature. 2022 Aug 3;608(7921):E1–E10. doi: 10.1038/s41586-022-04826-7 (PMC9352569; doi:10.1038/s41586-022-04826-7)

nature portfolio

https://doi.org/10.1038/s41586-022-04826-7

Supplementary information

A first update on mapping the human genetic architecture of COVID-19

In the format provided by the authors and unedited

METHODS

Ethical statements for each contributing study are given in Supplementary Table 1.

Probabilistic assignment of variants into susceptibility vs. severity effects

We consider an assignment of variants reported in **Supplementary Table 3** into two groups based on whether they seem to affect susceptibility to corona virus infection (INF) or severity of the infection (SEV). For this analysis, we meta-analyzed GWAS summary statistics from such versions of B2 (hospitalized for COVID vs. population) and C2 (infected vs. population) GWAS that only included those studies that had contributed some data to B2. Therefore, all studies included in this analysis had made some effort to distinguish from all the infected individuals those individuals who additionally were hospitalized. The sample sizes of these GWAS were 23,988 cases / 2,834,885 controls for B2 and 114,516 cases / 2,138,237 controls for C2. This corresponds to an effective samples size of 84,292 for the hospitalized analysis and 404,416 for the reported infection analysis.

We assume that all hospitalized cases (B2 cases) were included among the infected cases (C2 cases) of the corresponding study and that, for each study, the controls of B2 and C2 had a maximum overlap possible given the control counts in the two data sets.

Next, we explain how we defined the statistical models that represent INF and SEV, and how we compared these models at each SNP.

Intuitively, INF represents a variant that associates with susceptibility of infection but has no effect on the severity of infection. The allele frequency of such a variant is similar among the hospitalized cases (B2 cases) as it is among all infected (C2 cases). Thus, under the INF model, we assume similar effect size between C2 and B2, i.e., $\beta_{C2} \approx \beta_{B2}$.

Model SEV represents a variant that affects severity of infection ($|\beta_{B2}| > 0$) but not susceptibility to infection. If the cases of our susceptibility scan C2 were a random subset of infected individuals, then under the SEV model, $\beta_{C2} \approx 0$. However, since our C2 cases are strongly enriched for severe cases, we expect that in our data also $|\beta_{C2}| > 0$ even when a variant is affecting severity of infection but not susceptibility to infection. We expect that, for such variants, the effect size in C2 is proportional to its effect in B2, i.e., $\beta_{C2} \approx w_{SEV}\beta_{B2}$, where the constant of proportionality, $w_{SEV} < 1$, depends on the proportion of all C2 cases that are also B2 cases. If we imagine C2 analysis as a fixed-effect meta-analysis between B2 and an (imaginary) "non-severe infection vs. population" analysis that had no sample overlap with B2 analysis, then $w_{SEV} = n_{B2}^{(eff)}/n_{C2}^{(eff)}$, where $n_i^{(eff)} = R_i S_i / N_i$ is the effective sample size of study *i* with R_i the number of controls, S_i the number of cases and $N_i = R_i + S_i$. In our data, $n_{B2}^{(eff)}/n_{C2}^{(eff)} \approx 0.208$, where the effective sample sizes are computed by summing the effective sample sizes over individual studies of B2 and C2 analyses. Even when controls of B2 analysis and the imaginary "non-severe infection vs. population" analysis overlapped completely, the value of w_{SEV} would change

little in these data. After accounting for the overlap in controls, we estimated a value of w_{SEV} = 0.20 that we used in the analyses described below.

To derive correlation r_{B2C2} between the effect size estimators of B2 and C2 analyses due to overlapping samples, we used formula

$$r_{B2C2} = \frac{\sum_{k=1}^{K} \sqrt{n_{B2,k}^{(eff)}\, n_{C2,k}^{(eff)}}\, r_{B2C2,k}}{\sqrt{\sum_{k=1}^{K} n_{B2,k}^{(eff)}\, \sum_{k=1}^{K} n_{C2,k}^{(eff)}}}$$

where subscript k refers to individual studies. The correlation $r_{B2C2,k}$ between B2 and C2 for study k is computed as in Bhattacharjee et al.[1]:

$$r_{B2C2,k} = \sqrt{n_{B2,k}^{(eff)}\, n_{C2,k}^{(eff)}} \left(\frac{S_{B2C2,k}}{S_{B2,k} S_{C2,k}} + \frac{R_{B2C2,k}}{R_{B2,k} R_{C2,k}} \right)$$

where $S_{B2C2,k}$ is the number of shared B2 and C2 cases in study k and similarly $R_{B2C2,k}$ is the number of shared controls. By applying this to the data, we estimated r_{B2C2} = 0.45.

With these estimates of w_{SEV} = 0.2 and r_{B2C2} = 0.45, and with the observed data at any one SNP containing effect estimates $(\hat{\beta}_{B2}, \hat{\beta}_{C2})$ and their standard errors (s_{B2}, s_{C2}), we derive the two models, INF and SEV, as follows.

Prior distribution for effects is zero-centered bivariate normal distribution

$$\begin{pmatrix} \beta_{B2} \\ \beta_{C2} \end{pmatrix} \sim N\left(\begin{pmatrix} 0 \\ 0 \end{pmatrix}, \Theta_i \right), \text{ with}$$

$$\Theta_{INF} = \tau^2 \begin{pmatrix} 1 & 1 - \eta_{INF} \\ 1 - \eta_{INF} & 1 \end{pmatrix} \text{ and}$$

$$\Theta_{SEV} = \tau^2 \begin{pmatrix} 1 & (1 - \eta_{SEV}) w_{SEV} \\ (1 - \eta_{SEV}) w_{SEV} & w_{SEV}^2 \end{pmatrix}.$$

We have used value $\tau = 0.1$ to define the expected effect sizes of the B2 analysis (implying roughly that 95% of the true effect sizes of the risk variants have odds-ratio below 1.2). By tuning the parameters η_{INF} and η_{SEV}, we can define how much deviation real effects can have from the theoretical relationships $\beta_{C2} = \beta_{B2}$ and $\beta_{C2} = w_{SEV}\beta_{B2}$ corresponding to models INF and SEV, respectively. We have set these values in such a way that, under both models, the mean (Euclidean) distance between the effect size and the corresponding line is 0.0025 and 95% of the effects are within 0.006 units from the line. This happens when $\eta_{INF} = 0.001$ and $\eta_{SEV} = 0.013$.

The likelihood for the observed data under both models is Gaussian

$$\begin{pmatrix}\hat{\beta}_{B2}\\ \hat{\beta}_{C2}\end{pmatrix} \sim N\left(\begin{pmatrix}\beta_{B2}\\ \beta_{C2}\end{pmatrix}, \Sigma\right), \text{where } \Sigma = \begin{pmatrix} s_{B2}^2 & s_{B2}s_{C2}r_{B2C2} \\ s_{B2}s_{C2}r_{B2C2} & s_{C2}^2\end{pmatrix}.$$

It follows from Trochet et al.[2] that we can analytically integrate the likelihood with respect to the prior distributions and the resulting marginal likelihood for each model is proportional to a Gaussian density function evaluated at the observed effect size estimates as

$$\Pr(\text{DATA} \mid \text{Model } i) \propto f_N\left(\begin{pmatrix}\hat{\beta}_{B2}\\ \hat{\beta}_{C2}\end{pmatrix};\ \begin{pmatrix}0\\0\end{pmatrix}, \Theta_i + \Sigma\right).$$

We set equal prior probability on each model (i.e. 50% on INF and 50% on SEV), and consequently the posterior probabilities of the models will be proportional to their marginal likelihoods. These posterior probabilities are reported in Supplementary Table 3.

A limitation of this approach is that it classifies every variant between the two fixed models INF and SEV without considering a possibility that the variant might not fit either of these two models very well. We have chosen this approach since, based on the data shown in **Supplementary Figure 4**, a large majority of the variants are well aligned with either INF or SEV model. Consequently, we kept this model comparison simple and only between INF and SEV rather than complicated it by inclusion of some additional models that would have little support from the observed data.

Gene prioritization and Phenome wide association study (PheWAS)
The variant annotation and PheWAS were performed as per methods described in Niemi et. al 2021[3].

Genetic Correlations and Mendelian Randomization
The genetic correlation, heritability estimates, and Mendelian Randomization were performed as per methods described in Niemi et. al 2021[3].

SNP heritability for all three COVID-19 related phenotypes was low (<1% on the observed scale; Supplementary Table 7). To understand which traits are genetically correlated and/or potentially causally related to the three phenotypes, we first estimated genetic correlations with 38 traits (Supplementary Table 8). In addition to what was previously reported, we found positive genetic correlations with two risk factors (depression and insomnia symptoms), two biomarkers (C-reactive protein (CRP) and 25-hydroxyvitamin D), and two disease liability traits (asthma and heart failure).

We next applied two-sample Mendelian Randomization (MR) to infer potential causal relationships between COVID-19 related phenotypes and their genetically correlated traits. Four traits (BMI, type II diabetes, red blood cell count, and height) showed evidence of causal

associations after correcting for multiple testing and were robust to potential violations of the underlying assumptions of MR (Supplementary Table 9).

Multivariable Mendelian Randomization (MVMR) was used to estimate the direct effects of body mass index (BMI) and type II diabetes (T2D) on the risk of SARS-CoV-2 phenotypes, by including both exposures within the same model. We selected all independent ($r^2 = 0.001$; kb = 10000) genome-wide significant ($p < 5x10^{-8}$) SNPs associated with BMI and type II diabetes; and using the full list of SNPs associated with both BMI and T2D, performed a second clumping procedure ($r^2 = 0.001$; kb = 10000) to obtain independent SNPs. After clumping the full list of SNPs from both BMI and T2D and restricting to SNPs found in the SARS-CoV-2 GWASs, a total of 721 SNPs were available for multivariable MR (516 were associated with BMI only, 201 were associated with T2D only, and 4 SNPs overlap between both GWAS). MVMR was performed using the “MVMR” package [1]. The sensitivity analyses conducted using “MVMR” require estimates of the pairwise covariances between each instrument and each exposure, as such, we used the phenotypic correlation between BMI and T2D and summary data to generate estimates of the covariances. Phenotypic correlations between BMI and T2D were estimated from the LDSC regression intercept ($r^2 = 0.13$) [4]. Next, we calculated conditional F-statistics to evaluate the presence of weak instruments. The conditional F-statistic for both BMI and T2D were >10, indicating that the selected instruments were strongly associated with their corresponding exposure (Supplementary Table 10b) [5,6]. We then assessed multivariable instrument pleiotropy using the modified Cochran’s Q-statistic that accounts for potential weak instrument bias, with evidence of heterogeneity indicative of a violation in the escalation restriction assumption in MR [5,6] . For each of the SARS-CoV-2 phenotypes MVMR models there was evidence of heterogeneity, suggesting the causal estimates from IVW-MVMR may be biased due to the presence of horizontal pleiotropy (Supplementary Table 10c). Inverse weighted multivariable MR model was used to estimate the direct effect of BMI and T2D upon each of the SARS-CoV-2 phenotypes (Supplementary Table 10). When MVMR assumptions are violated, as indicated by the presence of weak instruments or horizontal pleiotropy, it is possible to obtain more robust causal estimates Q- statistic to minimization (Supplementary Table 10).

Code for implementing the MVMR analysis is available at:
https://github.com/marcoralab/multivariate_MR

References

1. Bhattacharjee, S. *et al.* A subset-based approach improves power and interpretation for the combined analysis of genetic association studies of heterogeneous traits. *Am. J. Hum. Genet.* **90,** 821–835 (2012).
2. Trochet, H. *et al.* Bayesian meta-analysis across genome-wide association studies of diverse phenotypes. *Genet. Epidemiol.* **43,** 532–547 (2019).
3. COVID-19 Host Genetics Initiative. Mapping the human genetic architecture of COVID-19. *Nature* (2021). doi:10.1038/s41586-021-03767-x

4. Zheng, J. *et al.* PhenoSpD: an integrated toolkit for phenotypic correlation estimation and multiple testing correction using GWAS summary statistics. *BioRxiv* (2017). doi:10.1101/148627
5. Sanderson, E., Davey Smith, G., Windmeijer, F. & Bowden, J. An examination of multivariable Mendelian randomization in the single-sample and two-sample summary data settings. *Int. J. Epidemiol.* **48,** 713–727 (2019).
6. Sanderson, E., Spiller, W. & Bowden, J. Testing and correcting for weak and pleiotropic instruments in two-sample multivariable Mendelian randomization. *Stat. Med.* **40,** 5434–5452 (2021).

SUPPLEMENTARY FIGURES

Supplementary Figure 1

Analytical summary of the COVID-19 HGI meta-analysis. Using the analytical plan set by the COVID-19 HGI, each individual study runs their analyses and uploads the results to the Initiative, who then runs the meta-analysis. There are three main analyses that each study can contribute summary statistics to: critically ill COVID-19, hospitalized COVID-19 and reported SARS-CoV-2 infection. The phenotypic criteria used to define cases are listed in the dark grey boxes, along with the numbers of cases (N) included in the final all ancestries meta-analysis. Controls were defined in the same way across all three analyses: as everybody that is not a case e.g. population controls (light grey box). Sensitivity analyses, not reported in this Figure, also used mild/asymptomatic COVID-19 cases as controls. Sample number (N) of controls differed between the analyses due to the difference in number of studies contributing data to these.

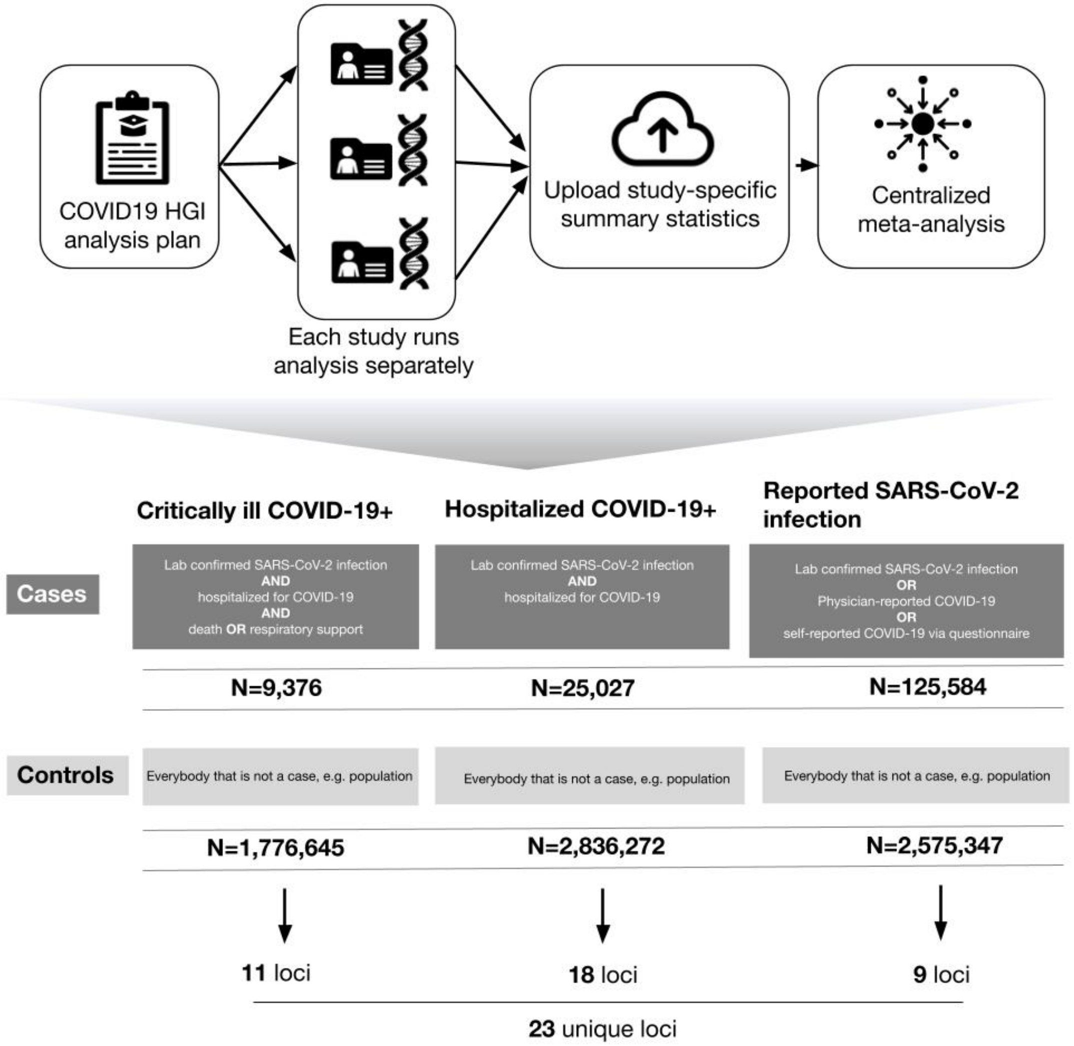

Supplementary Figure 2

Genome-wide association results for COVID-19 critical illness (Release 6). Results of genome-wide association study of critically ill COVID-19 cases vs population controls (n=9,376 cases and n=1,776,645 controls). Critically ill COVID-19 cases defined as those who required respiratory support in hospital or who were deceased due to the disease.

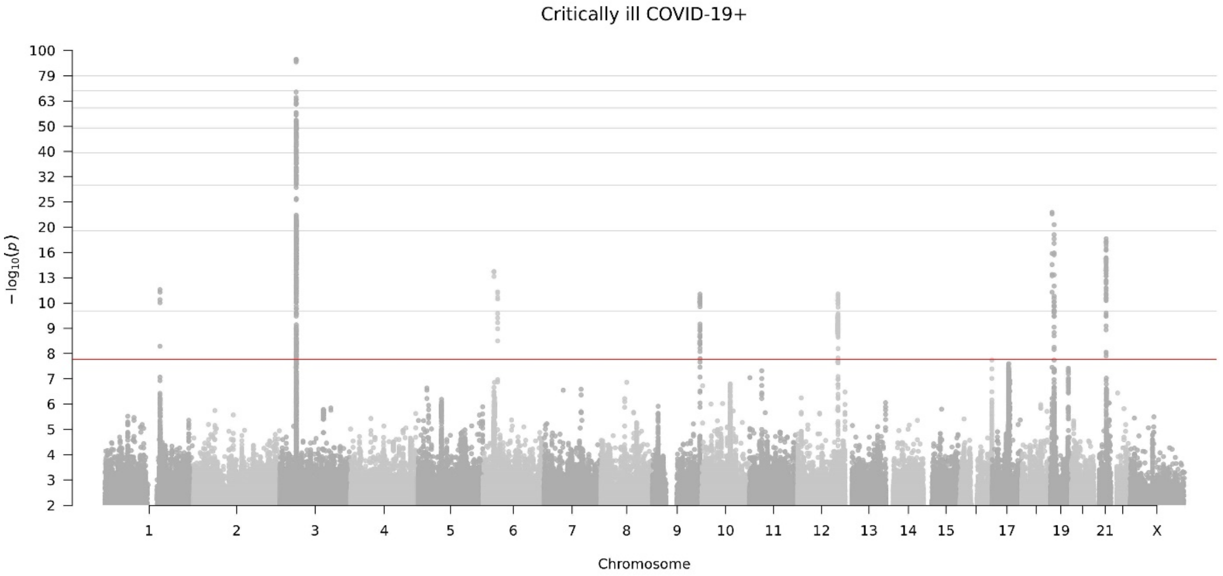

Supplementary Figure 3

Comparison of associations between release 5 and release 6. a. $\chi2$ statistics (y-axis) and effective sample size (x-axis) in release 5 (dots labelled as R5) and release 6 (dots labelled as R6), for each variant reported in release 6 and rs72711165. b. slope of the line represented in panel a, that is, the change in $\chi2$ statistics for each additional unit in the effective sample size, for each variant reported in release 6 and rs72711165. c. effect size absolute value in release 5 (x-axis) and release 6 (y-axis) and standard error, for each variant reported in release 6 and rs72711165.

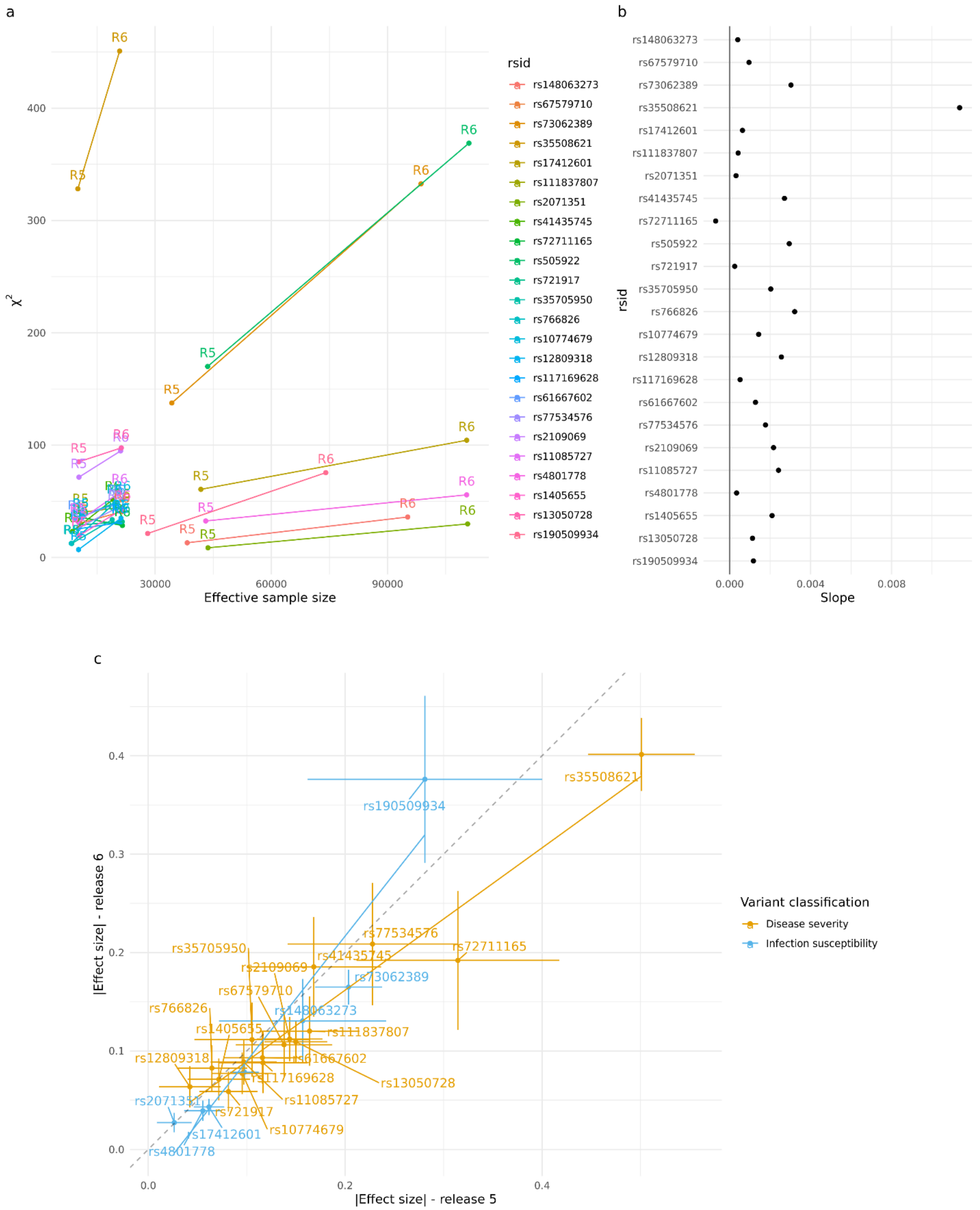
a
χ2
Effective sample size
rsid
rs148063273
rs67579710
rs73062389
rs35508621
rs17412601
rs111837807
rs2071351
rs41435745
rs72711165
rs505922
rs721917
rs35705950
rs766826
rs10774679
rs12809318
rs117169628
rs61667602
rs77534576
rs2109069
rs11085727
rs4801778
rs1405655
rs13050728
rs190509934
b
rsid
Slope
c
|Effect size| - release 6
|Effect size| - release 5
Variant classification
Disease severity
Infection susceptibility

Supplementary Figure 4

The estimated log odds ratios for 23 lead variants shown for B2 GWAS (hospitalized vs. controls) on x-axis and C2 GWAS (infected vs. controls) on y-axis. The assignment to variants affecting susceptibility to infection (INF) or disease severity (SEV) is shown by colors and corresponds to Supplementary Table 3. The two lines show the expected relationship between effect sizes of variants affecting susceptibility to infection (line y = x) or disease severity (line y = 0.2 x). In left panel, the variants have been annotated by rsids, and in right panel, 95% confidence intervals are shown.

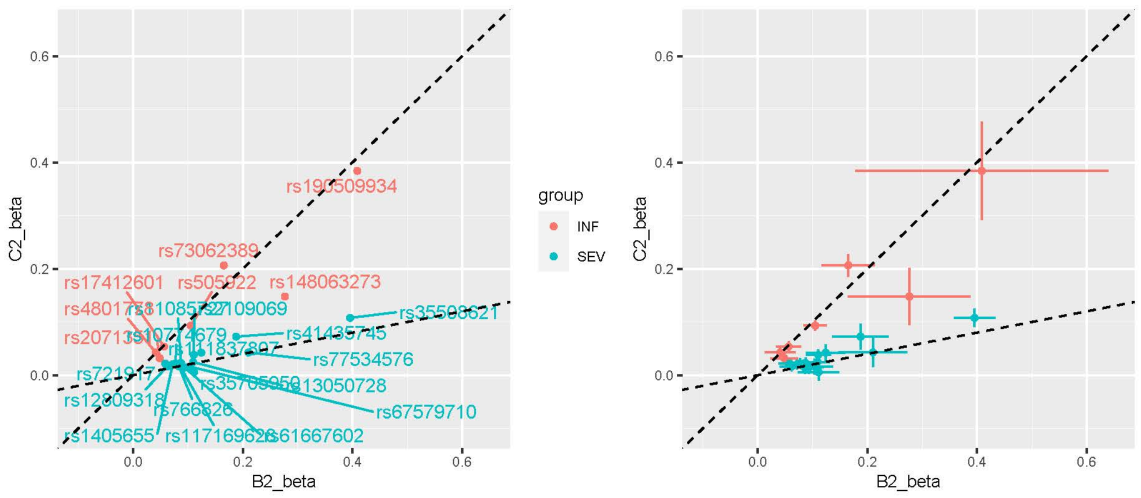

Supplementary Figure 5

Projection of contributed samples from participating studies into the same PC space. We asked participating studies to perform PC projection using the 1000 Genomes Project and Human Genome Diversity Project as a reference, with a common set of variants. For each panel (except for the reference), colored points correspond to contributed samples from each cohort, whereas gray points correspond to the 1000 Genomes reference samples. Color represents a genetic population that each cohort specified. Since 23andme, genomicsengland100kgp, and MVP only submitted PCA images, we overlaid their submitted transparent images using the same coordinates, instead of directly plotting them.

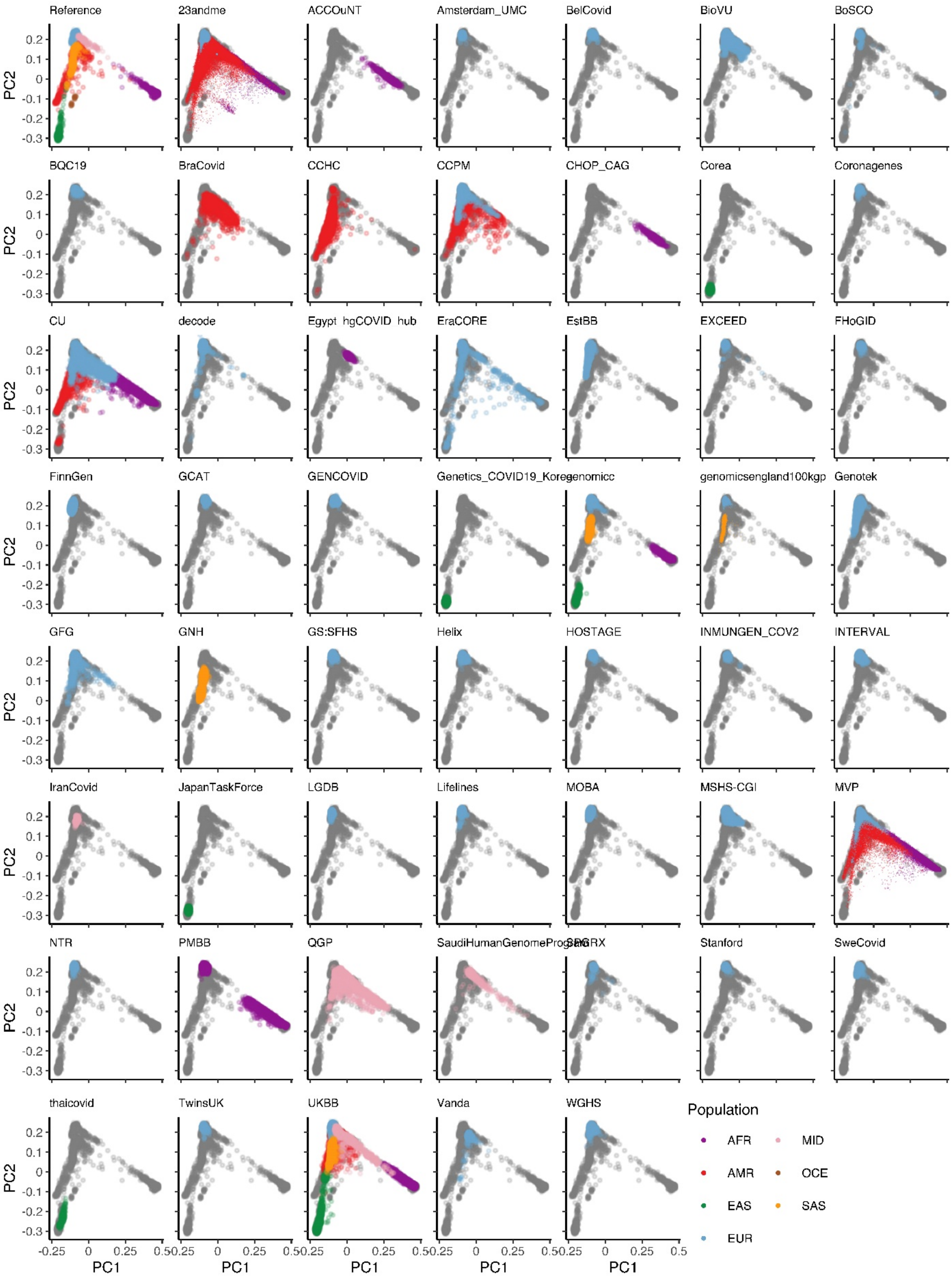
Reference
23andme
ACCOuNT
Amsterdam_UMC
BelCovid
BioVU
BoSCO
BQC19
BraCovid
CCHC
CCPM
CHOP_CAG
Corea
Coronagenes
CU
decode
Egypt_hgCOVID_hub
EraCORE
EstBB
EXCEED
FHoGID
FinnGen
GCAT
GENCOVID
Genetics_COVID19_Kore
genomicc
genomicsengland100kgp
Genotek
GFG
GNH
GS:SFHS
Helix
HOSTAGE
INMUNGEN_COV2
INTERVAL
IranCovid
JapanTaskForce
LGDB
Lifelines
MOBA
MSHS-CGI
MVP
NTR
PMBB
QGP
SaudiHumanGenomeProgram
SPGRX
Stanford
SweCovid
thaicovid
TwinsUK
UKBB
Vanda
WGHS
PC2
PC1
0.2
0.1
0
-0.1
-0.2
-0.3
-0.25
0
0.25
0.5
Population
AFR
AMR
EAS
EUR
MID
OCE
SAS

Supplementary Figure 6

Forest plots for highly heterogeneous loci. Forest plots for 6/23 loci that showed a significant heterogeneous effect across studies. For each of the loci, effect sizes and confidence intervals are reported for each contributing study. Studies are grouped by ancestry, with summary effects reported for each ancestry subgroup. The summary effect size across all studies is also reported in the bottom panel for each locus.

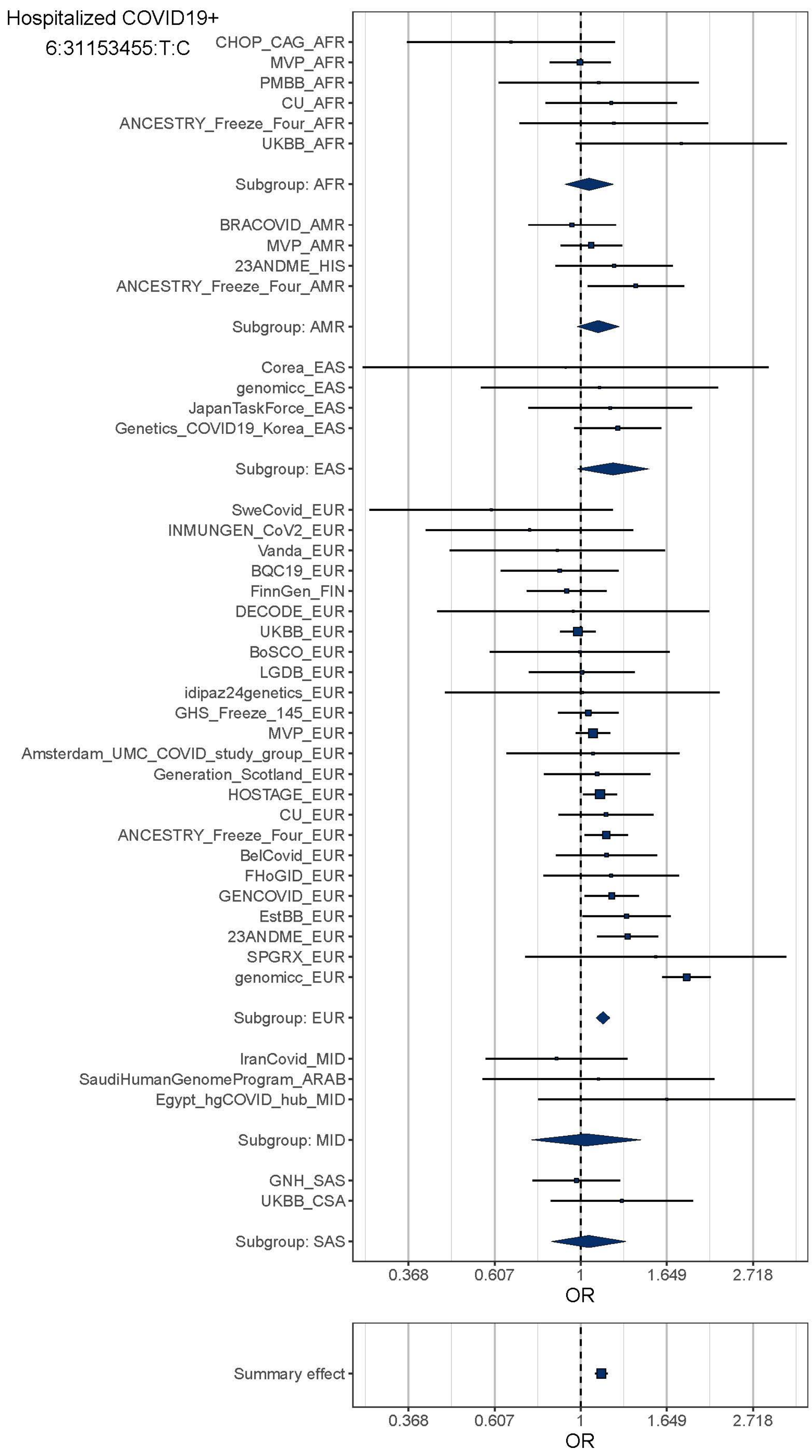
Hospitalized COVID19+
6:31153455:T:C
CHOP_CAG_AFR
MVP_AFR
PMBB_AFR
CU_AFR
ANCESTRY_Freeze_Four_AFR
UKBB_AFR
Subgroup: AFR
BRACOVID_AMR
MVP_AMR
23ANDME_HIS
ANCESTRY_Freeze_Four_AMR
Subgroup: AMR
Corea_EAS
genomicc_EAS
JapanTaskForce_EAS
Genetics_COVID19_Korea_EAS
Subgroup: EAS
SweCovid_EUR
INMUNGEN_CoV2_EUR
Vanda_EUR
BQC19_EUR
FinnGen_FIN
DECODE_EUR
UKBB_EUR
BoSCO_EUR
LGDB_EUR
idipaz24genetics_EUR
GHS_Freeze_145_EUR
MVP_EUR
Amsterdam_UMC_COVID_study_group_EUR
Generation_Scotland_EUR
HOSTAGE_EUR
CU_EUR
ANCESTRY_Freeze_Four_EUR
BelCovid_EUR
FHoGID_EUR
GENCOVID_EUR
EstBB_EUR
23ANDME_EUR
SPGRX_EUR
genomicc_EUR
Subgroup: EUR
IranCovid_MID
SaudiHumanGenomeProgram_ARAB
Egypt_hgCOVID_hub_MID
Subgroup: MID
GNH_SAS
UKBB_CSA
Subgroup: SAS
0.368
0.607
1
1.649
2.718
OR
Summary effect
0.368
0.607
1
1.649
2.718
OR

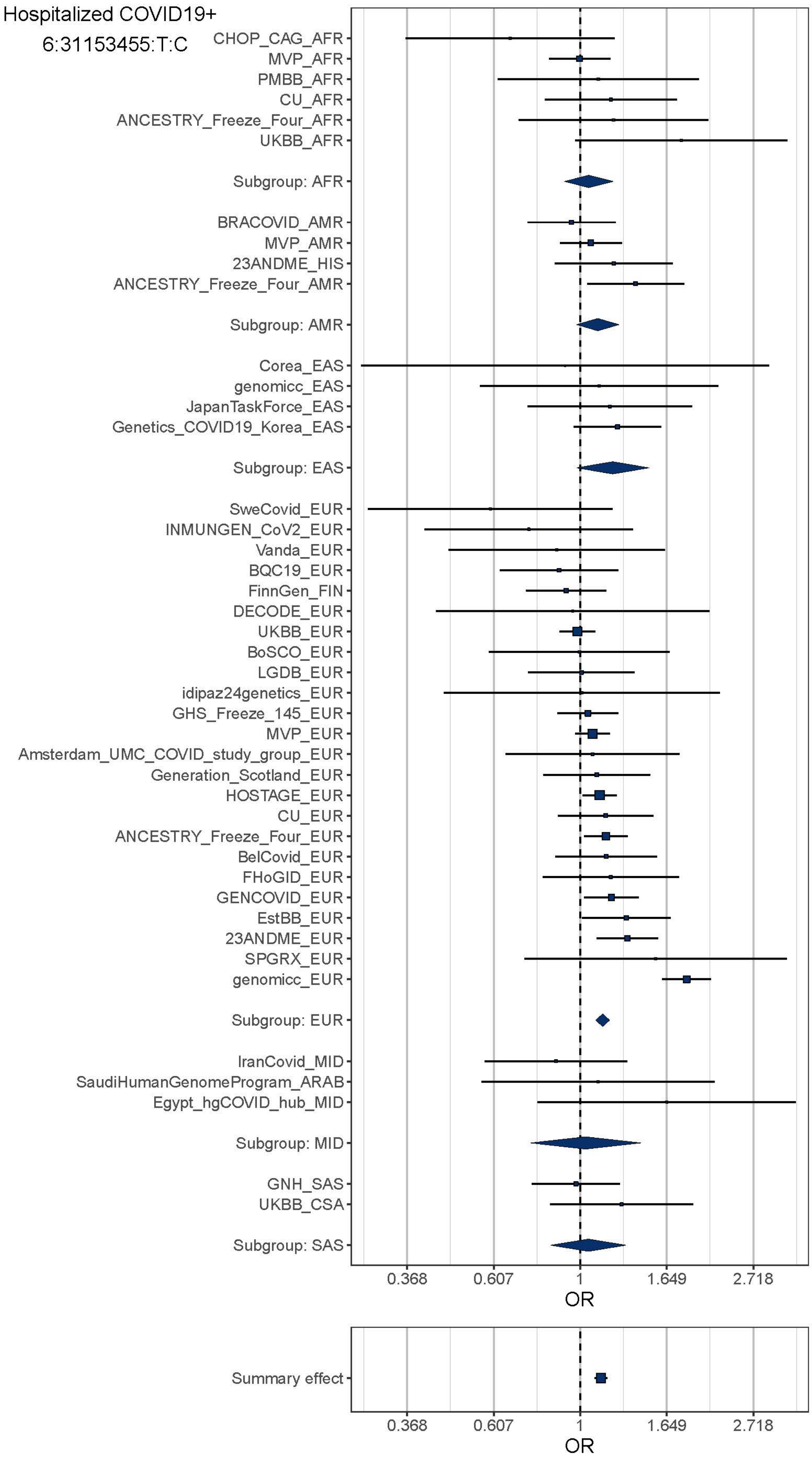
Hospitalized COVID19+
6:31153455:T:C
CHOP_CAG_AFR
MVP_AFR
PMBB_AFR
CU_AFR
ANCESTRY_Freeze_Four_AFR
UKBB_AFR
Subgroup: AFR
BRACOVID_AMR
MVP_AMR
23ANDME_HIS
ANCESTRY_Freeze_Four_AMR
Subgroup: AMR
Corea_EAS
genomicc_EAS
JapanTaskForce_EAS
Genetics_COVID19_Korea_EAS
Subgroup: EAS
SweCovid_EUR
INMUNGEN_CoV2_EUR
Vanda_EUR
BQC19_EUR
FinnGen_FIN
DECODE_EUR
UKBB_EUR
BoSCO_EUR
LGDB_EUR
idipaz24genetics_EUR
GHS_Freeze_145_EUR
MVP_EUR
Amsterdam_UMC_COVID_study_group_EUR
Generation_Scotland_EUR
HOSTAGE_EUR
CU_EUR
ANCESTRY_Freeze_Four_EUR
BelCovid_EUR
FHoGID_EUR
GENCOVID_EUR
EstBB_EUR
23ANDME_EUR
SPGRX_EUR
genomicc_EUR
Subgroup: EUR
IranCovid_MID
SaudiHumanGenomeProgram_ARAB
Egypt_hgCOVID_hub_MID
Subgroup: MID
GNH_SAS
UKBB_CSA
Subgroup: SAS
0.368
0.607
1
1.649
2.718
OR
Summary effect
0.368
0.607
1
1.649
2.718
OR

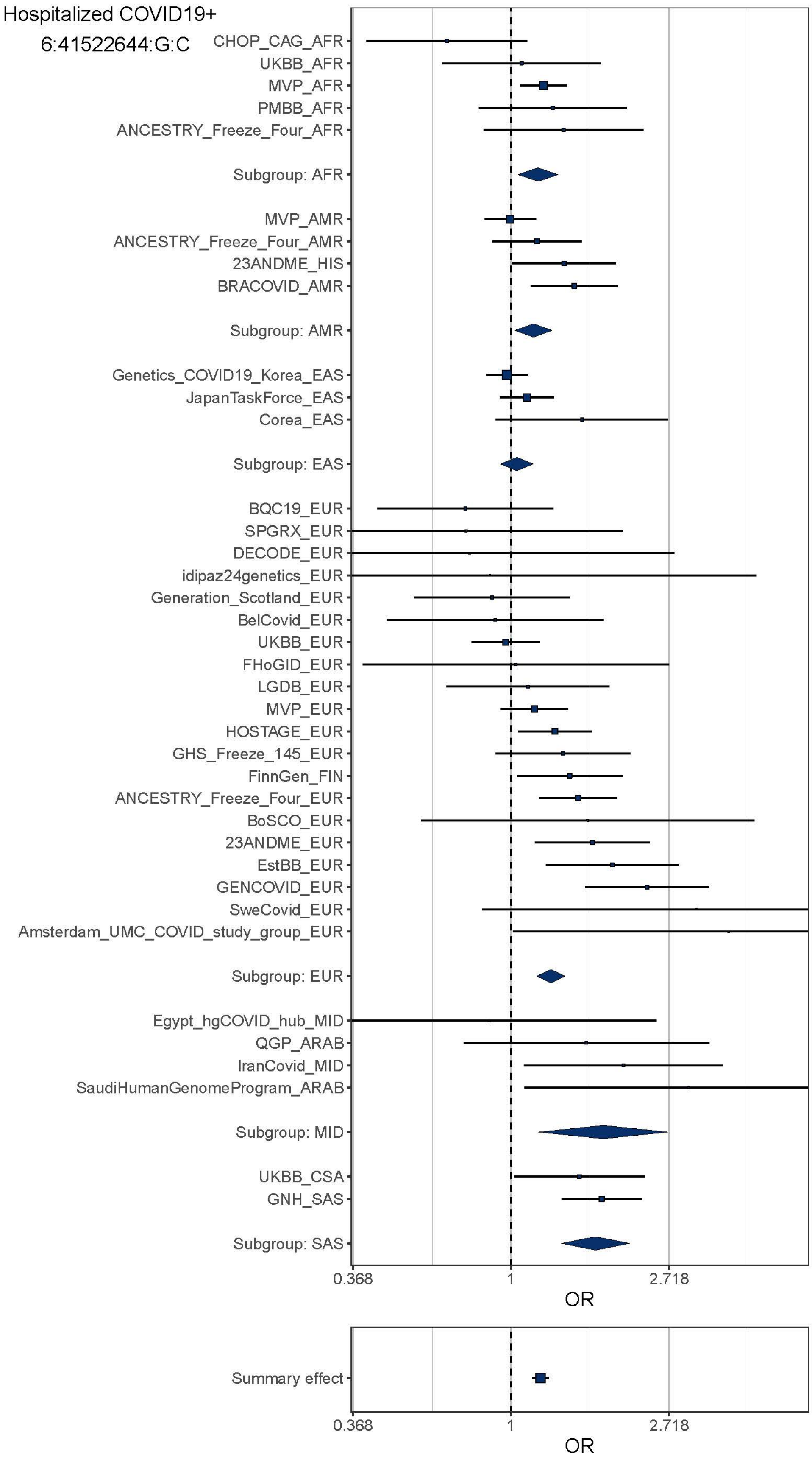
Hospitalized COVID19+
6:41522644:G:C
CHOP_CAG_AFR
UKBB_AFR
MVP_AFR
PMBB_AFR
ANCESTRY_Freeze_Four_AFR
Subgroup: AFR
MVP_AMR
ANCESTRY_Freeze_Four_AMR
23ANDME_HIS
BRACOVID_AMR
Subgroup: AMR
Genetics_COVID19_Korea_EAS
JapanTaskForce_EAS
Corea_EAS
Subgroup: EAS
BQC19_EUR
SPGRX_EUR
DECODE_EUR
idipaz24genetics_EUR
Generation_Scotland_EUR
BelCovid_EUR
UKBB_EUR
FHoGID_EUR
LGDB_EUR
MVP_EUR
HOSTAGE_EUR
GHS_Freeze_145_EUR
FinnGen_FIN
ANCESTRY_Freeze_Four_EUR
BoSCO_EUR
23ANDME_EUR
EstBB_EUR
GENCOVID_EUR
SweCovid_EUR
Amsterdam_UMC_COVID_study_group_EUR
Subgroup: EUR
Egypt_hgCOVID_hub_MID
QGP_ARAB
IranCovid_MID
SaudiHumanGenomeProgram_ARAB
Subgroup: MID
UKBB_CSA
GNH_SAS
Subgroup: SAS
0.368
1
2.718
OR
Summary effect
0.368
1
2.718
OR

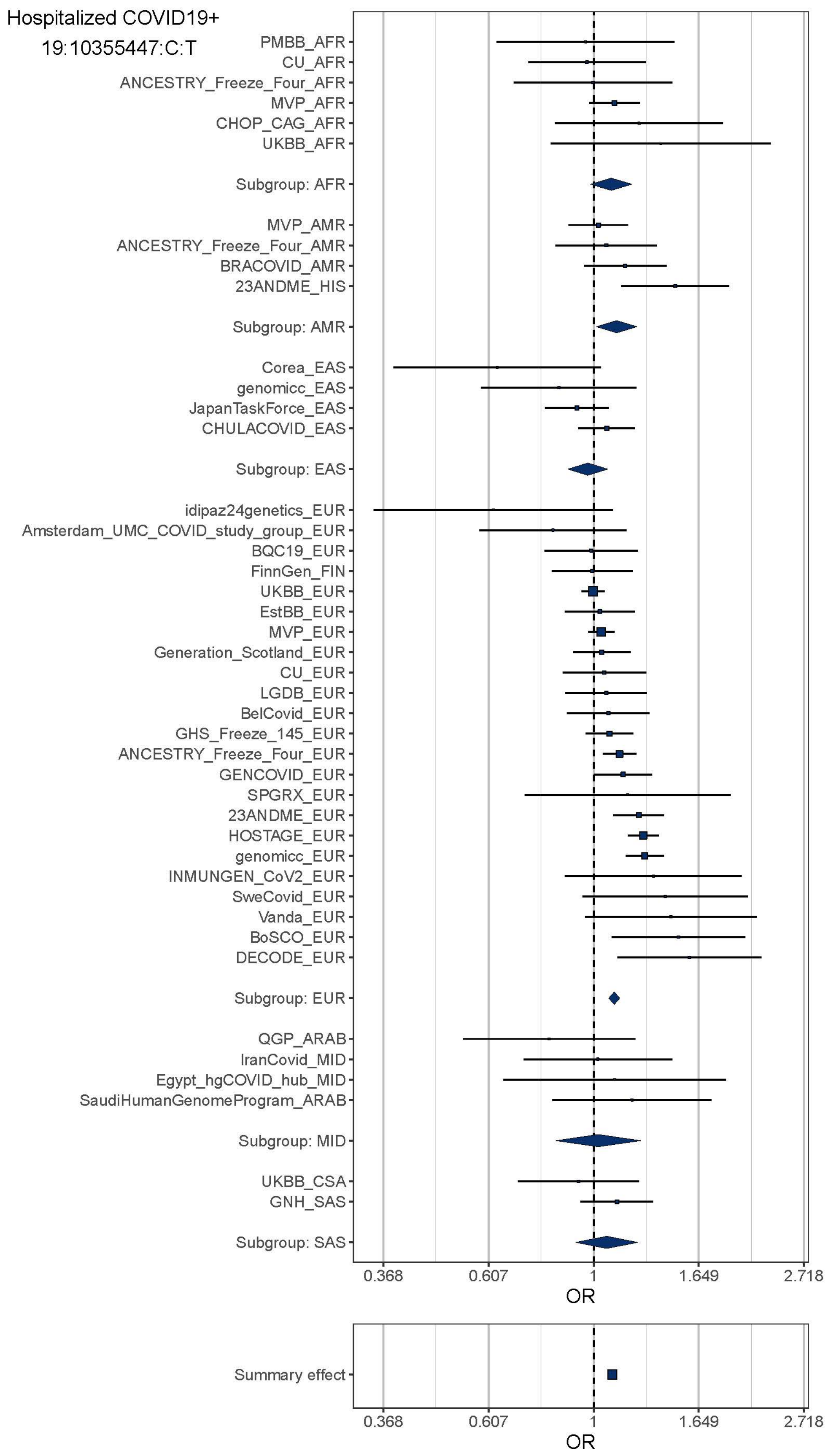
Hospitalized COVID19+
19:10355447:C:T
PMBB_AFR
CU_AFR
ANCESTRY_Freeze_Four_AFR
MVP_AFR
CHOP_CAG_AFR
UKBB_AFR
Subgroup: AFR
MVP_AMR
ANCESTRY_Freeze_Four_AMR
BRACOVID_AMR
23ANDME_HIS
Subgroup: AMR
Corea_EAS
genomicc_EAS
JapanTaskForce_EAS
CHULACOVID_EAS
Subgroup: EAS
idipaz24genetics_EUR
Amsterdam_UMC_COVID_study_group_EUR
BQC19_EUR
FinnGen_FIN
UKBB_EUR
EstBB_EUR
MVP_EUR
Generation_Scotland_EUR
CU_EUR
LGDB_EUR
BelCovid_EUR
GHS_Freeze_145_EUR
ANCESTRY_Freeze_Four_EUR
GENCOVID_EUR
SPGRX_EUR
23ANDME_EUR
HOSTAGE_EUR
genomicc_EUR
INMUNGEN_CoV2_EUR
SweCovid_EUR
Vanda_EUR
BoSCO_EUR
DECODE_EUR
Subgroup: EUR
QGP_ARAB
IranCovid_MID
Egypt_hgCOVID_hub_MID
SaudiHumanGenomeProgram_ARAB
Subgroup: MID
UKBB_CSA
GNH_SAS
Subgroup: SAS
0.368
0.607
1
1.649
2.718
OR
Summary effect
0.368
0.607
1
1.649
2.718
OR

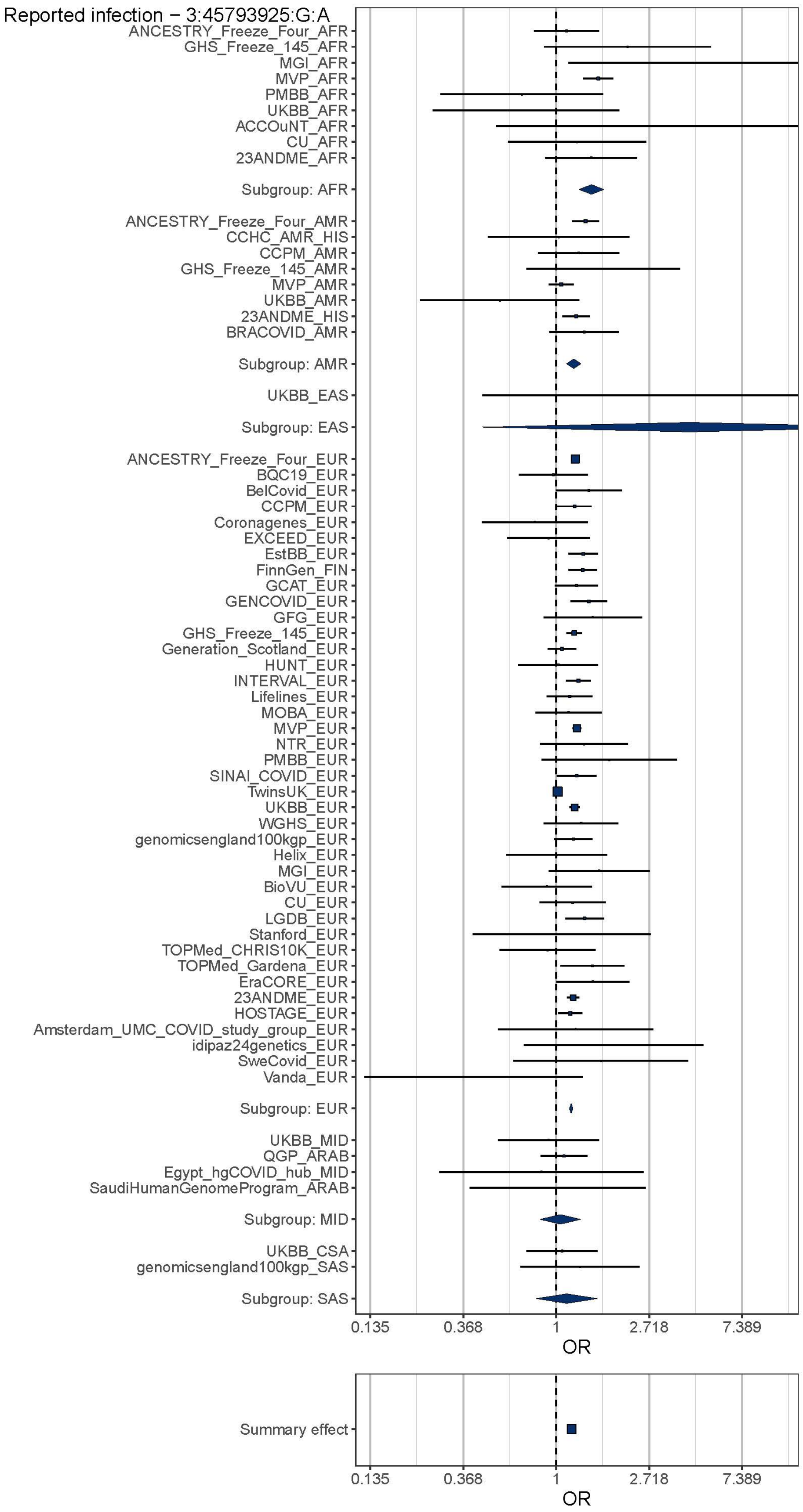
Reported infection − 3:45793925:G:A
ANCESTRY_Freeze_Four_AFR
GHS_Freeze_145_AFR
MGI_AFR
MVP_AFR
PMBB_AFR
UKBB_AFR
ACCOuNT_AFR
CU_AFR
23ANDME_AFR
Subgroup: AFR
ANCESTRY_Freeze_Four_AMR
CCHC_AMR_HIS
CCPM_AMR
GHS_Freeze_145_AMR
MVP_AMR
UKBB_AMR
23ANDME_HIS
BRACOVID_AMR
Subgroup: AMR
UKBB_EAS
Subgroup: EAS
ANCESTRY_Freeze_Four_EUR
BQC19_EUR
BelCovid_EUR
CCPM_EUR
Coronagenes_EUR
EXCEED_EUR
EstBB_EUR
FinnGen_FIN
GCAT_EUR
GENCOVID_EUR
GFG_EUR
GHS_Freeze_145_EUR
Generation_Scotland_EUR
HUNT_EUR
INTERVAL_EUR
Lifelines_EUR
MOBA_EUR
MVP_EUR
NTR_EUR
PMBB_EUR
SINAI_COVID_EUR
TwinsUK_EUR
UKBB_EUR
WGHS_EUR
genomicsengland100kgp_EUR
Helix_EUR
MGI_EUR
BioVU_EUR
CU_EUR
LGDB_EUR
Stanford_EUR
TOPMed_CHRIS10K_EUR
TOPMed_Gardena_EUR
EraCORE_EUR
23ANDME_EUR
HOSTAGE_EUR
Amsterdam_UMC_COVID_study_group_EUR
idipaz24genetics_EUR
SweCovid_EUR
Vanda_EUR
Subgroup: EUR
UKBB_MID
QGP_ARAB
Egypt_hgCOVID_hub_MID
SaudiHumanGenomeProgram_ARAB
Subgroup: MID
UKBB_CSA
genomicsengland100kgp_SAS
Subgroup: SAS
0.135
0.368
1
2.718
7.389
OR
Summary effect
0.135
0.368
1
2.718
7.389
OR

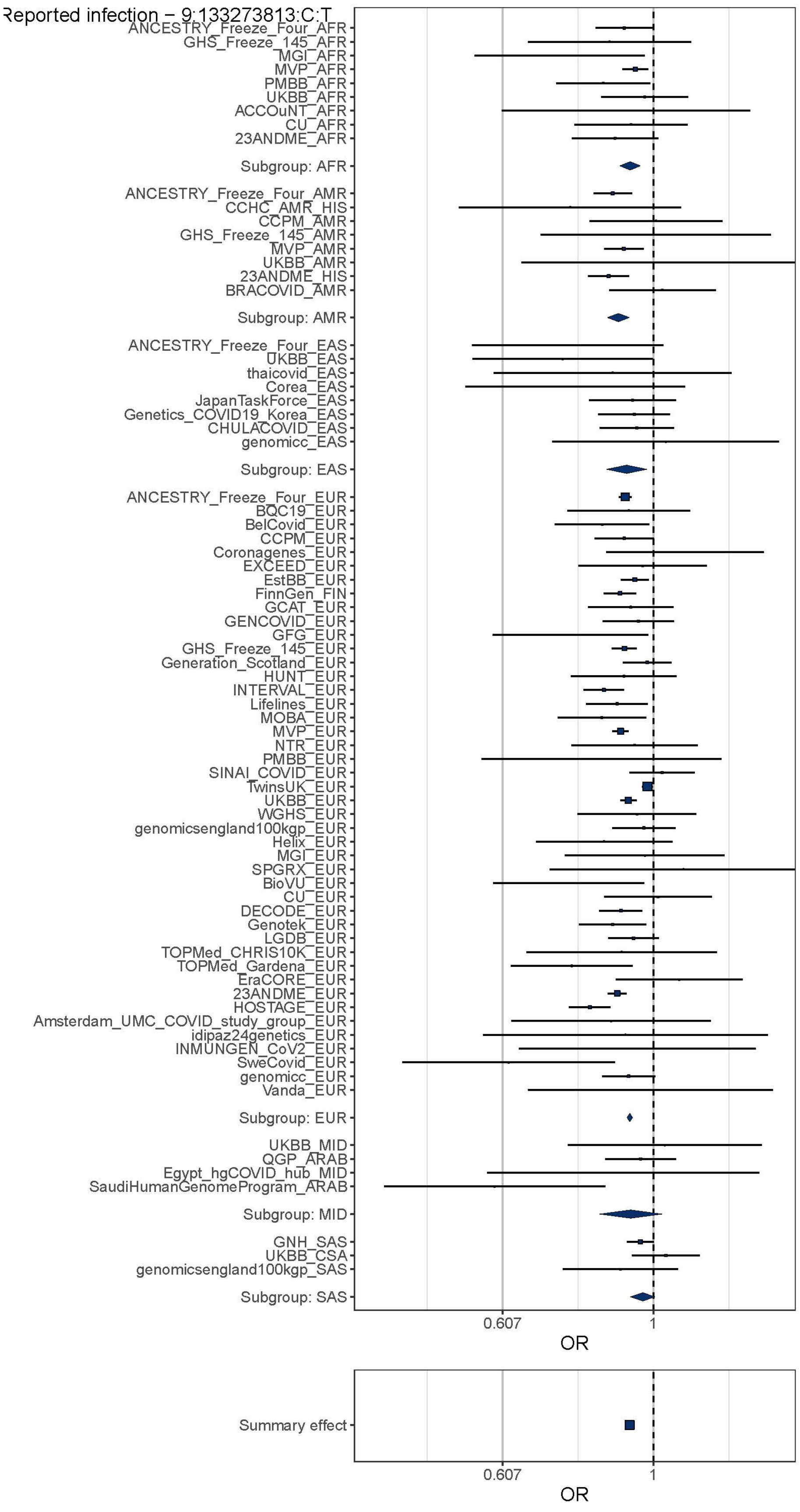
Reported infection – 9:133273813:C:T
ANCESTRY_Freeze_Four_AFR
GHS_Freeze_145_AFR
MGI_AFR
MVP_AFR
PMBB_AFR
UKBB_AFR
ACCOuNT_AFR
CU_AFR
23ANDME_AFR
Subgroup: AFR
ANCESTRY_Freeze_Four_AMR
CCHC_AMR_HIS
CCPM_AMR
GHS_Freeze_145_AMR
MVP_AMR
UKBB_AMR
23ANDME_HIS
BRACOVID_AMR
Subgroup: AMR
ANCESTRY_Freeze_Four_EAS
UKBB_EAS
thaicovid_EAS
Corea_EAS
JapanTaskForce_EAS
Genetics_COVID19_Korea_EAS
CHULACOVID_EAS
genomicc_EAS
Subgroup: EAS
ANCESTRY_Freeze_Four_EUR
BQC19_EUR
BelCovid_EUR
CCPM_EUR
Coronagenes_EUR
EXCEED_EUR
EstBB_EUR
FinnGen_FIN
GCAT_EUR
GENCOVID_EUR
GFG_EUR
GHS_Freeze_145_EUR
Generation_Scotland_EUR
HUNT_EUR
INTERVAL_EUR
Lifelines_EUR
MOBA_EUR
MVP_EUR
NTR_EUR
PMBB_EUR
SINAI_COVID_EUR
TwinsUK_EUR
UKBB_EUR
WGHS_EUR
genomicsengland100kgp_EUR
Helix_EUR
MGI_EUR
SPGRX_EUR
BioVU_EUR
CU_EUR
DECODE_EUR
Genotek_EUR
LGDB_EUR
TOPMed_CHRIS10K_EUR
TOPMed_Gardena_EUR
EraCORE_EUR
23ANDME_EUR
HOSTAGE_EUR
Amsterdam_UMC_COVID_study_group_EUR
idipaz24genetics_EUR
INMUNGEN_CoV2_EUR
SweCovid_EUR
genomicc_EUR
Vanda_EUR
Subgroup: EUR
UKBB_MID
QGP_ARAB
Egypt_hgCOVID_hub_MID
SaudiHumanGenomeProgram_ARAB
Subgroup: MID
GNH_SAS
UKBB_CSA
genomicsengland100kgp_SAS
Subgroup: SAS
0.607
1
OR
Summary effect
0.607
1
OR

Supplementary Figure 7

LocusZoom plots to visualize the meta-analysis results at the loci passing genome-wide significance. For each genome-wide significant locus in three meta-analyses: meta-analysis of critical illness, hospitalization, and reported infection, we showed 1) a Manhattan plot of each locus where a color represents a weighted-average r2 value (see COVID-19 Host Genetics Initiative, 2021) to a lead variant (unadjusted P-values from the two-tailed inverse variance weighted meta-analysis); 2) r2 values to a lead variant across gnomAD v2 populations, i.e., African/African-American (AFR), Latino/Admixed American (AMR), Ashkenazi Jewish (ASJ), East Asian (EAS), Estonian (EST), Finnish (FIN), Non-Finish Europeans (NFE), North-Western Europeans (NWE), and Southern Europeans (SEU); 3) genes at a locus; and 4) genes prioritized by each gene prioritization metric where a size of circles represents a rank in each metric. Note that the COVID-19 lead variants were chosen across all the meta-analyses (Supplementary Table 2) and were not necessarily a variant with the most significant P-value from each inverse variance weighted meta-analysis.

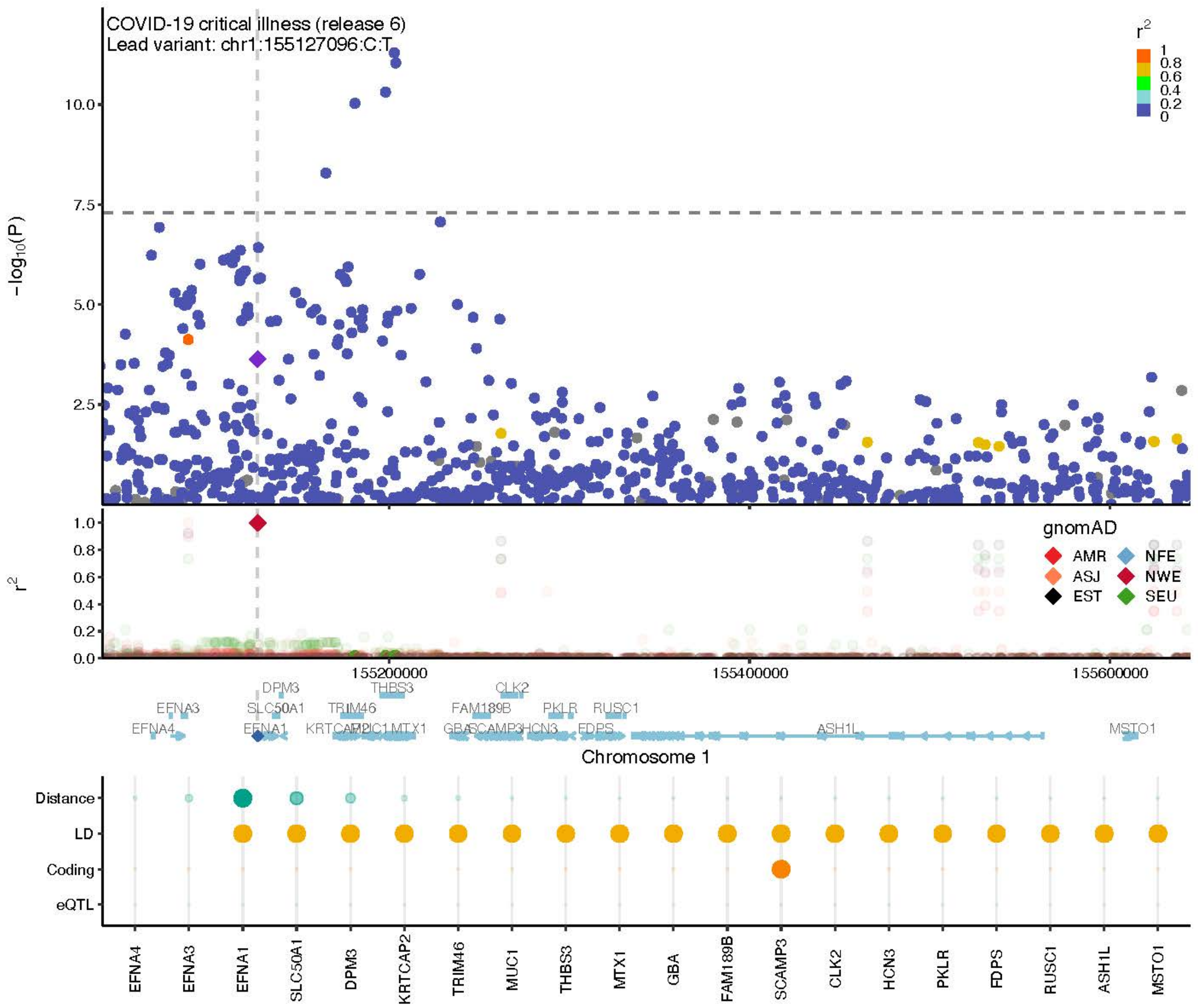
COVID-19 critical illness (release 6)
Lead variant: chr1:155127096:C:T
-log10(P)
r2
gnomAD
AMR
ASJ
EST
NFE
NWE
SEU
155200000
155400000
155600000
Chromosome 1
Distance
LD
Coding
eQTL
EFNA4
EFNA3
EFNA1
SLC50A1
DPM3
KRTCAP2
TRIM46
MUC1
THBS3
MTX1
GBA
FAM189B
SCAMP3
CLK2
HCN3
PKLR
FDPS
RUSC1
ASH1L
MSTO1

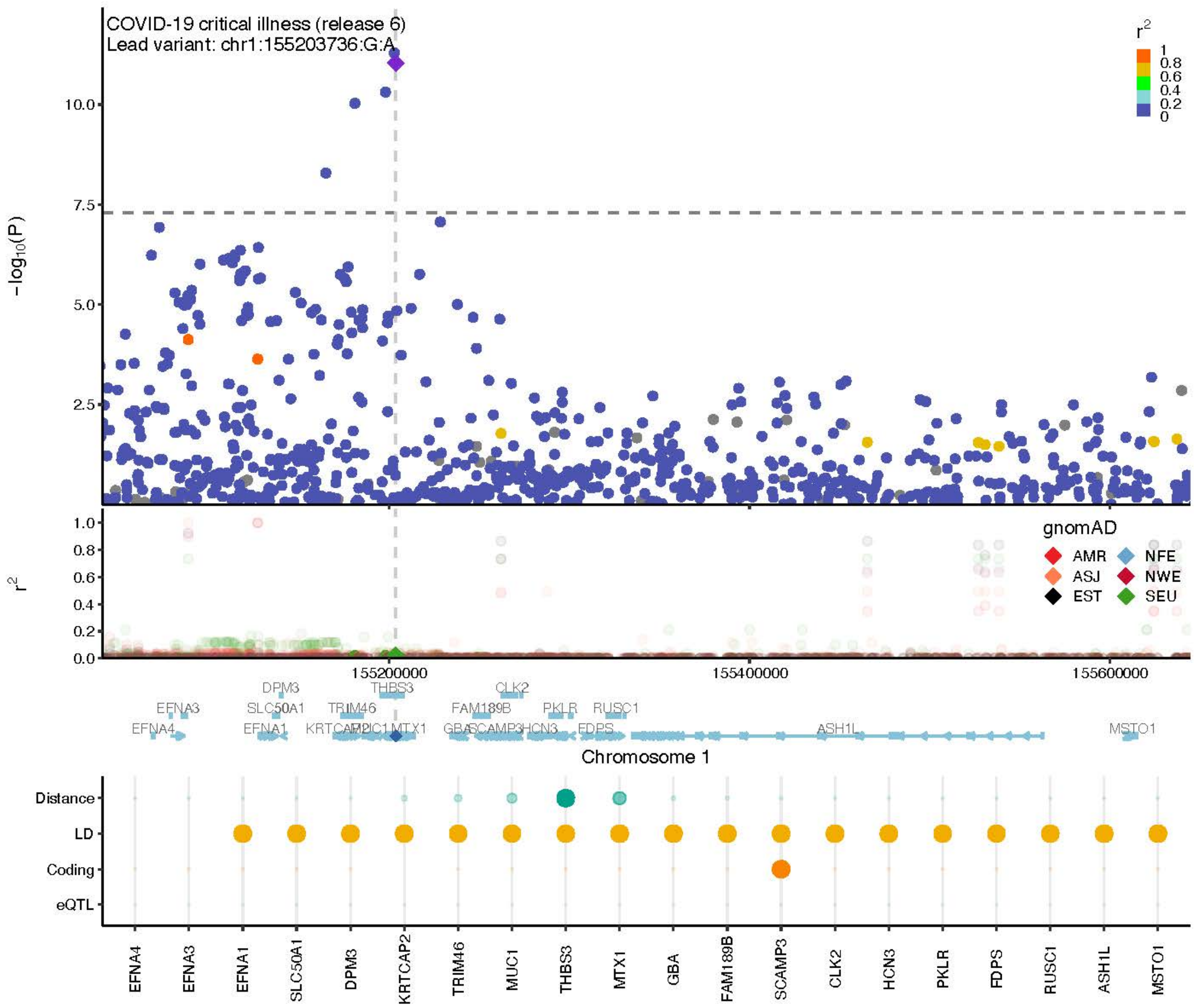
COVID-19 critical illness (release 6)
Lead variant: chr1:155203736:G:A
−log10(P)
r2
gnomAD
AMR
ASJ
EST
NFE
NWE
SEU
Chromosome 1
Distance
LD
Coding
eQTL
EFNA4
EFNA3
EFNA1
SLC50A1
DPM3
KRTCAP2
TRIM46
MUC1
THBS3
MTX1
GBA
FAM189B
SCAMP3
CLK2
HCN3
PKLR
FDPS
RUSC1
ASH1L
MSTO1

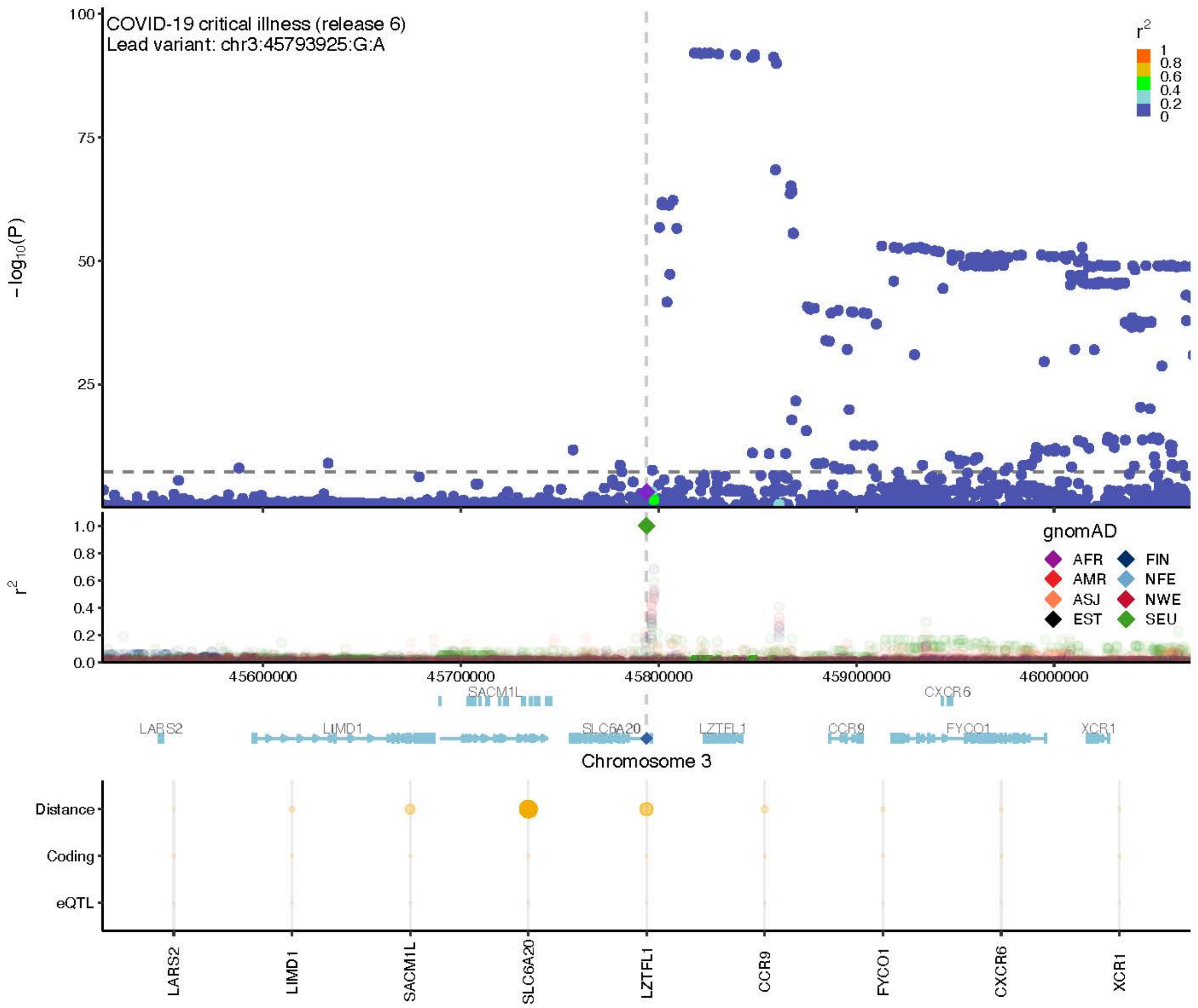
COVID-19 critical illness (release 6)
Lead variant: chr3:45793925:G:A
−log10(P)
100
75
50
25
r2
1
0.8
0.6
0.4
0.2
0
1.0
0.8
0.6
0.4
0.2
0.0
gnomAD
AFR
AMR
ASJ
EST
FIN
NFE
NWE
SEU
45600000
45700000
45800000
45900000
46000000
SACM1L
CXCR6
LARS2
LIMD1
SLC6A20
LZTFL1
CCR9
FYCO1
XCR1
Chromosome 3
Distance
Coding
eQTL
LARS2
LIMD1
SACM1L
SLC6A20
LZTFL1
CCR9
FYCO1
CXCR6
XCR1

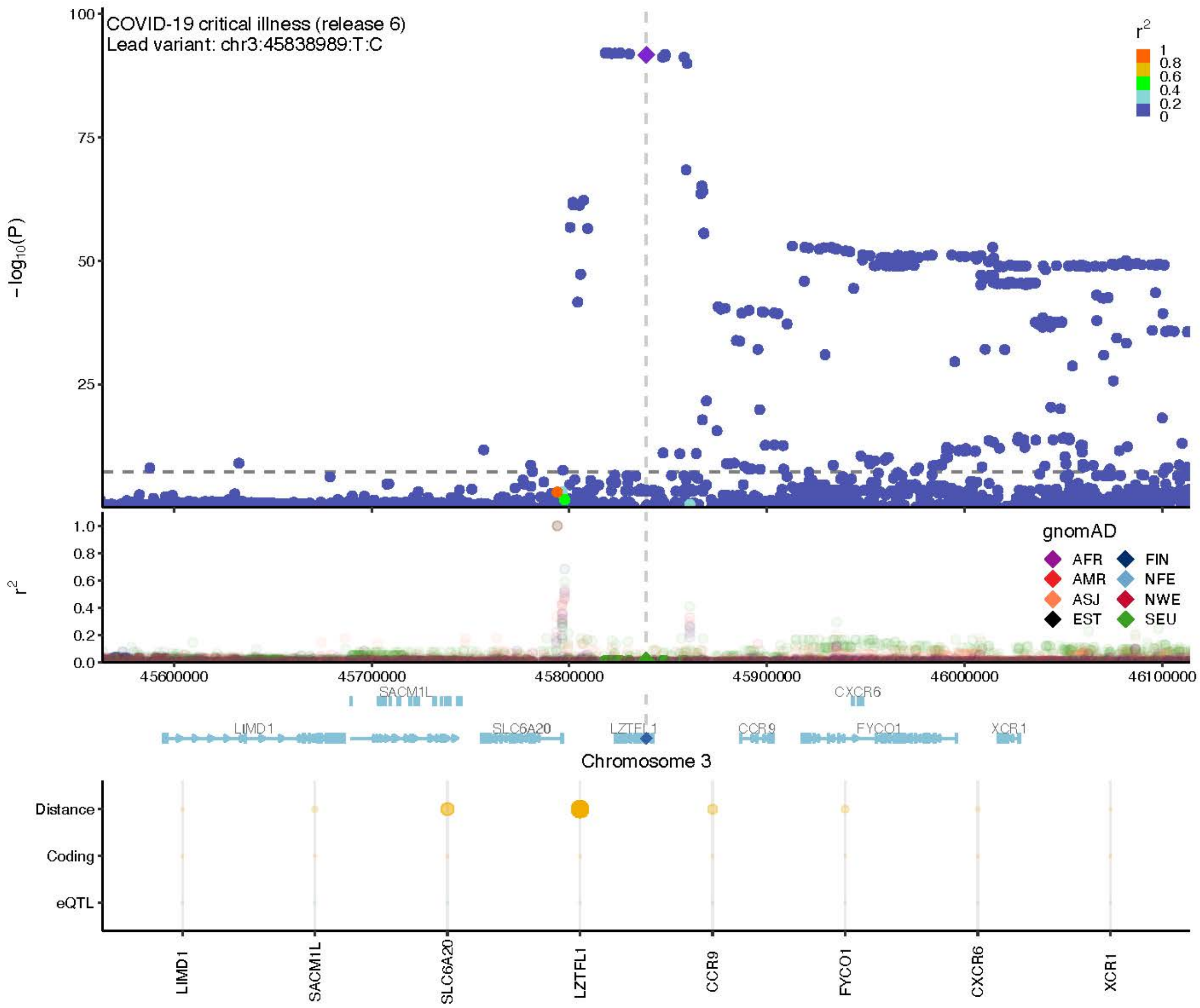
COVID-19 critical illness (release 6)
Lead variant: chr3:45838989:T:C
$-\log_{10}(P)$
r^2
gnomAD
AFR
AMR
ASJ
EST
FIN
NFE
NWE
SEU
SACM1L
CXCR6
LIMD1
SLC6A20
LZTFL1
CCR9
FYCO1
XCR1
Chromosome 3
Distance
Coding
eQTL
LIMD1
SACM1L
SLC6A20
LZTFL1
CCR9
FYCO1
CXCR6
XCR1

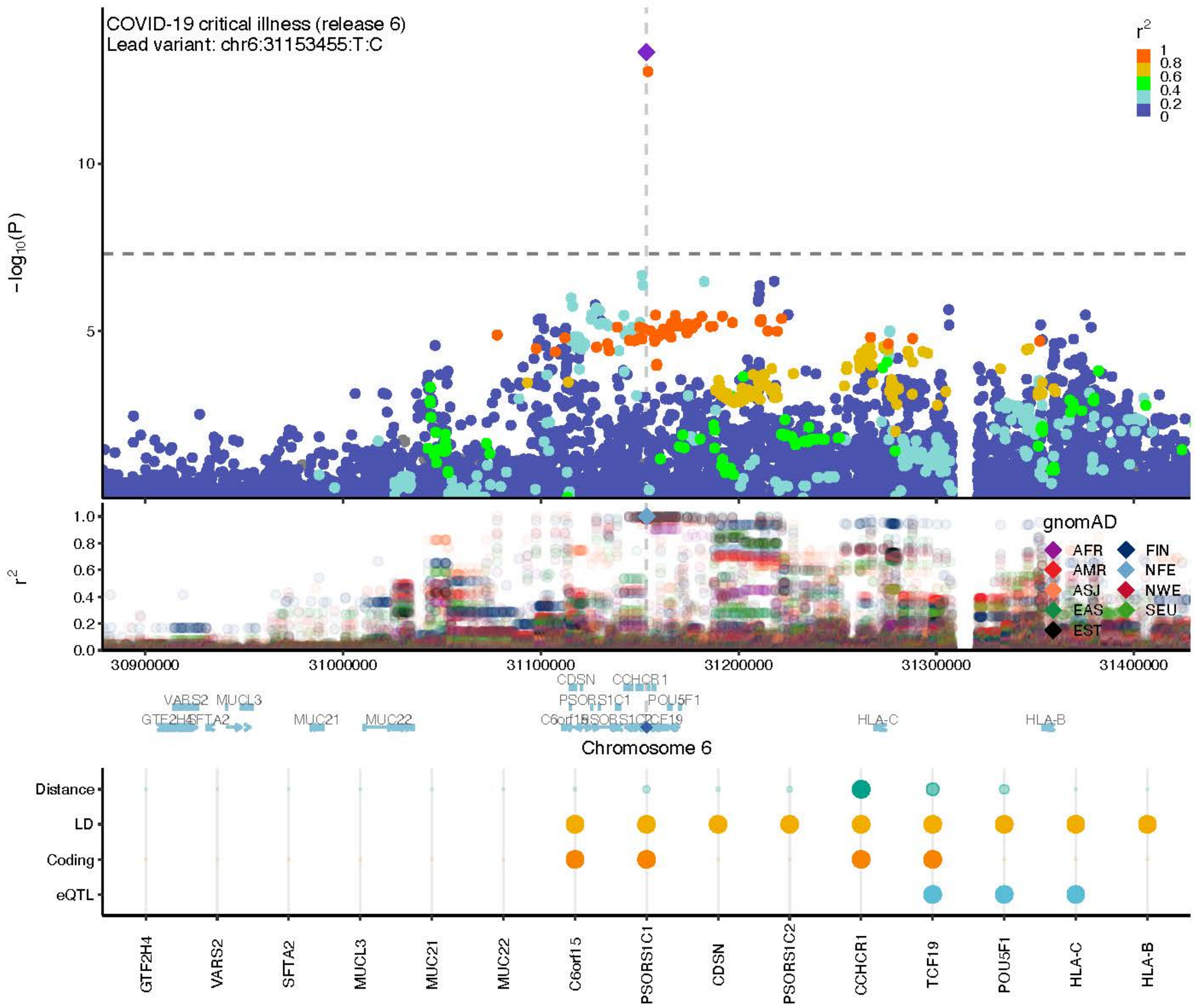
COVID-19 critical illness (release 6)
Lead variant: chr6:31153455:T:C
$-\log_{10}(P)$
10
5
r^2
1
0.8
0.6
0.4
0.2
0
r^2
1.0
0.8
0.6
0.4
0.2
0.0
gnomAD
AFR
AMR
ASJ
EAS
EST
FIN
NFE
NWE
SEU
30900000
31000000
31100000
31200000
31300000
31400000
CDSN
CCHCR1
VARS2
MUCL3
PSORS1C1
POU5F1
GTF2H4
SFTA2
MUC21
MUC22
HLA-C
HLA-B
Chromosome 6
Distance
LD
Coding
eQTL
GTF2H4
VARS2
SFTA2
MUCL3
MUC21
MUC22
C6orf15
PSORS1C1
CDSN
PSORS1C2
CCHCR1
TCF19
POU5F1
HLA-C
HLA-B

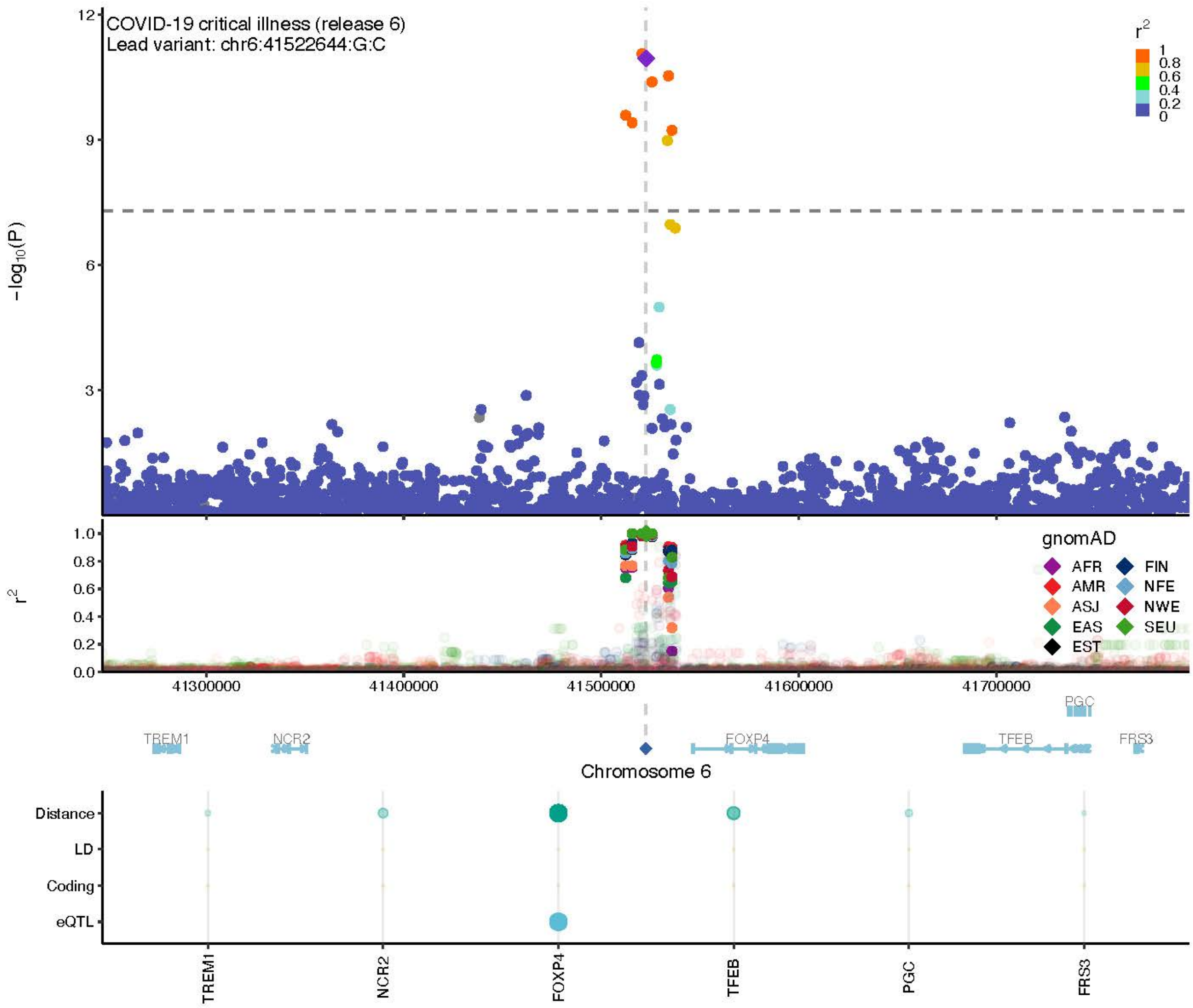
COVID-19 critical illness (release 6)
Lead variant: chr6:41522644:G:C
-log10(P)
r2
1
0.8
0.6
0.4
0.2
0
gnomAD
AFR
AMR
ASJ
EAS
EST
FIN
NFE
NWE
SEU
41300000
41400000
41500000
41600000
41700000
TREM1
NCR2
FOXP4
TFEB
PGC
FRS3
Chromosome 6
Distance
LD
Coding
eQTL

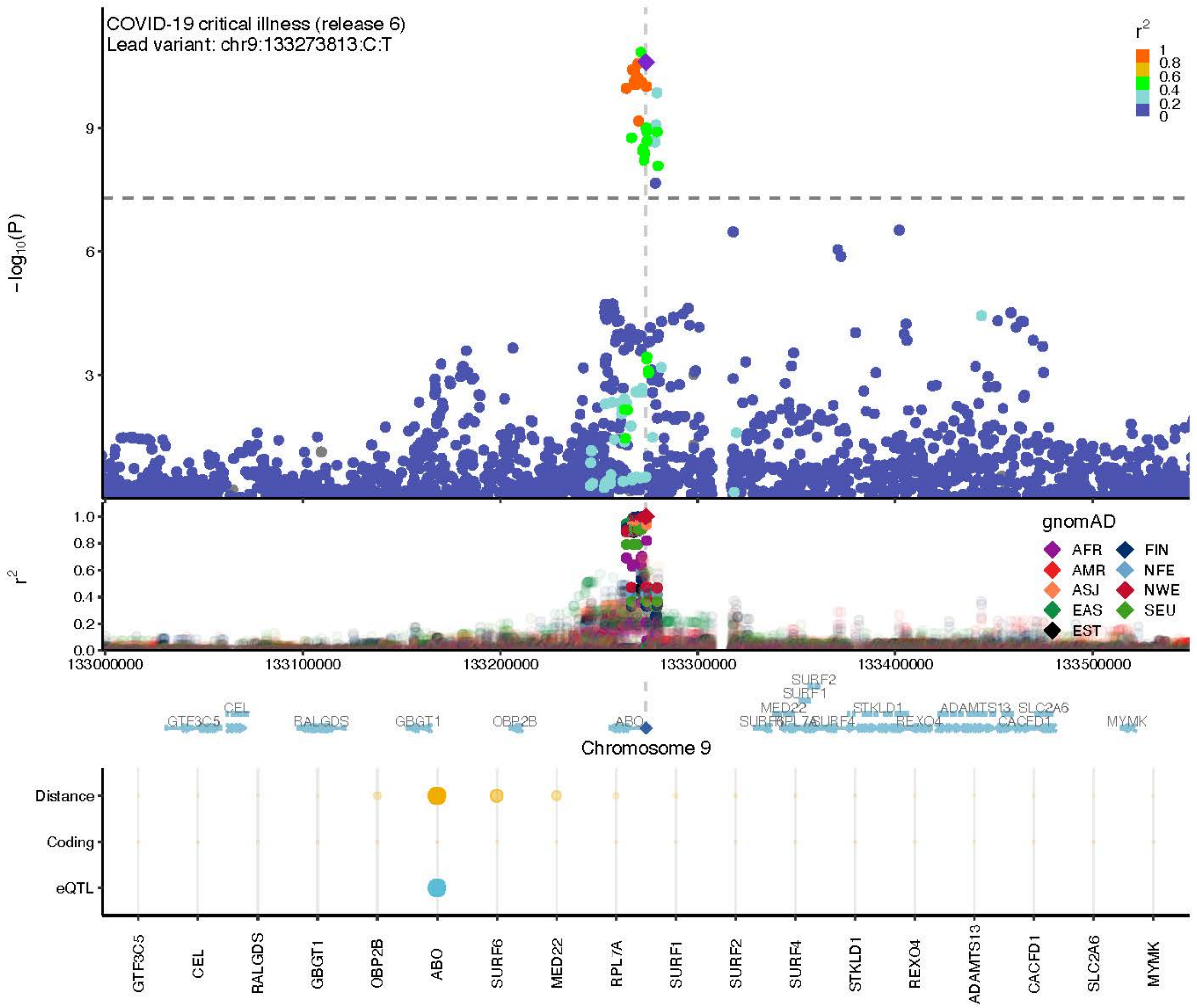
COVID-19 critical illness (release 6)
Lead variant: chr9:133273813:C:T
−log10(P)
r2
gnomAD
AFR
AMR
ASJ
EAS
EST
FIN
NFE
NWE
SEU
Chromosome 9
Distance
Coding
eQTL
GTF3C5
CEL
RALGDS
GBGT1
OBP2B
ABO
SURF6
MED22
RPL7A
SURF1
SURF2
SURF4
STKLD1
REXO4
ADAMTS13
CACFD1
SLC2A6
MYMK

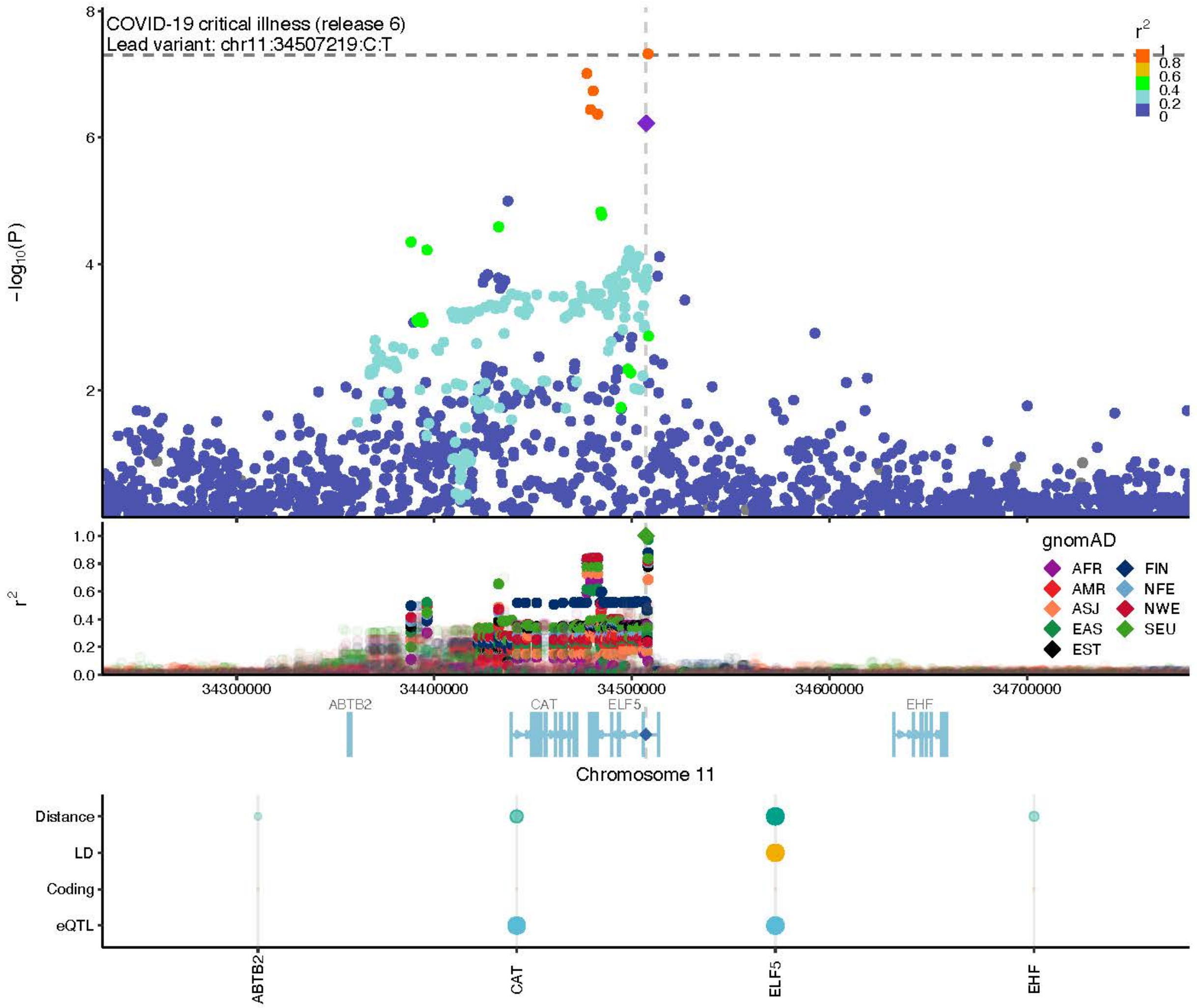
COVID-19 critical illness (release 6)
Lead variant: chr11:34507219:C:T
-log10(P)
r2
1
0.8
0.6
0.4
0.2
0
gnomAD
AFR
AMR
ASJ
EAS
EST
FIN
NFE
NWE
SEU
34300000
34400000
34500000
34600000
34700000
ABTB2
CAT
ELF5
EHF
Chromosome 11
Distance
LD
Coding
eQTL
ABTB2
CAT
ELF5
EHF

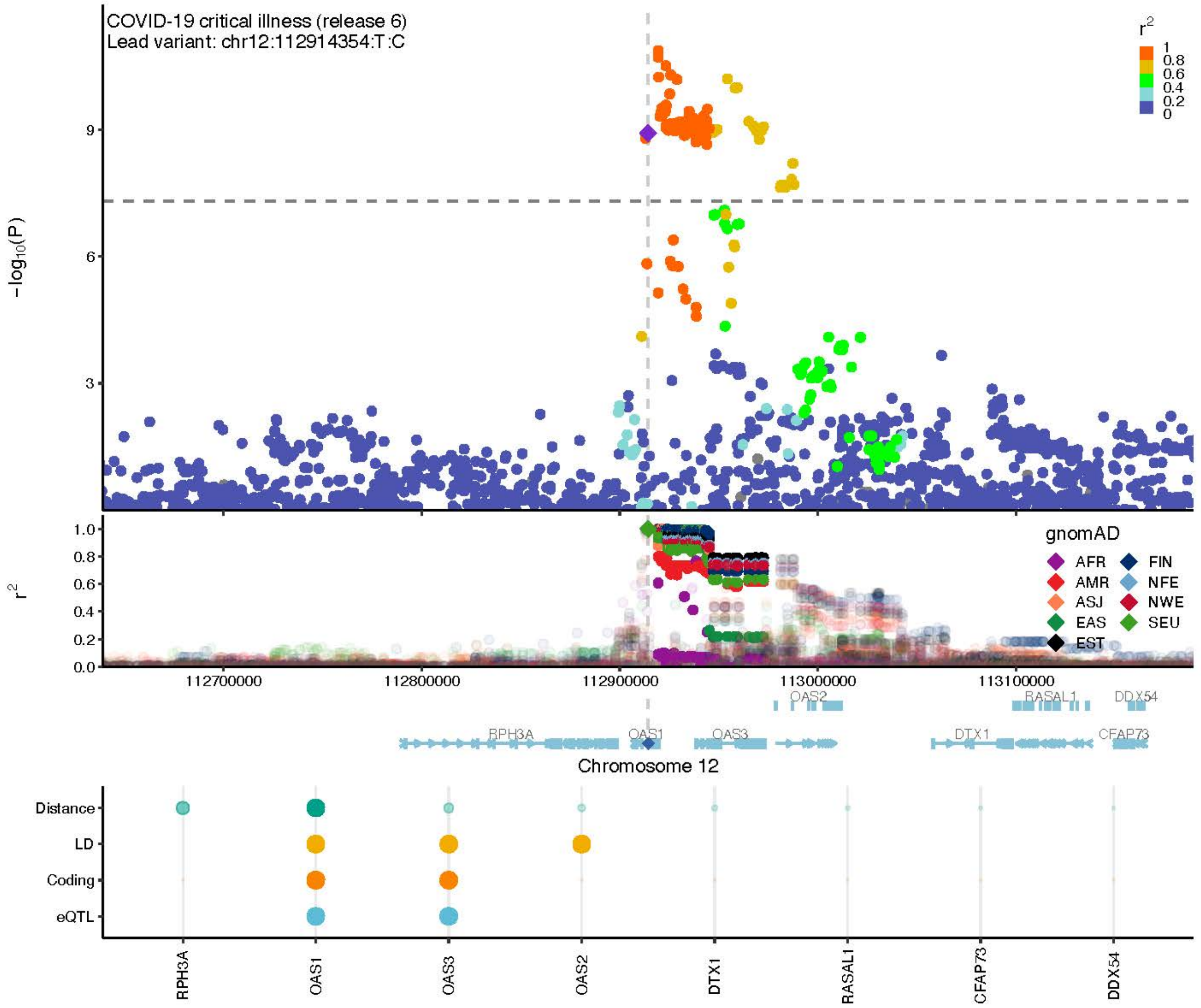
COVID-19 critical illness (release 6)
Lead variant: chr12:112914354:T:C
-log10(P)
r2
gnomAD
AFR
FIN
AMR
NFE
ASJ
NWE
EAS
SEU
EST
112700000
112800000
112900000
113000000
113100000
OAS2
RASAL1
DDX54
RPH3A
OAS1
OAS3
DTX1
CFAP73
Chromosome 12
Distance
LD
Coding
eQTL
RPH3A
OAS1
OAS3
OAS2
DTX1
RASAL1
CFAP73
DDX54

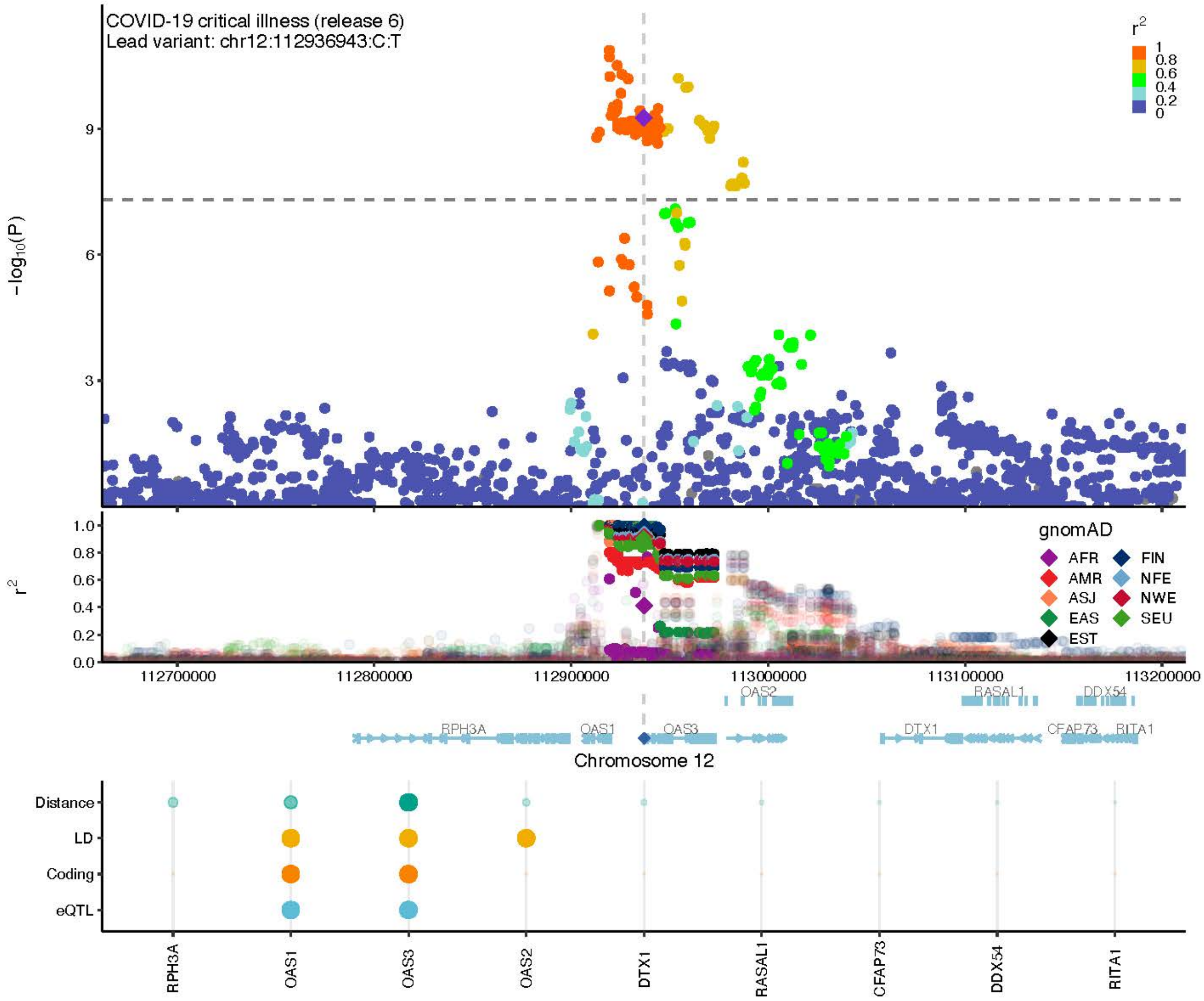
COVID-19 critical illness (release 6)
Lead variant: chr12:112936943:C:T
-log10(P)
r2
gnomAD
AFR
AMR
ASJ
EAS
EST
FIN
NFE
NWE
SEU
112700000
112800000
112900000
113000000
113100000
113200000
Chromosome 12
RPH3A
OAS1
OAS3
OAS2
DTX1
RASAL1
CFAP73
DDX54
RITA1
Distance
LD
Coding
eQTL

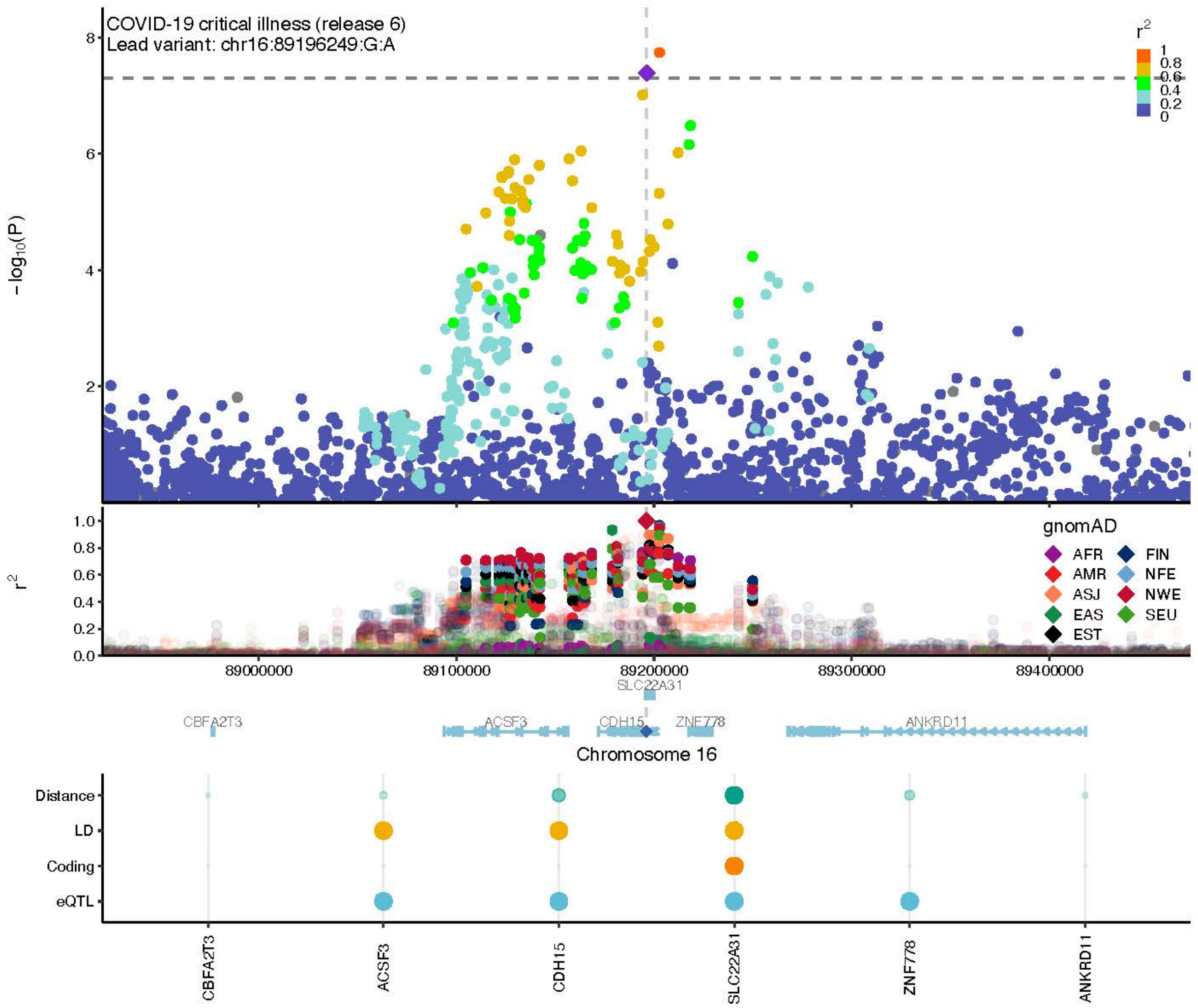
COVID-19 critical illness (release 6)
Lead variant: chr16:89196249:G:A
-log10(P)
r2
1
0.8
0.6
0.4
0.2
0
gnomAD
AFR
AMR
ASJ
EAS
EST
FIN
NFE
NWE
SEU
89000000
89100000
89200000
89300000
89400000
SLC22A31
CBFA2T3
ACSF3
CDH15
ZNF778
ANKRD11
Chromosome 16
Distance
LD
Coding
eQTL
CBFA2T3
ACSF3
CDH15
SLC22A31
ZNF778
ANKRD11

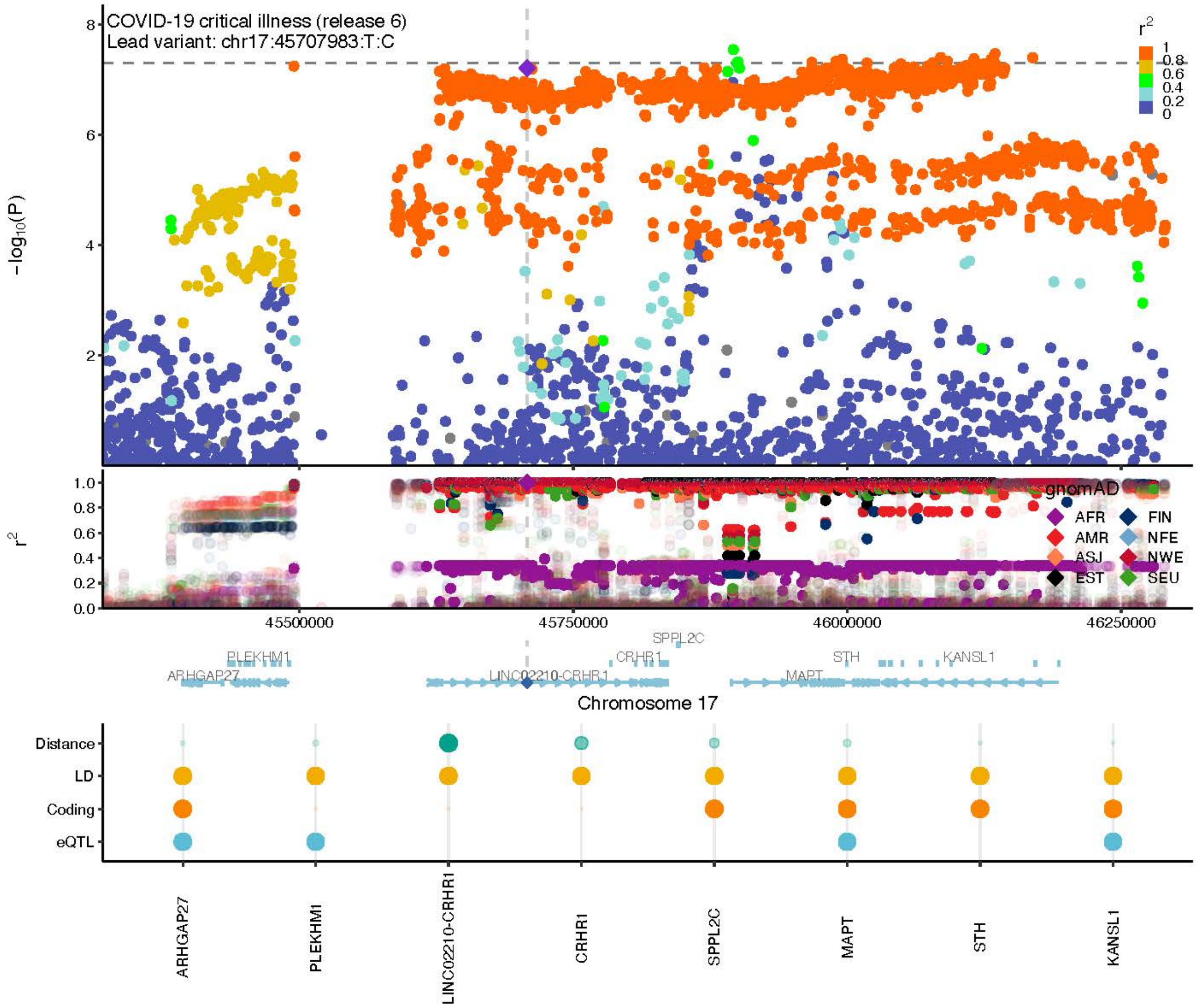
COVID-19 critical illness (release 6)
Lead variant: chr17:45707983:T:C
-log10(P)
r2
1
0.8
0.6
0.4
0.2
0
gnomAD
AFR
FIN
AMR
NFE
ASJ
NWE
EST
SEU
45500000
45750000
46000000
46250000
SPPL2C
PLEKHM1
CRHR1
STH
KANSL1
ARHGAP27
LINC02210-CRHR1
MAPT
Chromosome 17
Distance
LD
Coding
eQTL
ARHGAP27
PLEKHM1
LINC02210-CRHR1
CRHR1
SPPL2C
MAPT
STH
KANSL1

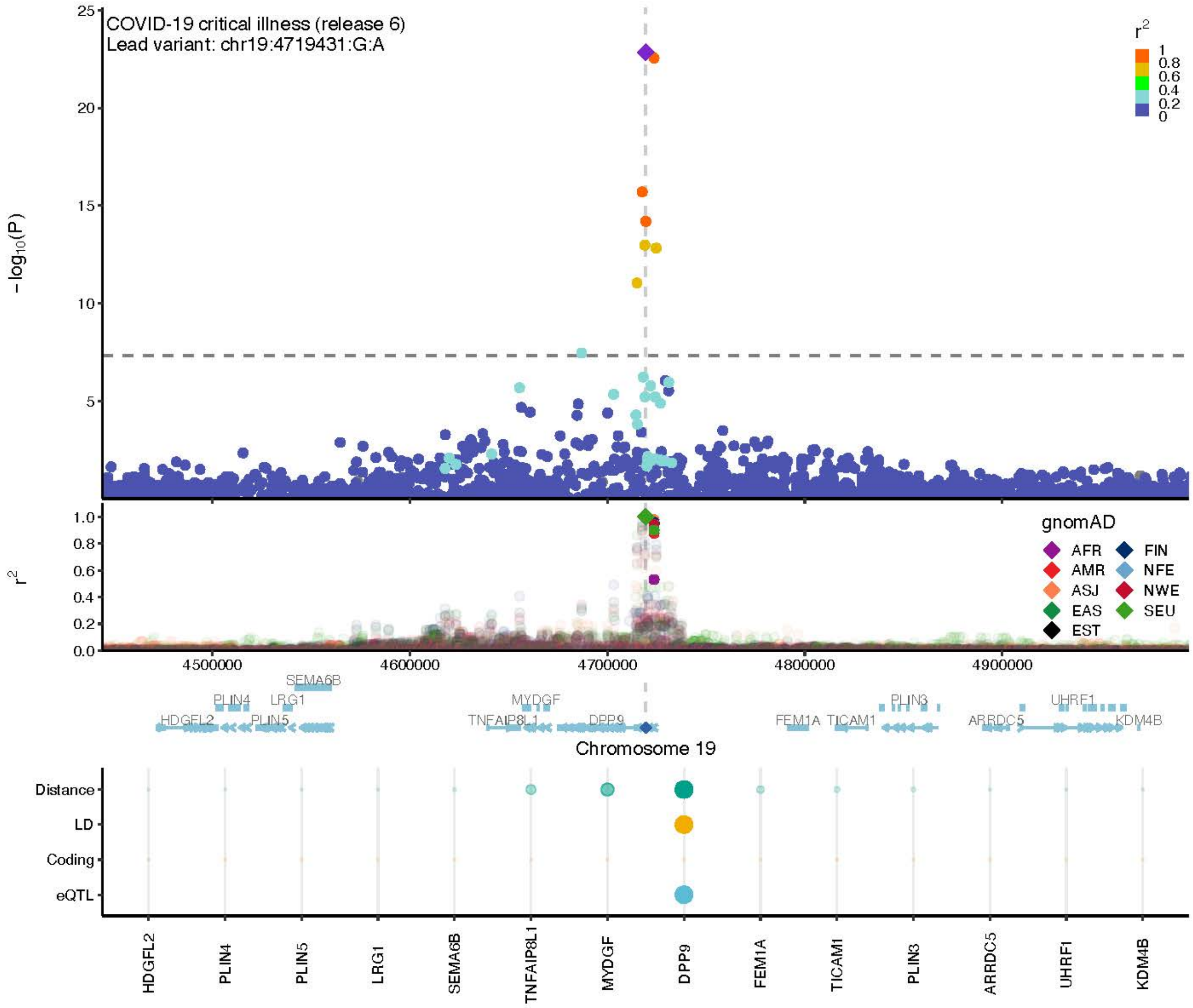
COVID-19 critical illness (release 6)
Lead variant: chr19:4719431:G:A
$-\log_{10}(P)$
r^2
gnomAD
AFR
AMR
ASJ
EAS
EST
FIN
NFE
NWE
SEU
Chromosome 19
Distance
LD
Coding
eQTL
HDGFL2
PLIN4
PLIN5
LRG1
SEMA6B
TNFAIP8L1
MYDGF
DPP9
FEM1A
TICAM1
PLIN3
ARRDC5
UHRF1
KDM4B

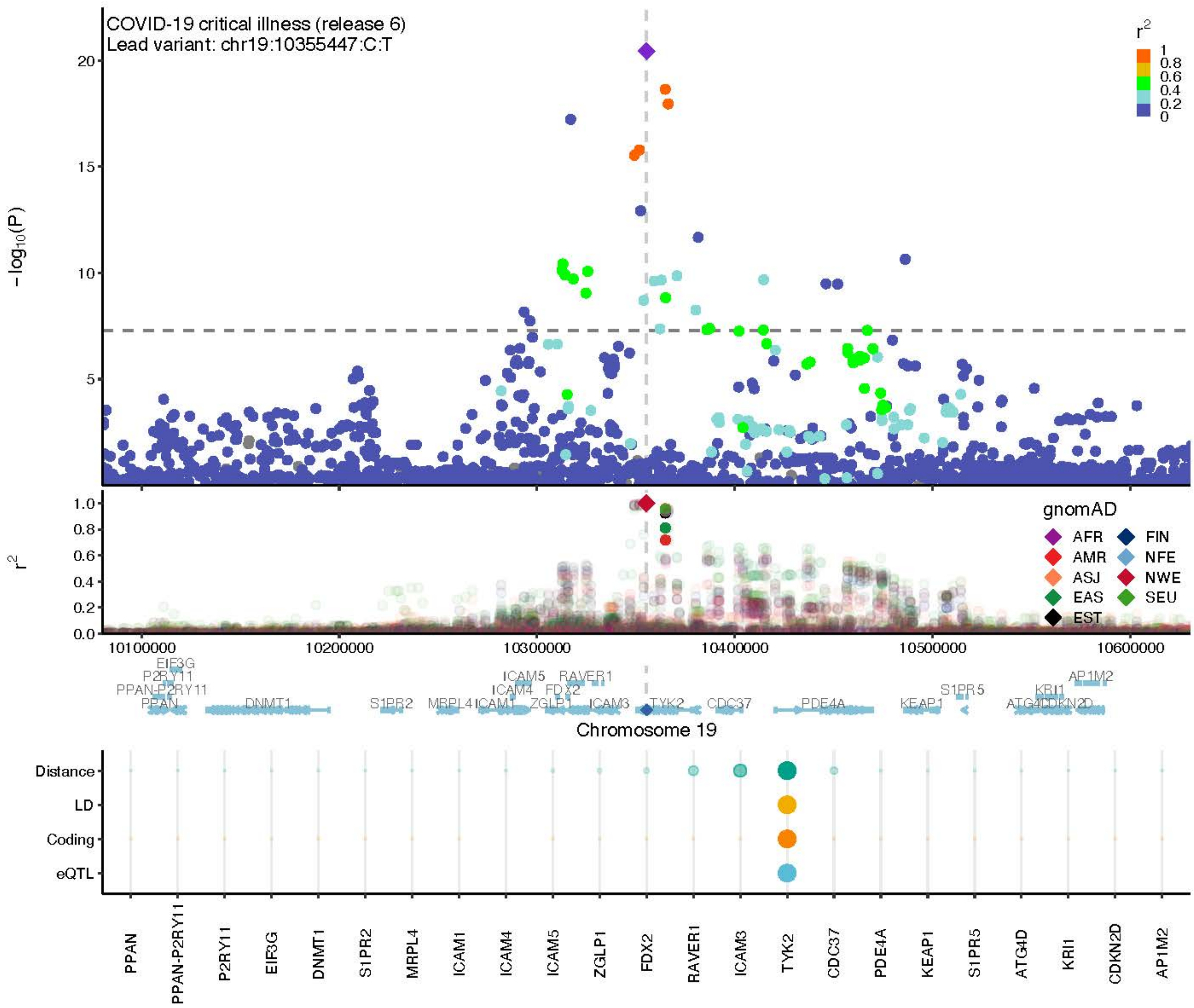
COVID-19 critical illness (release 6)
Lead variant: chr19:10355447:C:T
-log10(P)
r2
gnomAD
AFR
AMR
ASJ
EAS
EST
FIN
NFE
NWE
SEU
Chromosome 19
Distance
LD
Coding
eQTL
PPAN
PPAN-P2RY11
P2RY11
EIF3G
DNMT1
S1PR2
MRPL4
ICAM1
ICAM4
ICAM5
ZGLP1
FDX2
RAVER1
ICAM3
TYK2
CDC37
PDE4A
KEAP1
S1PR5
ATG4D
KRI1
CDKN2D
AP1M2

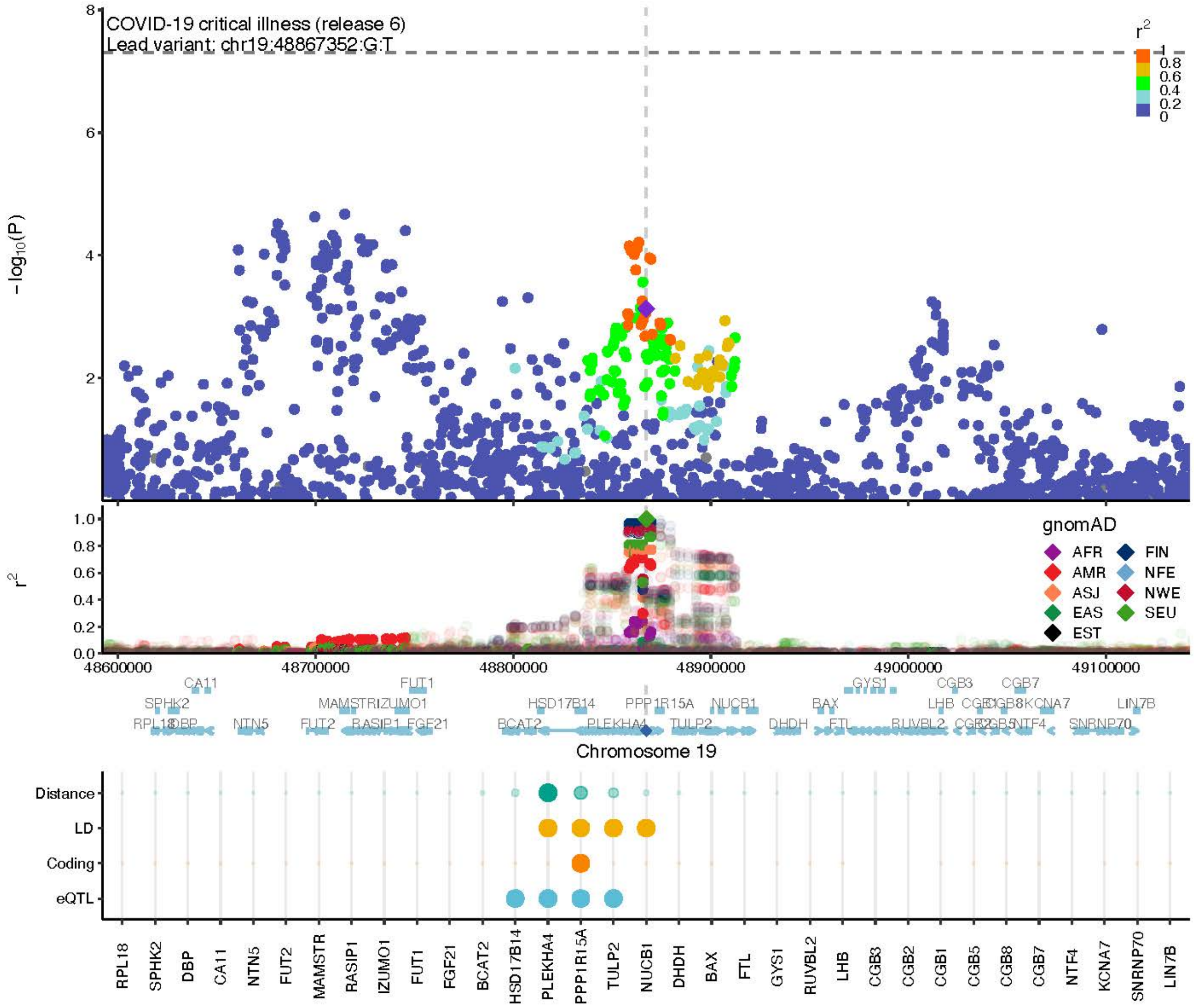
COVID-19 critical illness (release 6)
Lead variant: chr19:48867352:G:T
-log10(P)
r2
gnomAD
AFR
FIN
AMR
NFE
ASJ
NWE
EAS
SEU
EST
48600000
48700000
48800000
48900000
49000000
49100000
Chromosome 19
Distance
LD
Coding
eQTL
RPL18
SPHK2
DBP
CA11
NTN5
FUT2
MAMSTR
RASIP1
IZUMO1
FUT1
FGF21
BCAT2
HSD17B14
PLEKHA4
PPP1R15A
TULP2
NUCB1
DHDH
BAX
FTL
GYS1
RUVBL2
LHB
CGB3
CGB2
CGB1
CGB5
CGB8
CGB7
NTF4
KCNA7
SNRNP70
LIN7B

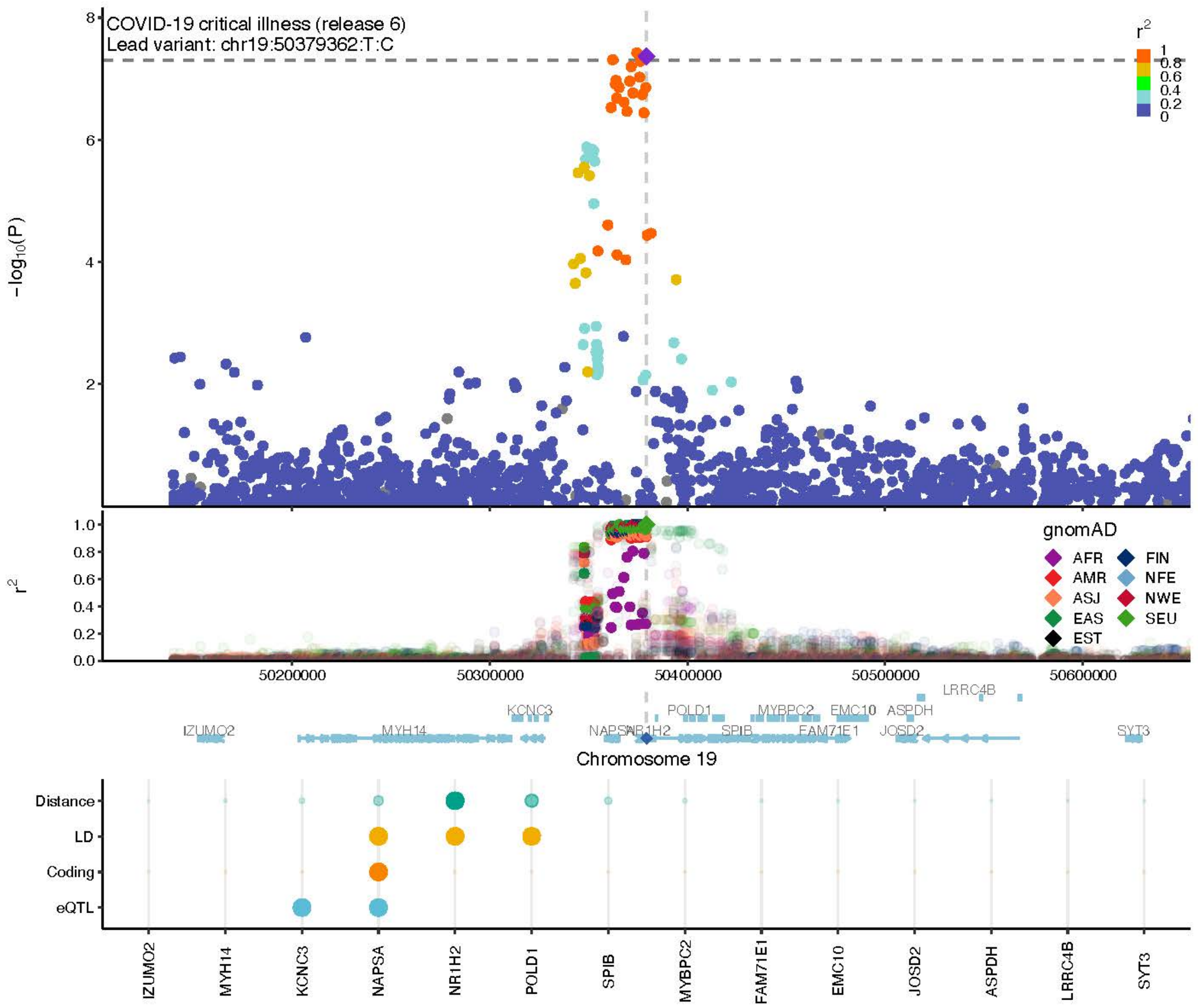
COVID-19 critical illness (release 6)
Lead variant: chr19:50379362:T:C
-log10(P)
r2
gnomAD
AFR
AMR
ASJ
EAS
EST
FIN
NFE
NWE
SEU
50200000
50300000
50400000
50500000
50600000
Chromosome 19
IZUMO2
MYH14
KCNC3
NAPSA
NR1H2
POLD1
SPIB
MYBPC2
FAM71E1
EMC10
JOSD2
ASPDH
LRRC4B
SYT3
Distance
LD
Coding
eQTL

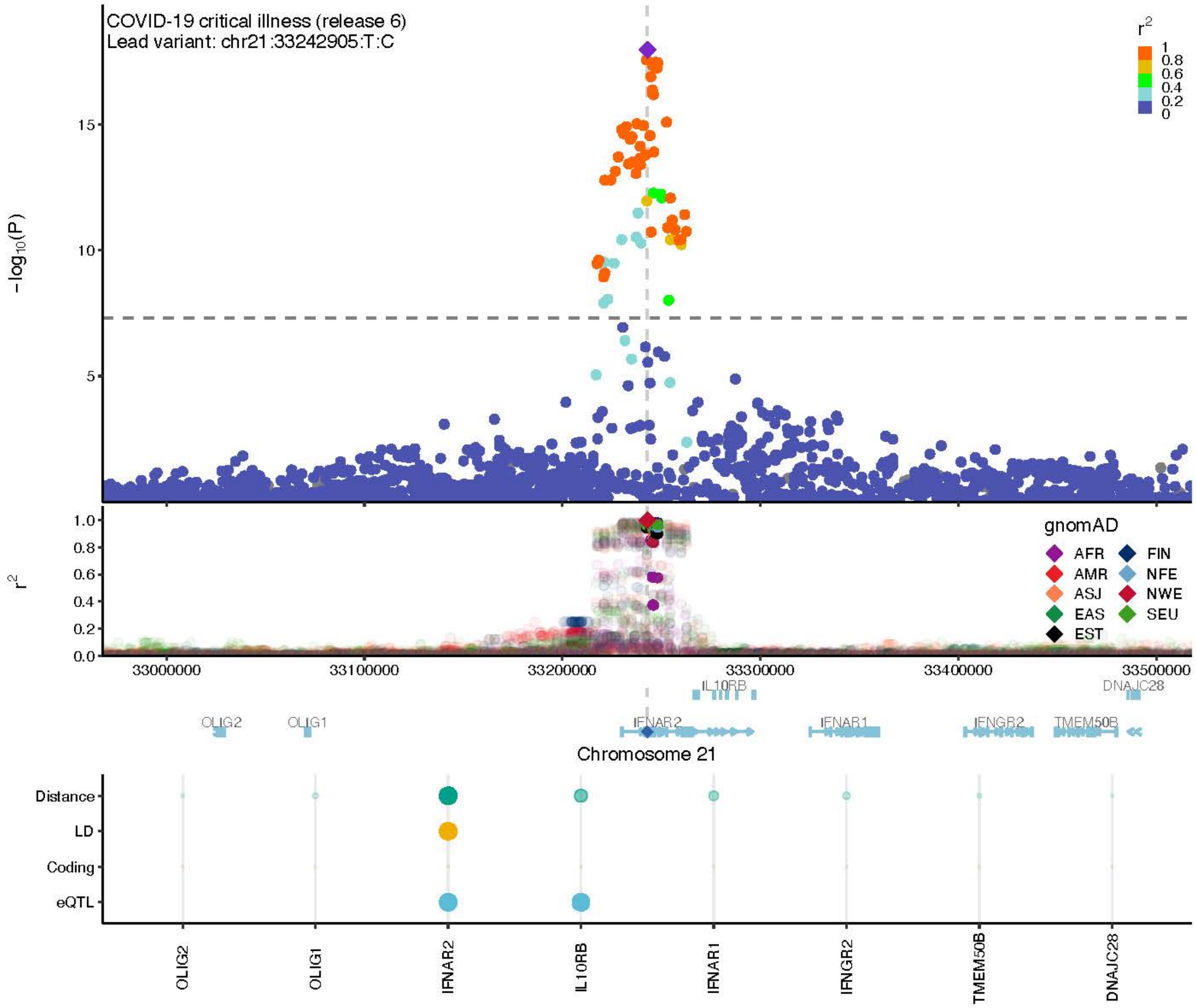
COVID-19 critical illness (release 6)
Lead variant: chr21:33242905:T:C
$-\log_{10}(P)$
15
10
5
r^2
1
0.8
0.6
0.4
0.2
0
r^2
1.0
0.8
0.6
0.4
0.2
0.0
gnomAD
AFR
AMR
ASJ
EAS
EST
FIN
NFE
NWE
SEU
33000000
33100000
33200000
33300000
33400000
33500000
IL10RB
DNAJC28
OLIG2
OLIG1
IFNAR2
IFNAR1
IFNGR2
TMEM50B
Chromosome 21
Distance
LD
Coding
eQTL
OLIG2
OLIG1
IFNAR2
IL10RB
IFNAR1
IFNGR2
TMEM50B
DNAJC28

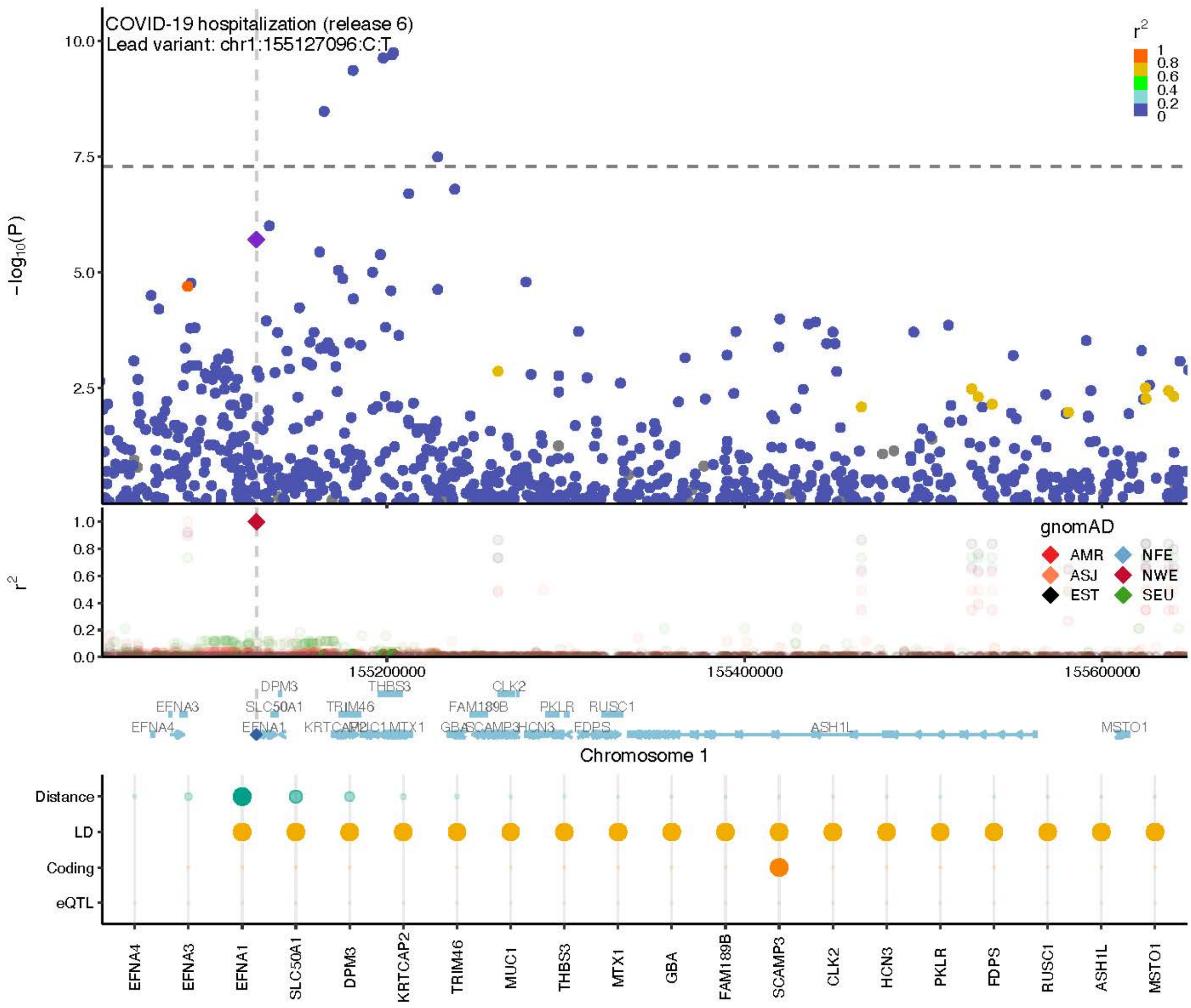
COVID-19 hospitalization (release 6)
Lead variant: chr1:155127096:C:T
−log10(P)
r2
gnomAD
AMR
ASJ
EST
NFE
NWE
SEU
155200000
155400000
155600000
Chromosome 1
Distance
LD
Coding
eQTL
EFNA4
EFNA3
EFNA1
SLC50A1
DPM3
KRTCAP2
TRIM46
MUC1
THBS3
MTX1
GBA
FAM189B
SCAMP3
CLK2
HCN3
PKLR
FDPS
RUSC1
ASH1L
MSTO1

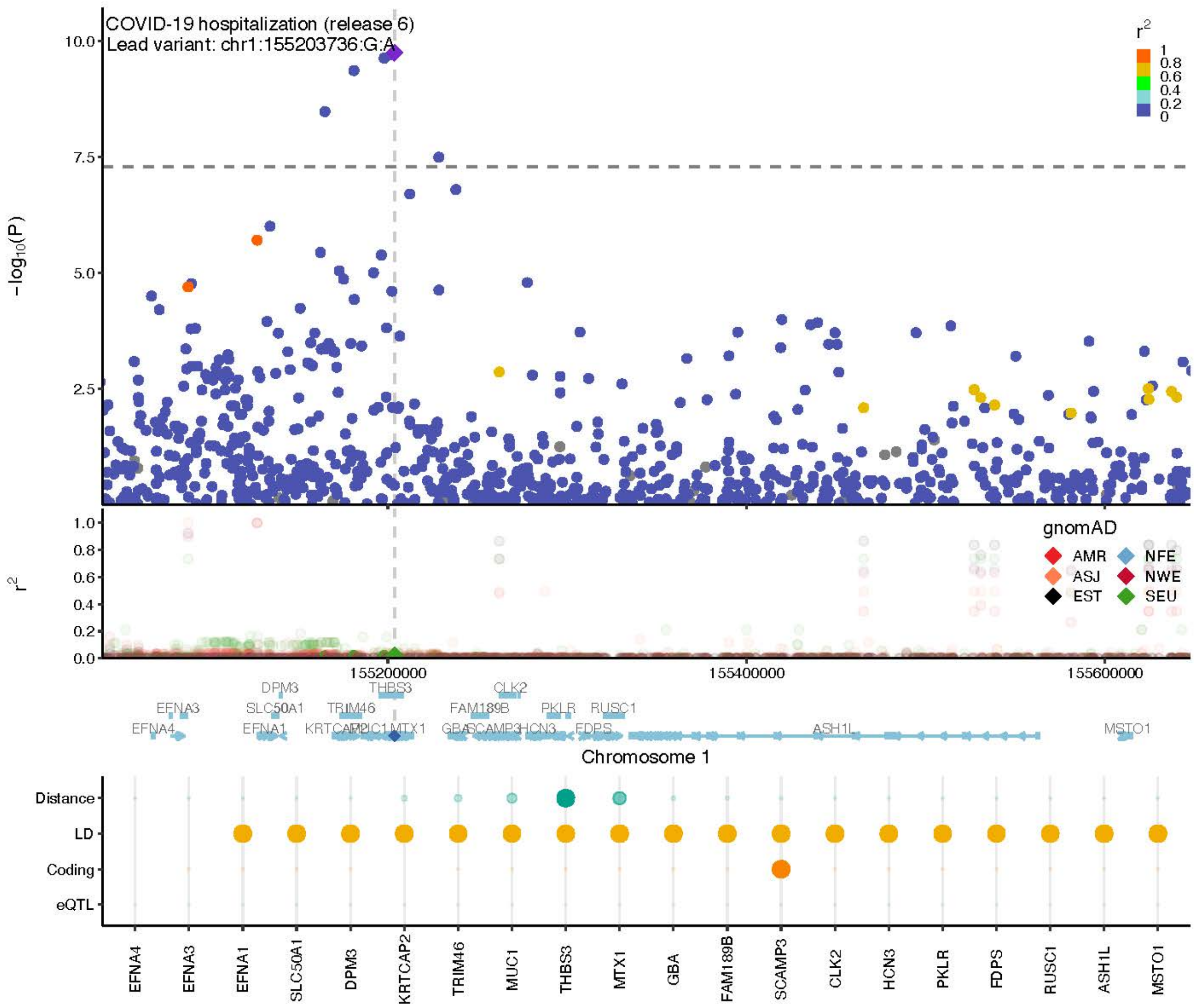
COVID-19 hospitalization (release 6)
Lead variant: chr1:155203736:G:A
−log10(P)
r2
gnomAD
AMR
ASJ
EST
NFE
NWE
SEU
155200000
155400000
155600000
Chromosome 1
Distance
LD
Coding
eQTL
EFNA4
EFNA3
EFNA1
SLC50A1
DPM3
KRTCAP2
TRIM46
MUC1
THBS3
MTX1
GBA
FAM189B
SCAMP3
CLK2
HCN3
PKLR
FDPS
RUSC1
ASH1L
MSTO1

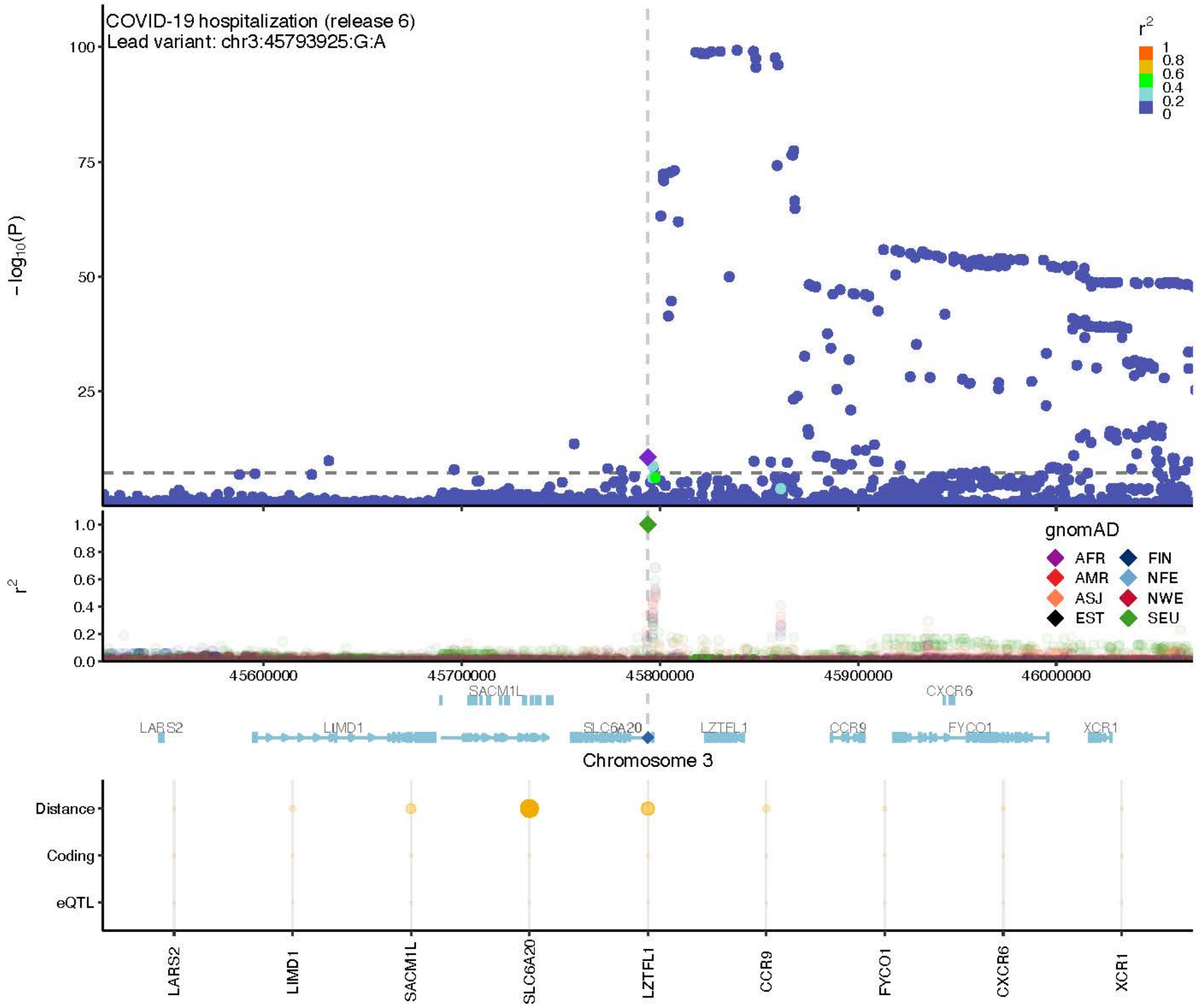
COVID-19 hospitalization (release 6)
Lead variant: chr3:45793925:G:A
-log10(P)
r2
1
0.8
0.6
0.4
0.2
0
gnomAD
AFR
AMR
ASJ
EST
FIN
NFE
NWE
SEU
45600000
45700000
45800000
45900000
46000000
Chromosome 3
Distance
Coding
eQTL
LARS2
LIMD1
SACM1L
SLC6A20
LZTFL1
CCR9
FYCO1
CXCR6
XCR1

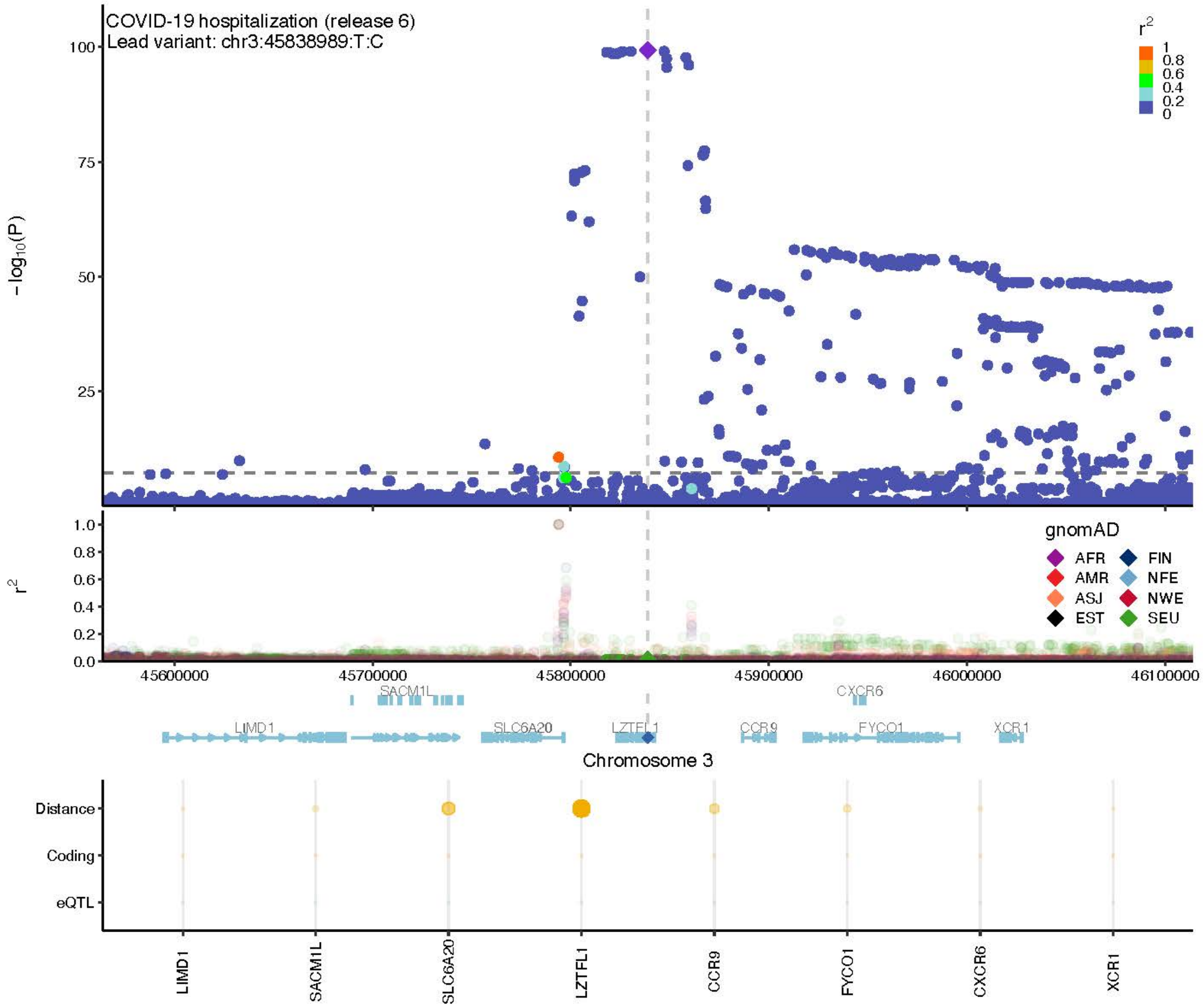
COVID-19 hospitalization (release 6)
Lead variant: chr3:45838989:T:C
r2
1
0.8
0.6
0.4
0.2
0
-log10(P)
100
75
50
25
r2
1.0
0.8
0.6
0.4
0.2
0.0
gnomAD
AFR
AMR
ASJ
EST
FIN
NFE
NWE
SEU
45600000
45700000
45800000
45900000
46000000
46100000
SACM1L
CXCR6
LIMD1
SLC6A20
LZTFL1
CCR9
FYCO1
XCR1
Chromosome 3
Distance
Coding
eQTL
LIMD1
SACM1L
SLC6A20
LZTFL1
CCR9
FYCO1
CXCR6
XCR1

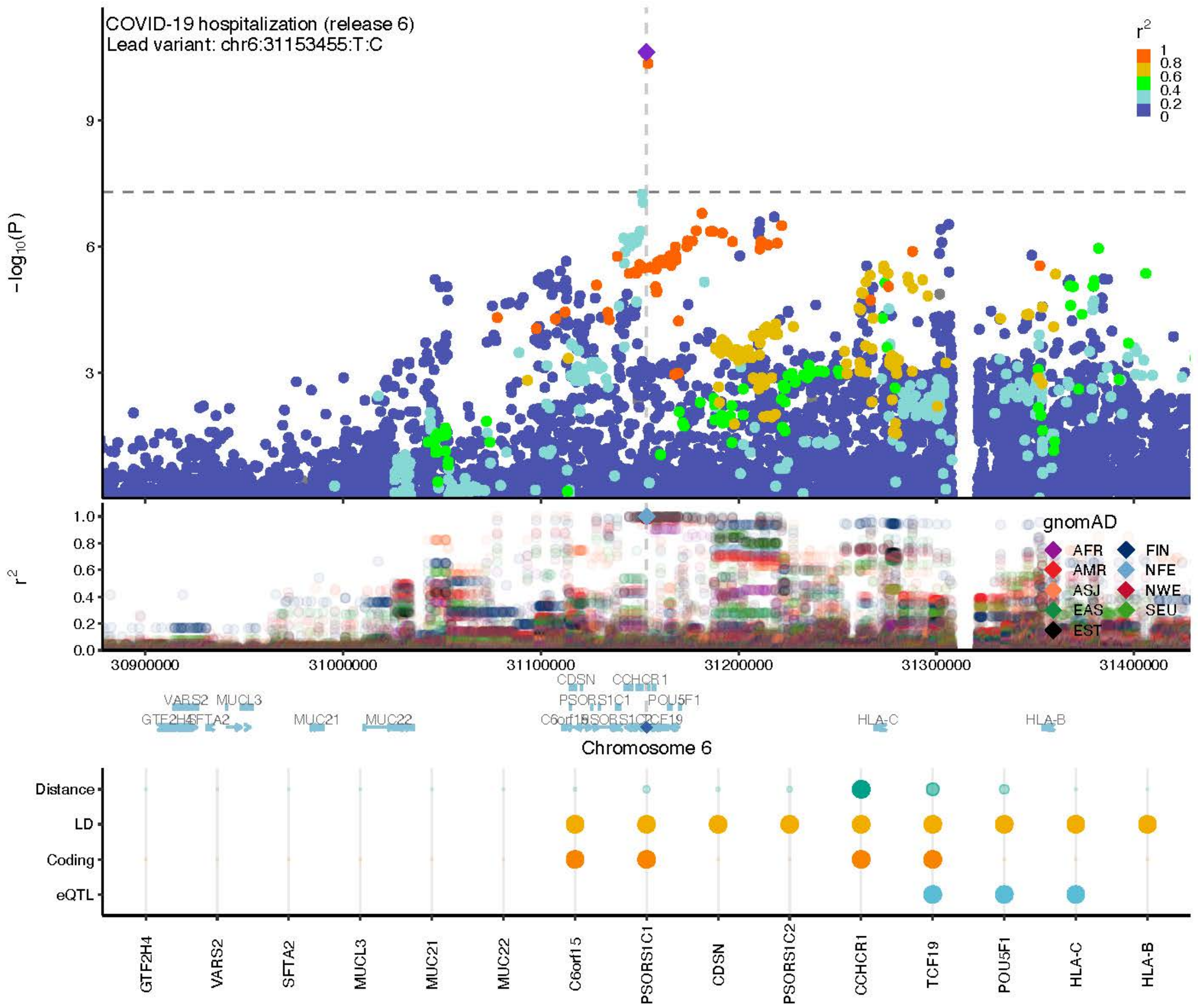
COVID-19 hospitalization (release 6)
Lead variant: chr6:31153455:T:C
r^2
1
0.8
0.6
0.4
0.2
0
$-\log_{10}(P)$
9
6
3
r^2
1.0
0.8
0.6
0.4
0.2
0.0
gnomAD
AFR
AMR
ASJ
EAS
EST
FIN
NFE
NWE
SEU
30900000
31000000
31100000
31200000
31300000
31400000
Chromosome 6
Distance
LD
Coding
eQTL
GTF2H4
VARS2
SFTA2
MUCL3
MUC21
MUC22
C6orf15
PSORS1C1
CDSN
PSORS1C2
CCHCR1
TCF19
POU5F1
HLA-C
HLA-B

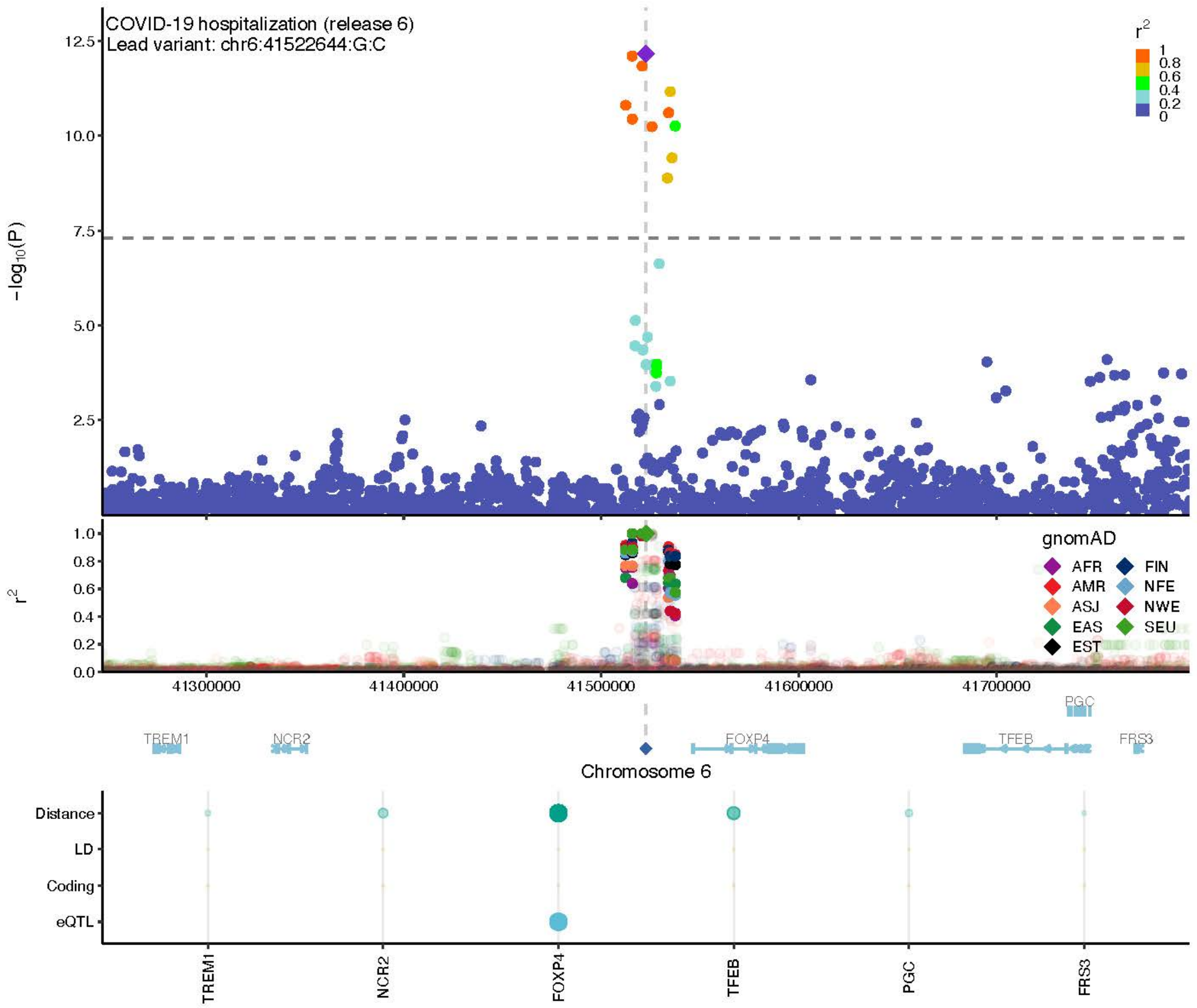
COVID-19 hospitalization (release 6)
Lead variant: chr6:41522644:G:C
-log10(P)
12.5
10.0
7.5
5.0
2.5
r2
1
0.8
0.6
0.4
0.2
0
1.0
0.8
0.6
0.4
0.2
0.0
gnomAD
AFR
AMR
ASJ
EAS
EST
FIN
NFE
NWE
SEU
41300000
41400000
41500000
41600000
41700000
PGC
TREM1
NCR2
FOXP4
TFEB
FRS3
Chromosome 6
Distance
LD
Coding
eQTL
TREM1
NCR2
FOXP4
TFEB
PGC
FRS3

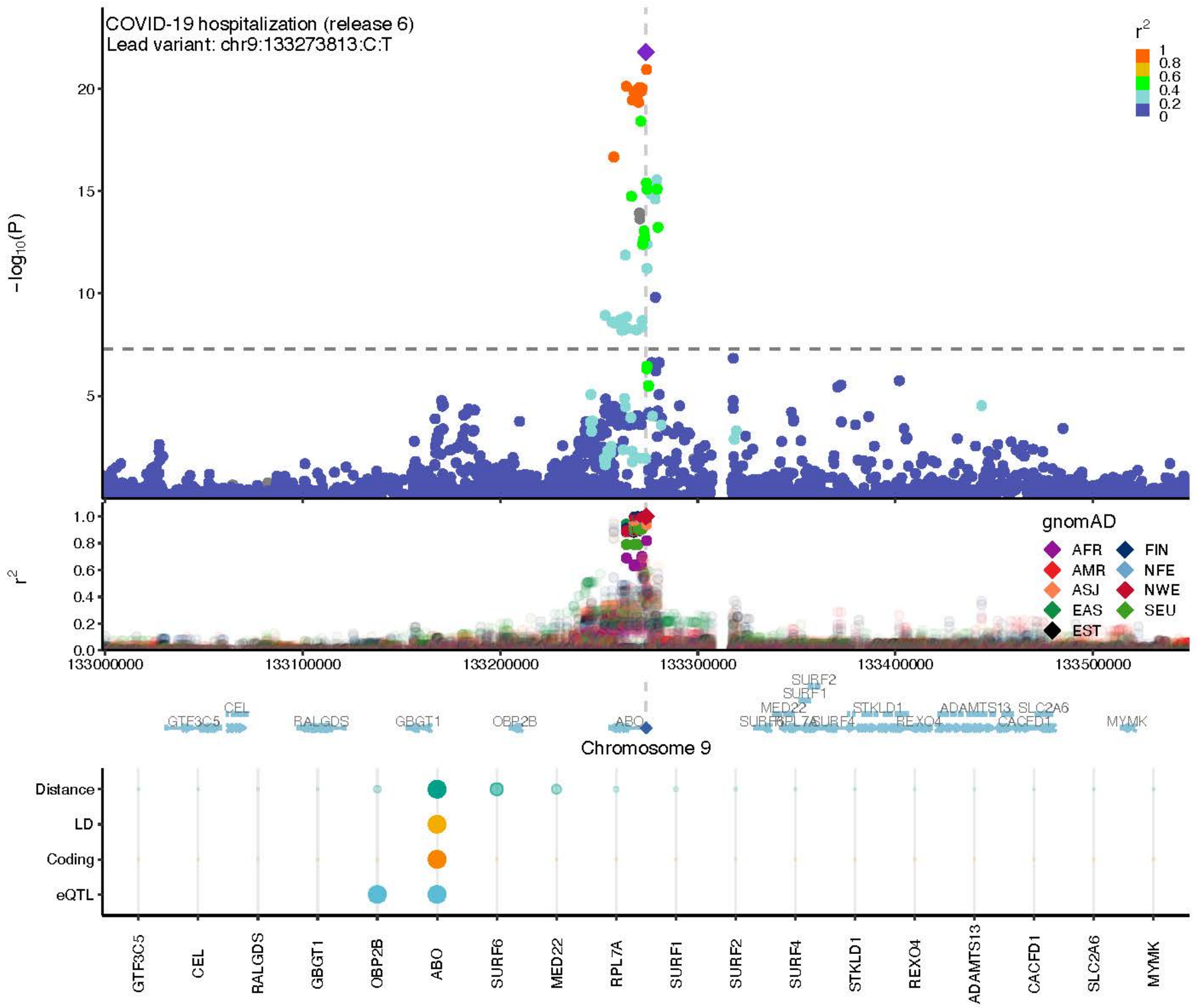
COVID-19 hospitalization (release 6)
Lead variant: chr9:133273813:C:T
-log10(P)
r2
gnomAD
AFR
AMR
ASJ
EAS
EST
FIN
NFE
NWE
SEU
133000000
133100000
133200000
133300000
133400000
133500000
Chromosome 9
Distance
LD
Coding
eQTL
GTF3C5
CEL
RALGDS
GBGT1
OBP2B
ABO
SURF6
MED22
RPL7A
SURF1
SURF2
SURF4
STKLD1
REXO4
ADAMTS13
CACFD1
SLC2A6
MYMK

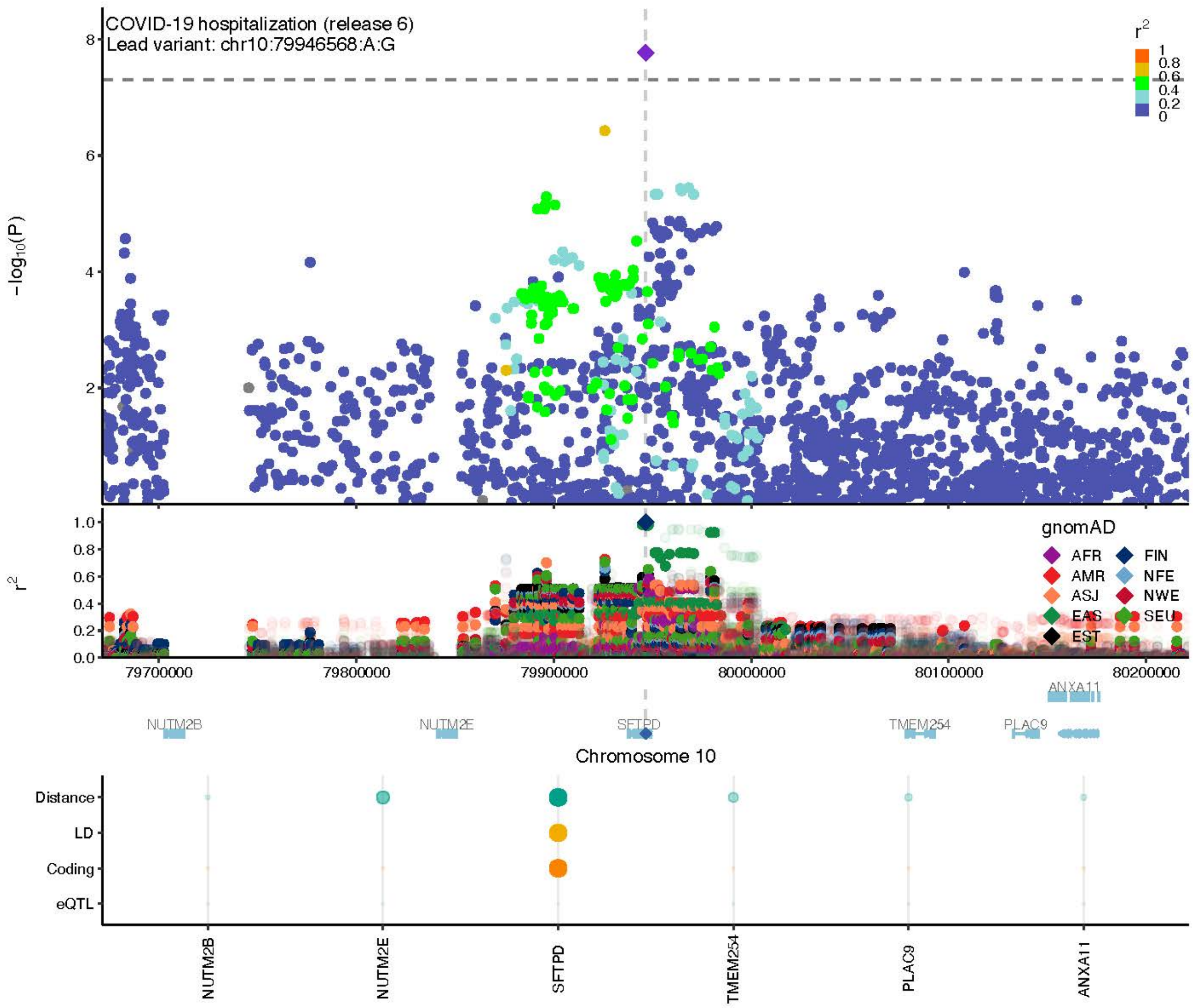
COVID-19 hospitalization (release 6)
Lead variant: chr10:79946568:A:G
−log10(P)
r2
1
0.8
0.6
0.4
0.2
0
gnomAD
AFR
AMR
ASJ
EAS
EST
FIN
NFE
NWE
SEU
79700000
79800000
79900000
80000000
80100000
80200000
ANXA11
NUTM2B
NUTM2E
SFTPD
TMEM254
PLAC9
Chromosome 10
Distance
LD
Coding
eQTL

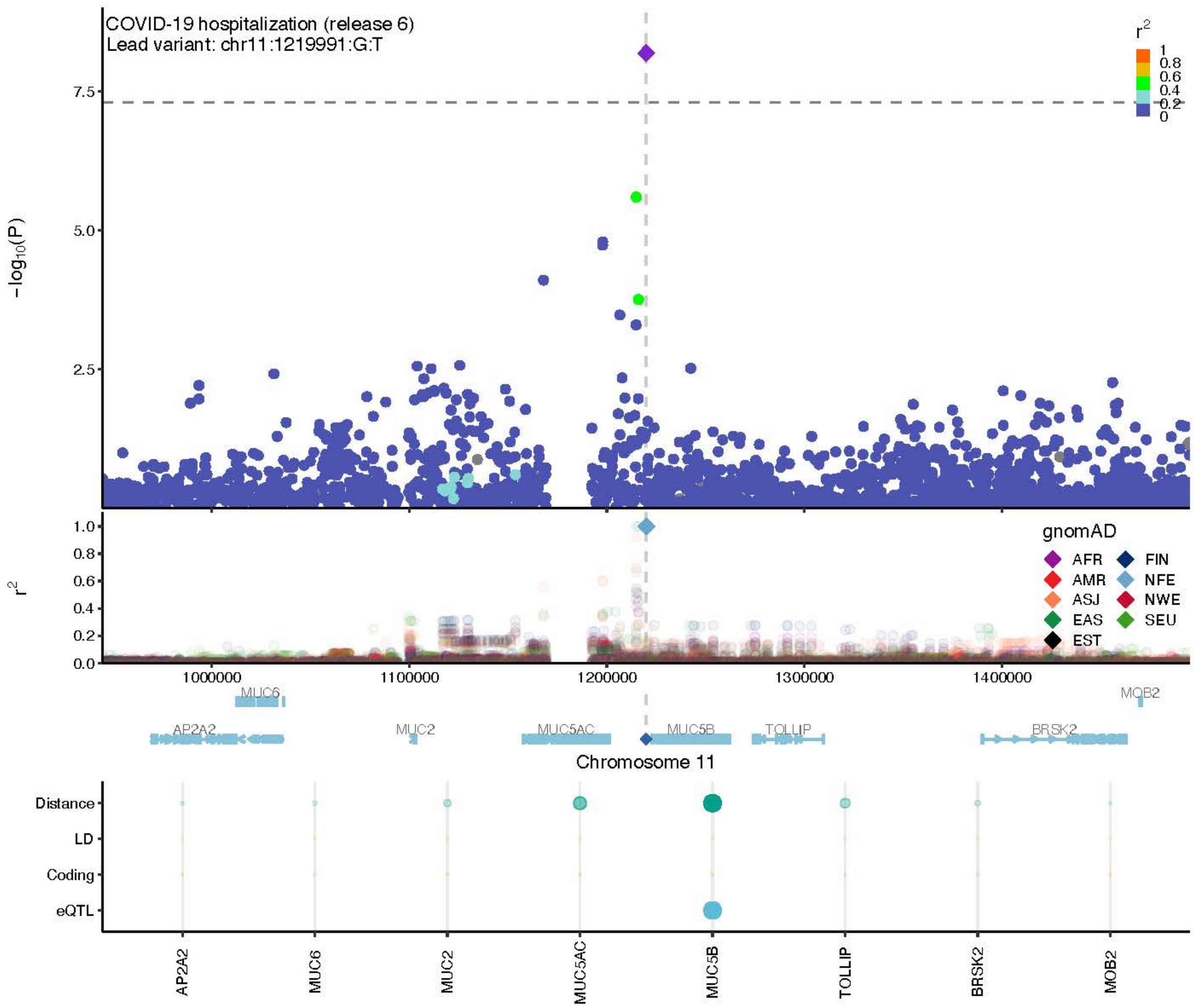
COVID-19 hospitalization (release 6)
Lead variant: chr11:1219991:G:T
−log10(P)
7.5
5.0
2.5
r2
1
0.8
0.6
0.4
0.2
0
1.0
0.8
0.6
0.4
0.2
0.0
gnomAD
AFR
AMR
ASJ
EAS
EST
FIN
NFE
NWE
SEU
1000000
1100000
1200000
1300000
1400000
MUC6
MOB2
AP2A2
MUC2
MUC5AC
MUC5B
TOLLIP
BRSK2
Chromosome 11
Distance
LD
Coding
eQTL
AP2A2
MUC6
MUC2
MUC5AC
MUC5B
TOLLIP
BRSK2
MOB2

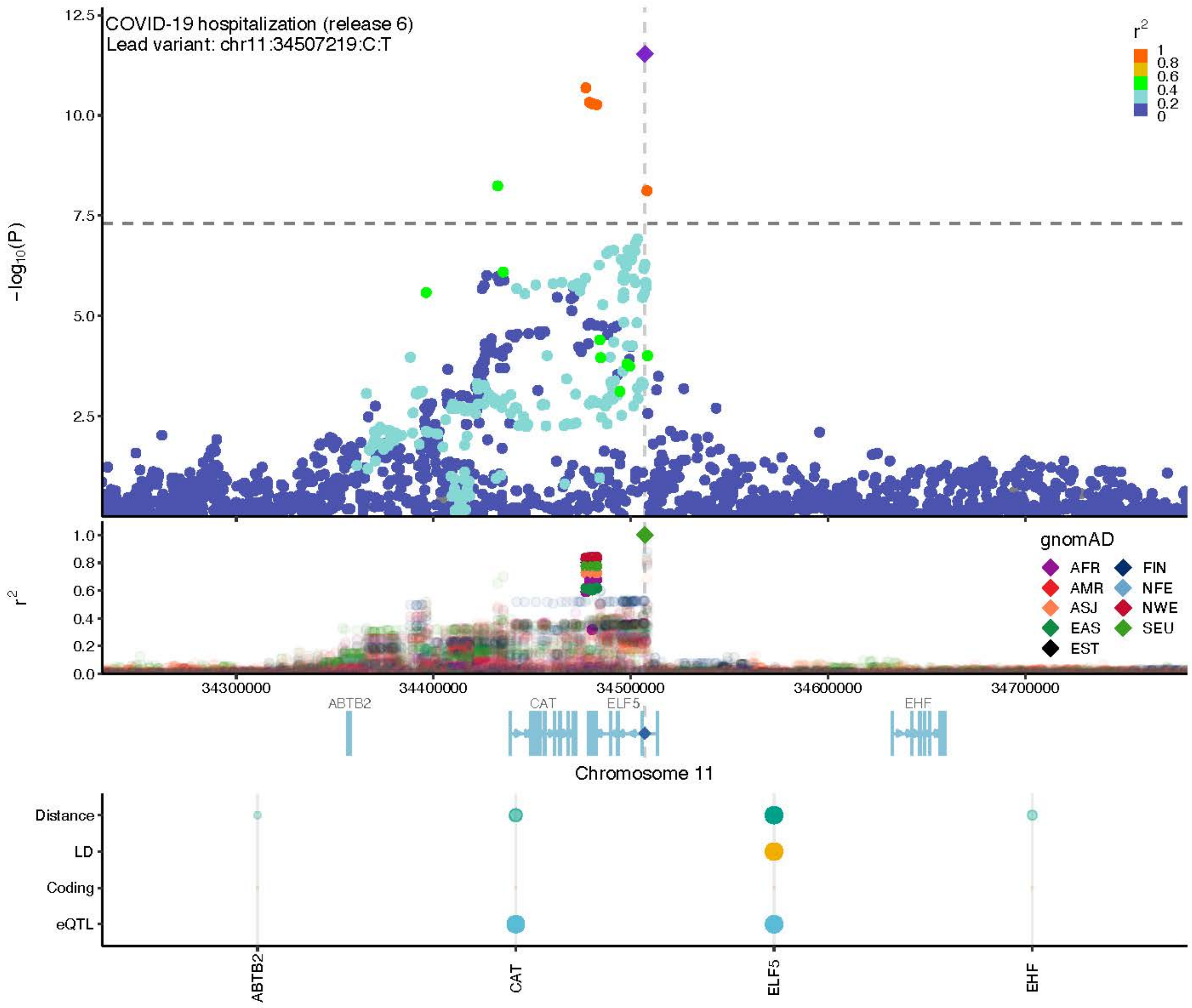
COVID-19 hospitalization (release 6)
Lead variant: chr11:34507219:C:T
-log10(P)
12.5
10.0
7.5
5.0
2.5
r2
1
0.8
0.6
0.4
0.2
0
1.0
0.8
0.6
0.4
0.2
0.0
gnomAD
AFR
AMR
ASJ
EAS
EST
FIN
NFE
NWE
SEU
34300000
34400000
34500000
34600000
34700000
ABTB2
CAT
ELF5
EHF
Chromosome 11
Distance
LD
Coding
eQTL
ABTB2
CAT
ELF5
EHF

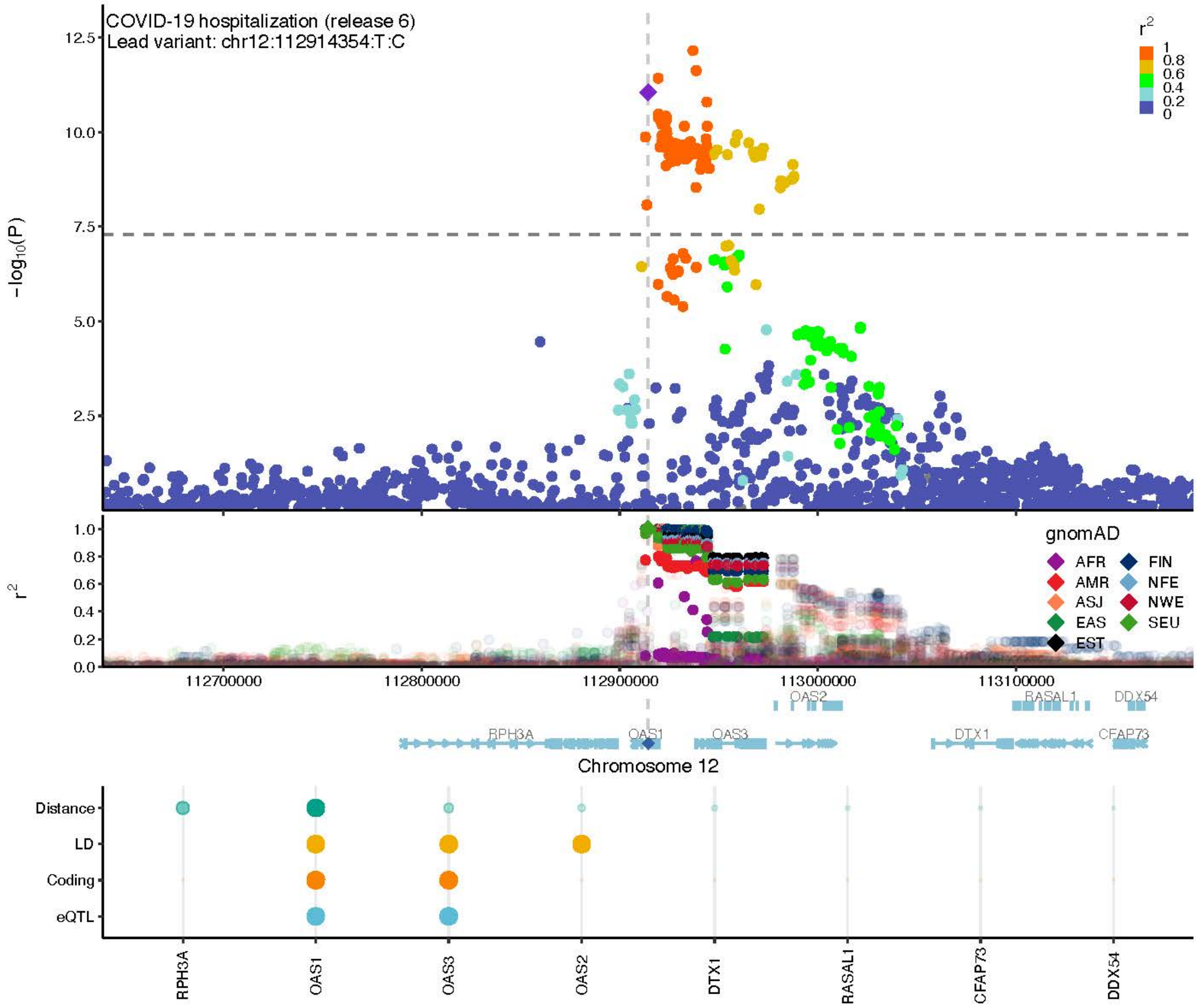
COVID-19 hospitalization (release 6)
Lead variant: chr12:112914354:T:C
-log10(P)
12.5
10.0
7.5
5.0
2.5
r2
1
0.8
0.6
0.4
0.2
0
gnomAD
AFR
AMR
ASJ
EAS
EST
FIN
NFE
NWE
SEU
1.0
0.8
0.6
0.4
0.2
0.0
112700000
112800000
112900000
113000000
113100000
OAS2
RASAL1
DDX54
RPH3A
OAS1
OAS3
DTX1
CFAP73
Chromosome 12
Distance
LD
Coding
eQTL
RPH3A
OAS1
OAS3
OAS2
DTX1
RASAL1
CFAP73
DDX54

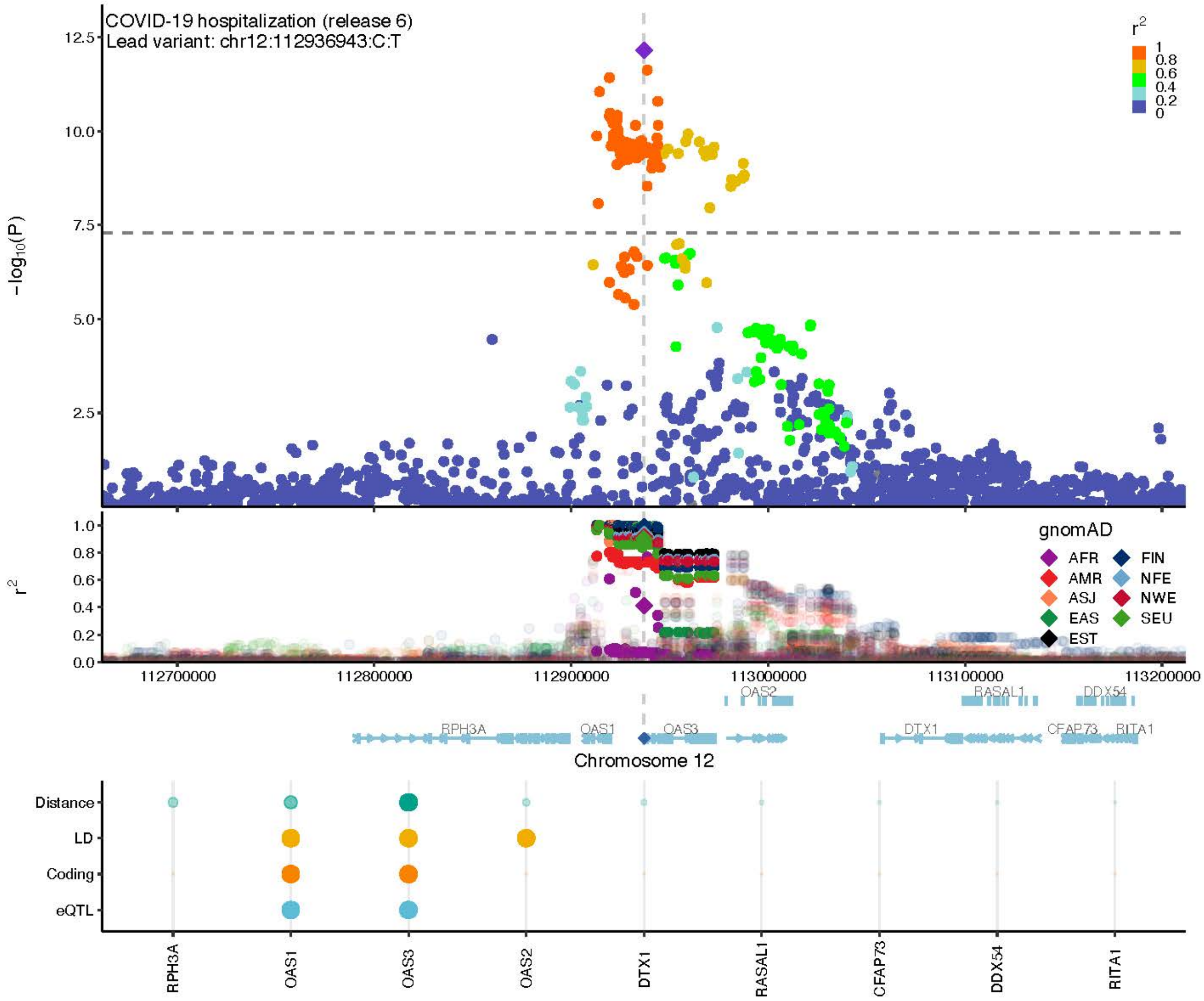
COVID-19 hospitalization (release 6)
Lead variant: chr12:112936943:C:T
-log10(P)
12.5
10.0
7.5
5.0
2.5
r2
1
0.8
0.6
0.4
0.2
0
1.0
0.8
0.6
0.4
0.2
0.0
gnomAD
AFR
AMR
ASJ
EAS
EST
FIN
NFE
NWE
SEU
112700000
112800000
112900000
113000000
113100000
113200000
OAS2
RASAL1
DDX54
RPH3A
OAS1
OAS3
DTX1
CFAP73
RITA1
Chromosome 12
Distance
LD
Coding
eQTL
RPH3A
OAS1
OAS3
OAS2
DTX1
RASAL1
CFAP73
DDX54
RITA1

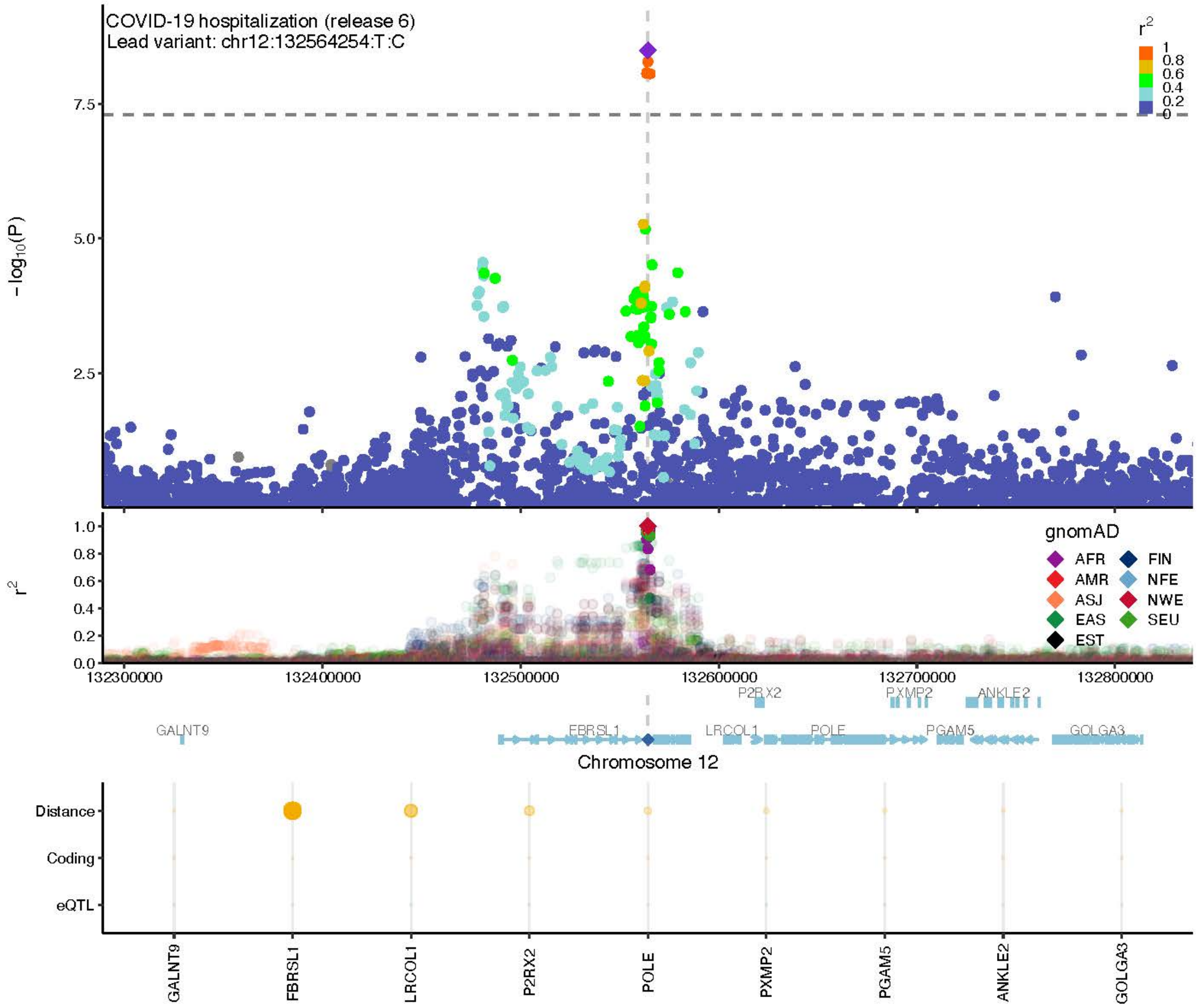
COVID-19 hospitalization (release 6)
Lead variant: chr12:132564254:T:C
−log10(P)
7.5
5.0
2.5
r2
1
0.8
0.6
0.4
0.2
0
r2
1.0
0.8
0.6
0.4
0.2
0.0
gnomAD
AFR
AMR
ASJ
EAS
EST
FIN
NFE
NWE
SEU
132300000
132400000
132500000
132600000
132700000
132800000
P2RX2
PXMP2
ANKLE2
GALNT9
FBRSL1
LRCOL1
POLE
PGAM5
GOLGA3
Chromosome 12
Distance
Coding
eQTL
GALNT9
FBRSL1
LRCOL1
P2RX2
POLE
PXMP2
PGAM5
ANKLE2
GOLGA3

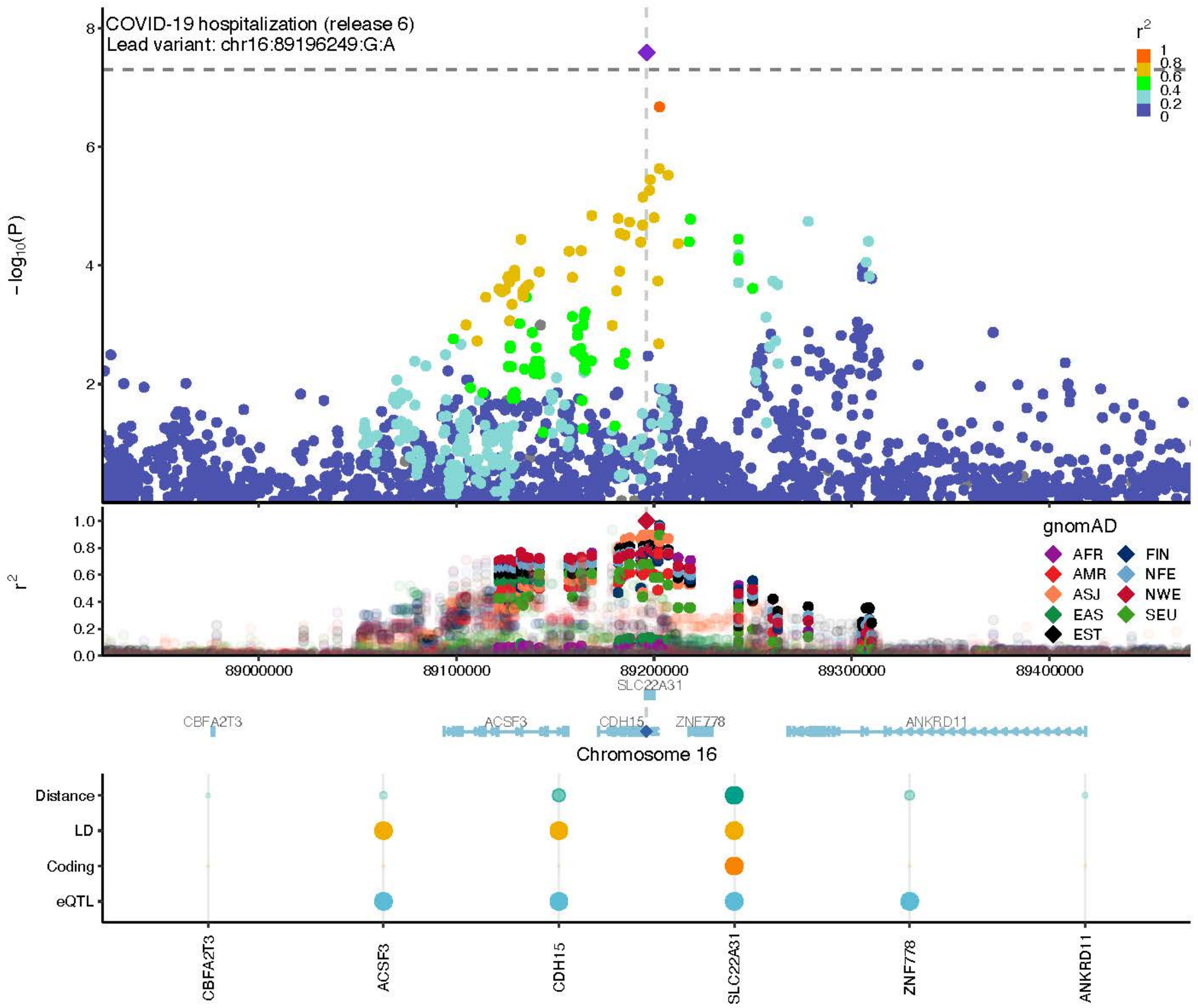
COVID-19 hospitalization (release 6)
Lead variant: chr16:89196249:G:A
−log10(P)
r2
1
0.8
0.6
0.4
0.2
0
gnomAD
AFR
AMR
ASJ
EAS
EST
FIN
NFE
NWE
SEU
89000000
89100000
89200000
89300000
89400000
SLC22A31
CBFA2T3
ACSF3
CDH15
ZNF778
ANKRD11
Chromosome 16
Distance
LD
Coding
eQTL
CBFA2T3
ACSF3
CDH15
SLC22A31
ZNF778
ANKRD11

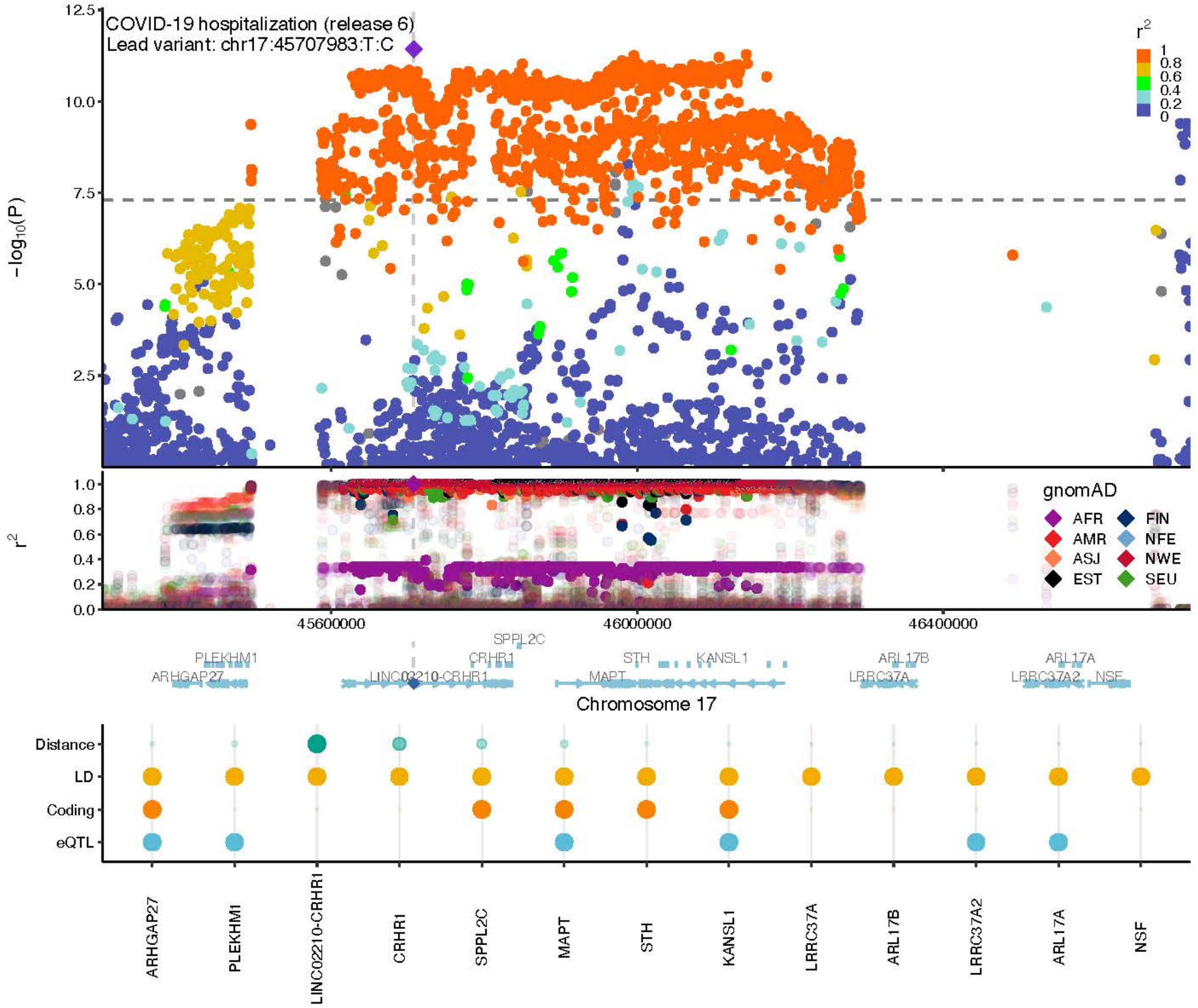
COVID-19 hospitalization (release 6)
Lead variant: chr17:45707983:T:C
-log10(P)
r2
gnomAD
AFR
FIN
AMR
NFE
ASJ
NWE
EST
SEU
45600000
46000000
46400000
Chromosome 17
Distance
LD
Coding
eQTL
ARHGAP27
PLEKHM1
LINC02210-CRHR1
CRHR1
SPPL2C
MAPT
STH
KANSL1
LRRC37A
ARL17B
LRRC37A2
ARL17A
NSF

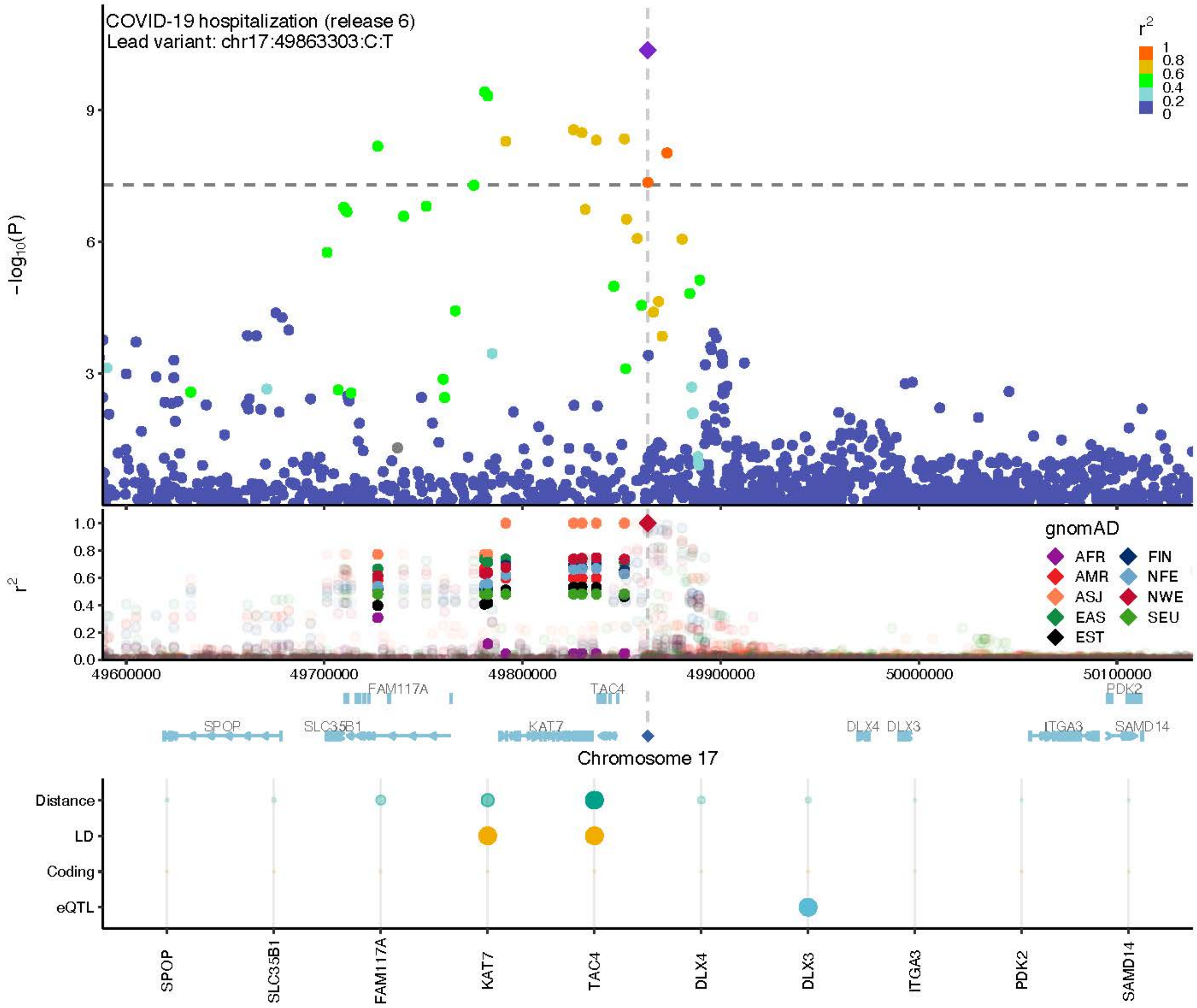
COVID-19 hospitalization (release 6)
Lead variant: chr17:49863303:C:T
−log10(P)
r2
gnomAD
AFR FIN AMR NFE ASJ NWE EAS SEU EST
49600000 49700000 49800000 49900000 50000000 50100000
FAM117A TAC4 PDK2 SPOP SLC35B1 KAT7 DLX4 DLX3 ITGA3 SAMD14
Chromosome 17
Distance
LD
Coding
eQTL
SPOP SLC35B1 FAM117A KAT7 TAC4 DLX4 DLX3 ITGA3 PDK2 SAMD14

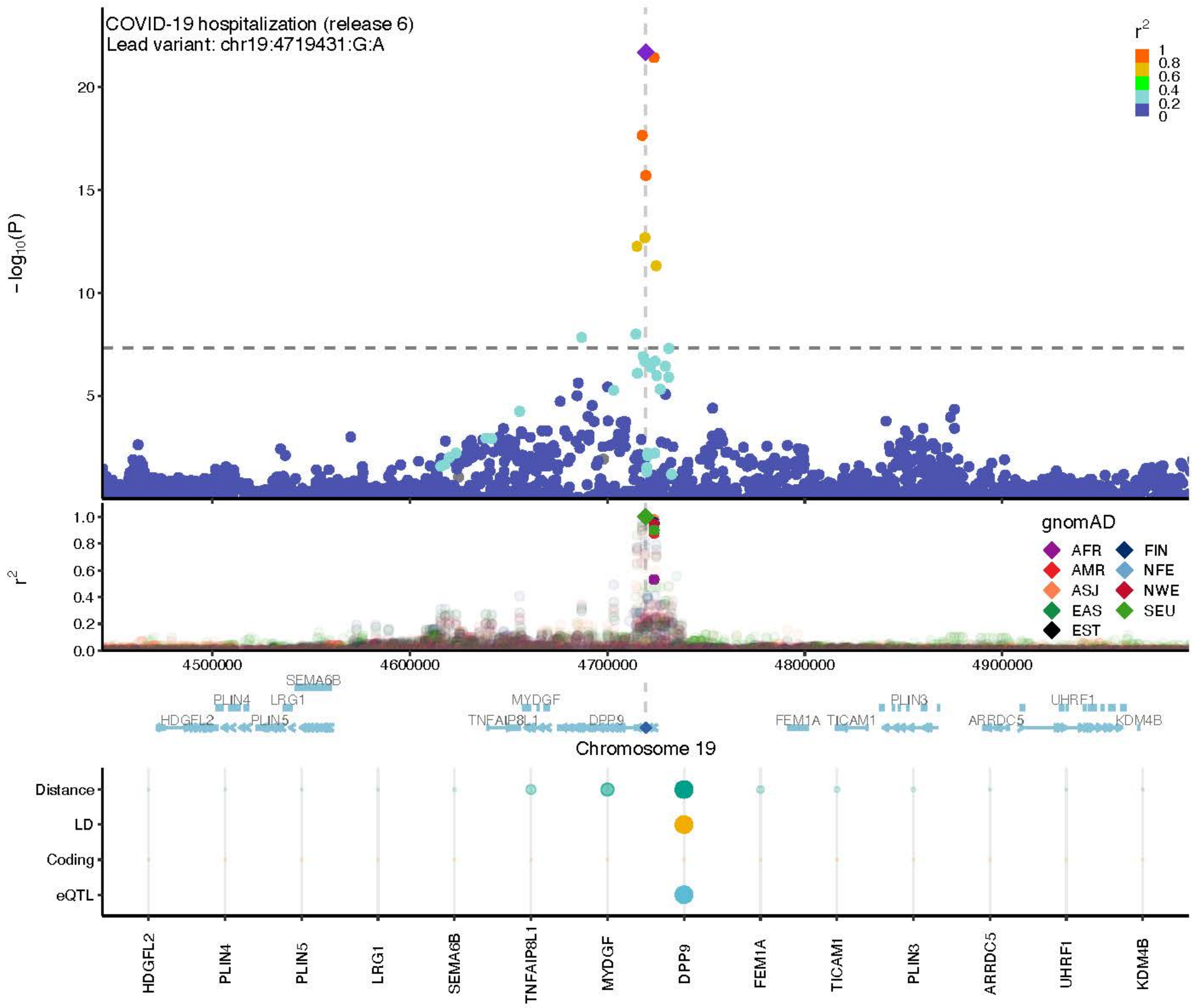
COVID-19 hospitalization (release 6)
Lead variant: chr19:4719431:G:A
-log10(P)
r2
gnomAD
AFR
AMR
ASJ
EAS
EST
FIN
NFE
NWE
SEU
Chromosome 19
Distance
LD
Coding
eQTL
HDGFL2
PLIN4
PLIN5
LRG1
SEMA6B
TNFAIP8L1
MYDGF
DPP9
FEM1A
TICAM1
PLIN3
ARRDC5
UHRF1
KDM4B

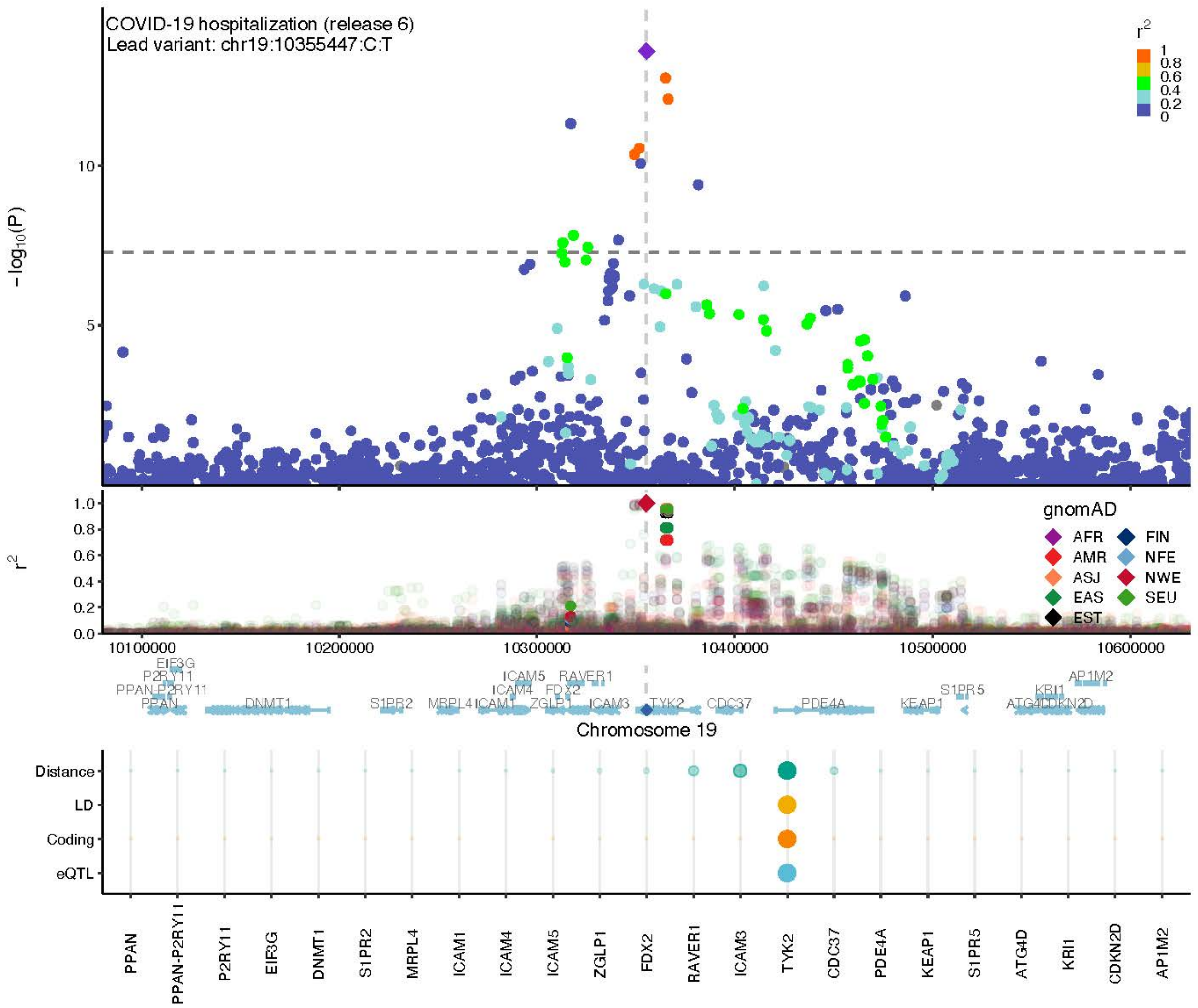
COVID-19 hospitalization (release 6)
Lead variant: chr19:10355447:C:T
$-\log_{10}(P)$
r^2
gnomAD
AFR
AMR
ASJ
EAS
EST
FIN
NFE
NWE
SEU
10100000
10200000
10300000
10400000
10500000
10600000
Chromosome 19
Distance
LD
Coding
eQTL
PPAN
PPAN-P2RY11
P2RY11
EIF3G
DNMT1
S1PR2
MRPL4
ICAM1
ICAM4
ICAM5
ZGLP1
FDX2
RAVER1
ICAM3
TYK2
CDC37
PDE4A
KEAP1
S1PR5
ATG4D
KRI1
CDKN2D
AP1M2

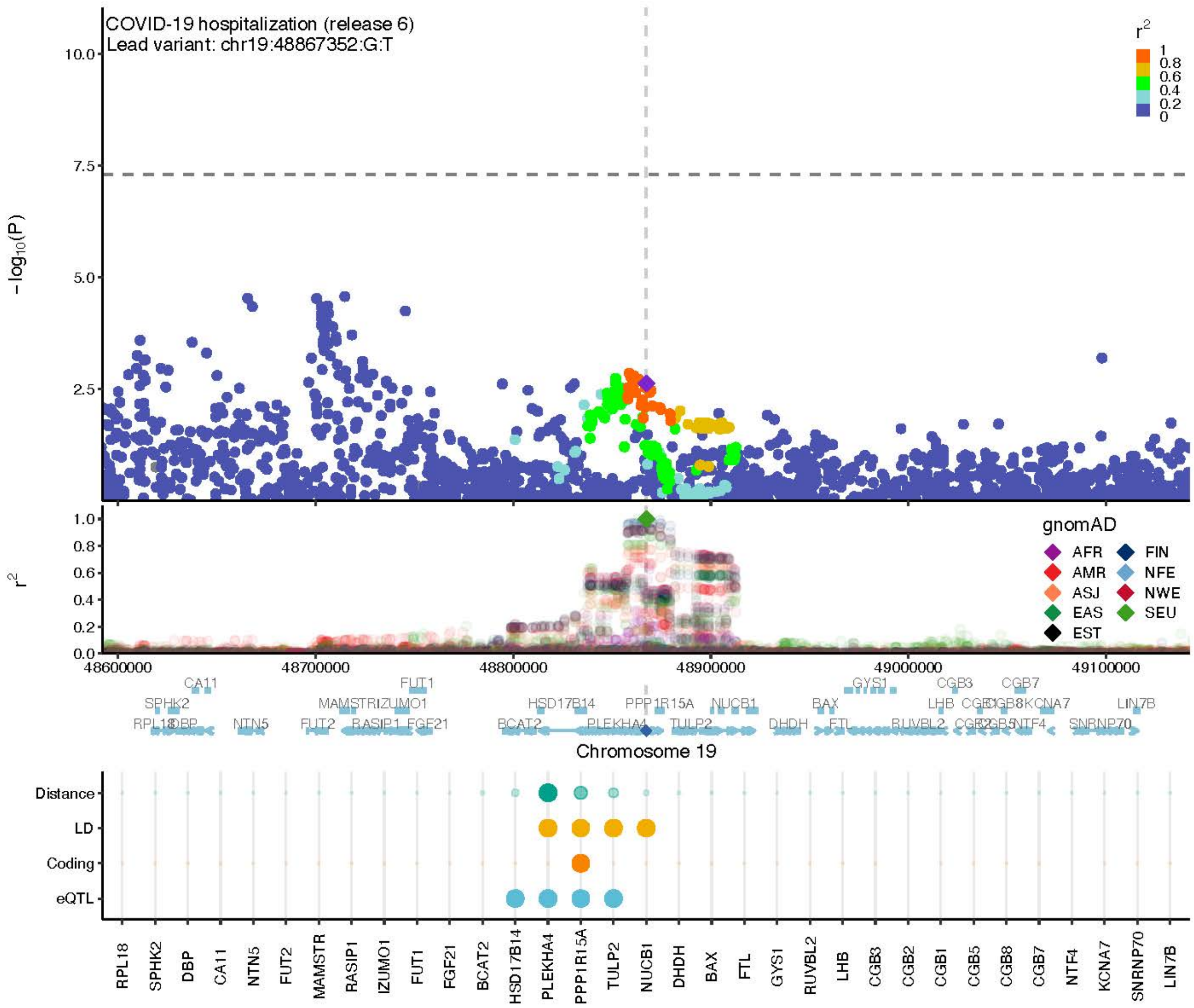
COVID-19 hospitalization (release 6)
Lead variant: chr19:48867352:G:T
−log10(P)
r2
gnomAD
AFR FIN AMR NFE ASJ NWE EAS SEU EST
Chromosome 19
48600000 48700000 48800000 48900000 49000000 49100000
Distance
LD
Coding
eQTL
RPL18 SPHK2 DBP CA11 NTN5 FUT2 MAMSTR RASIP1 IZUMO1 FUT1 FGF21 BCAT2 HSD17B14 PLEKHA4 PPP1R15A TULP2 NUCB1 DHDH BAX FTL GYS1 RUVBL2 LHB CGB3 CGB2 CGB1 CGB5 CGB8 CGB7 NTF4 KCNA7 SNRNP70 LIN7B

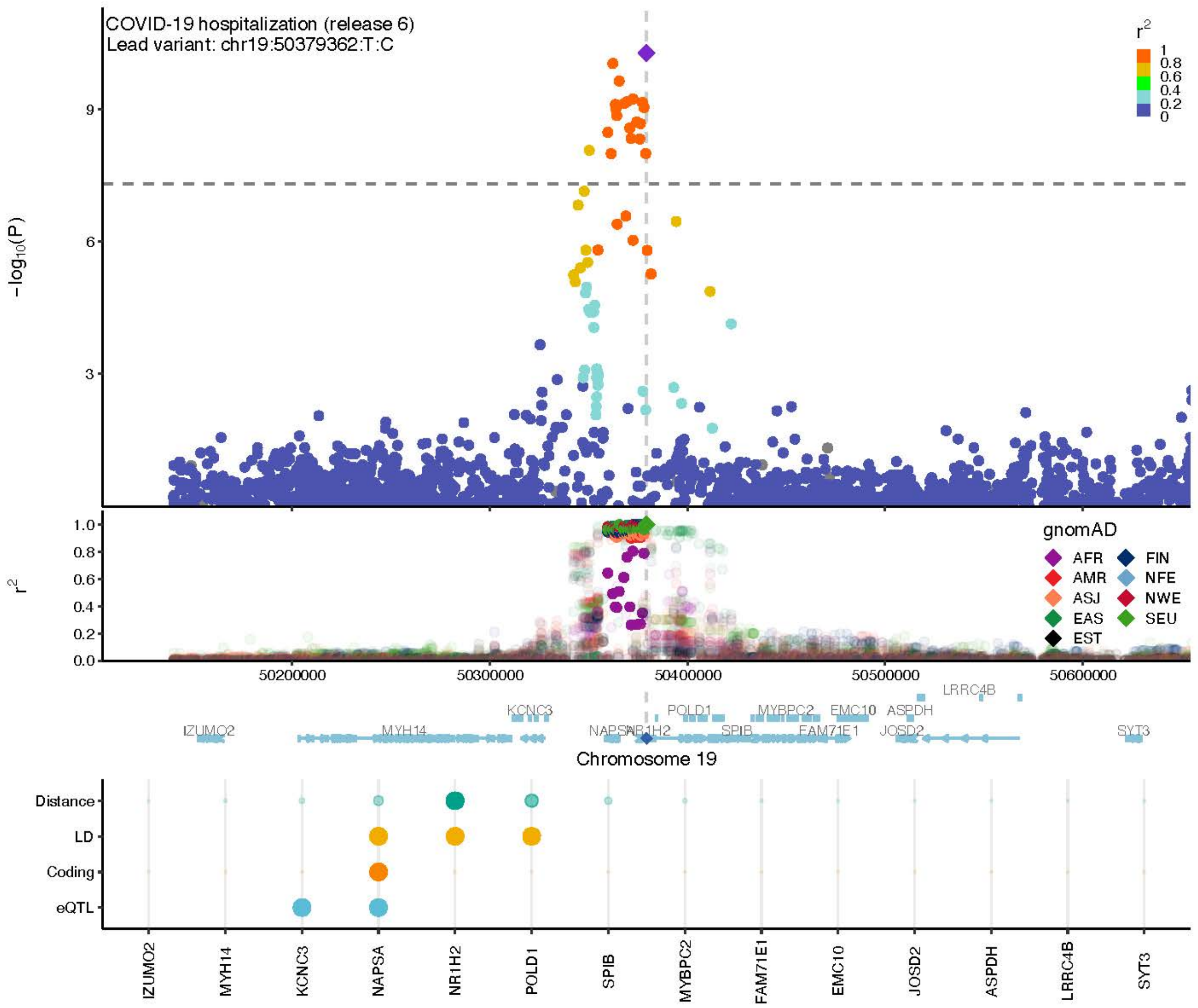
COVID-19 hospitalization (release 6)
Lead variant: chr19:50379362:T:C
-log10(P)
r2
1
0.8
0.6
0.4
0.2
0
gnomAD
AFR
AMR
ASJ
EAS
EST
FIN
NFE
NWE
SEU
50200000
50300000
50400000
50500000
50600000
Chromosome 19
Distance
LD
Coding
eQTL
IZUMO2
MYH14
KCNC3
NAPSA
NR1H2
POLD1
SPIB
MYBPC2
FAM71E1
EMC10
JOSD2
ASPDH
LRRC4B
SYT3

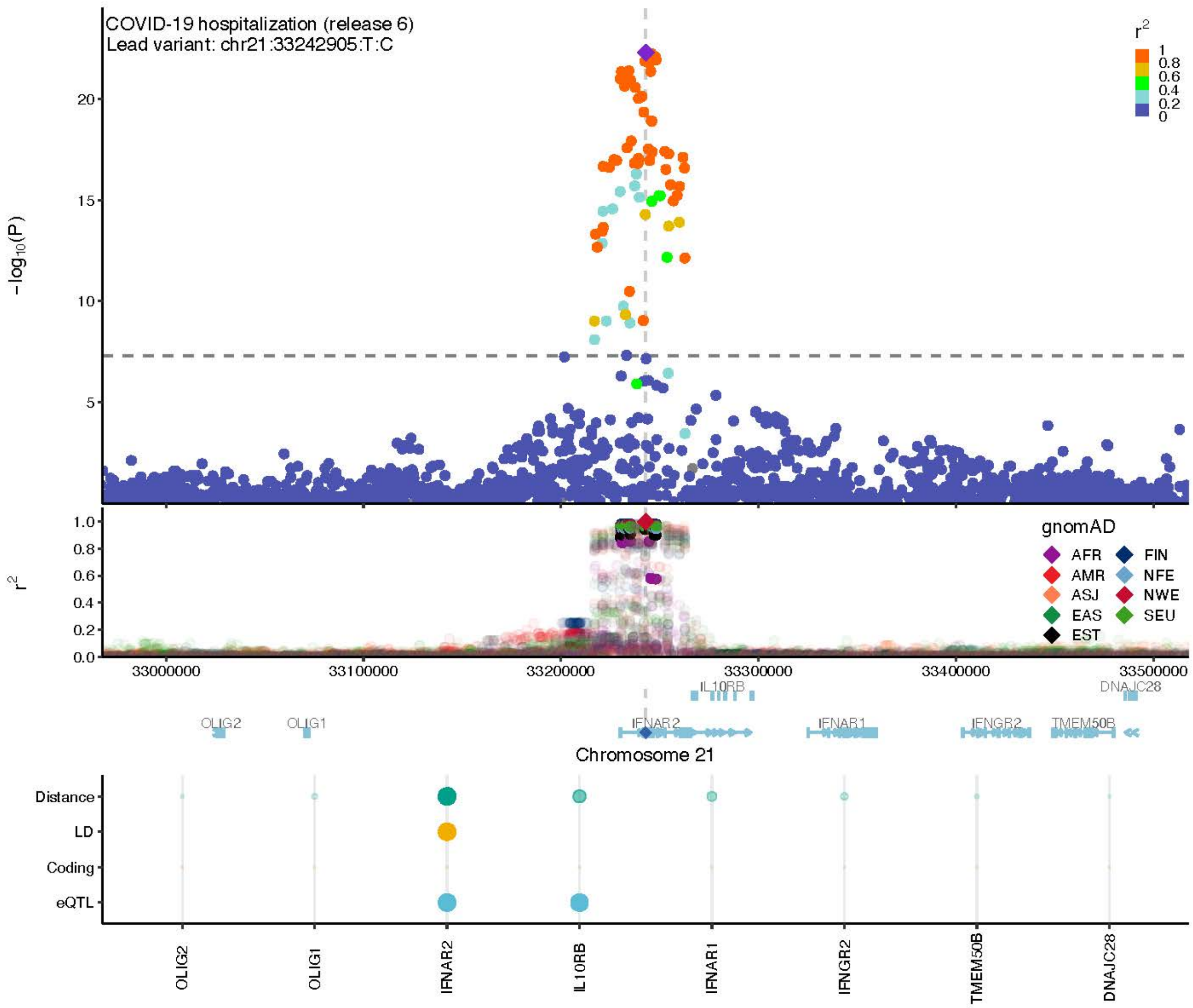
COVID-19 hospitalization (release 6)
Lead variant: chr21:33242905:T:C
$-\log_{10}(P)$
r^2
gnomAD
AFR
AMR
ASJ
EAS
EST
FIN
NFE
NWE
SEU
33000000
33100000
33200000
33300000
33400000
33500000
Chromosome 21
IL10RB
DNAJC28
OLIG2
OLIG1
IFNAR2
IFNAR1
IFNGR2
TMEM50B
Distance
LD
Coding
eQTL
OLIG2
OLIG1
IFNAR2
IL10RB
IFNAR1
IFNGR2
TMEM50B
DNAJC28

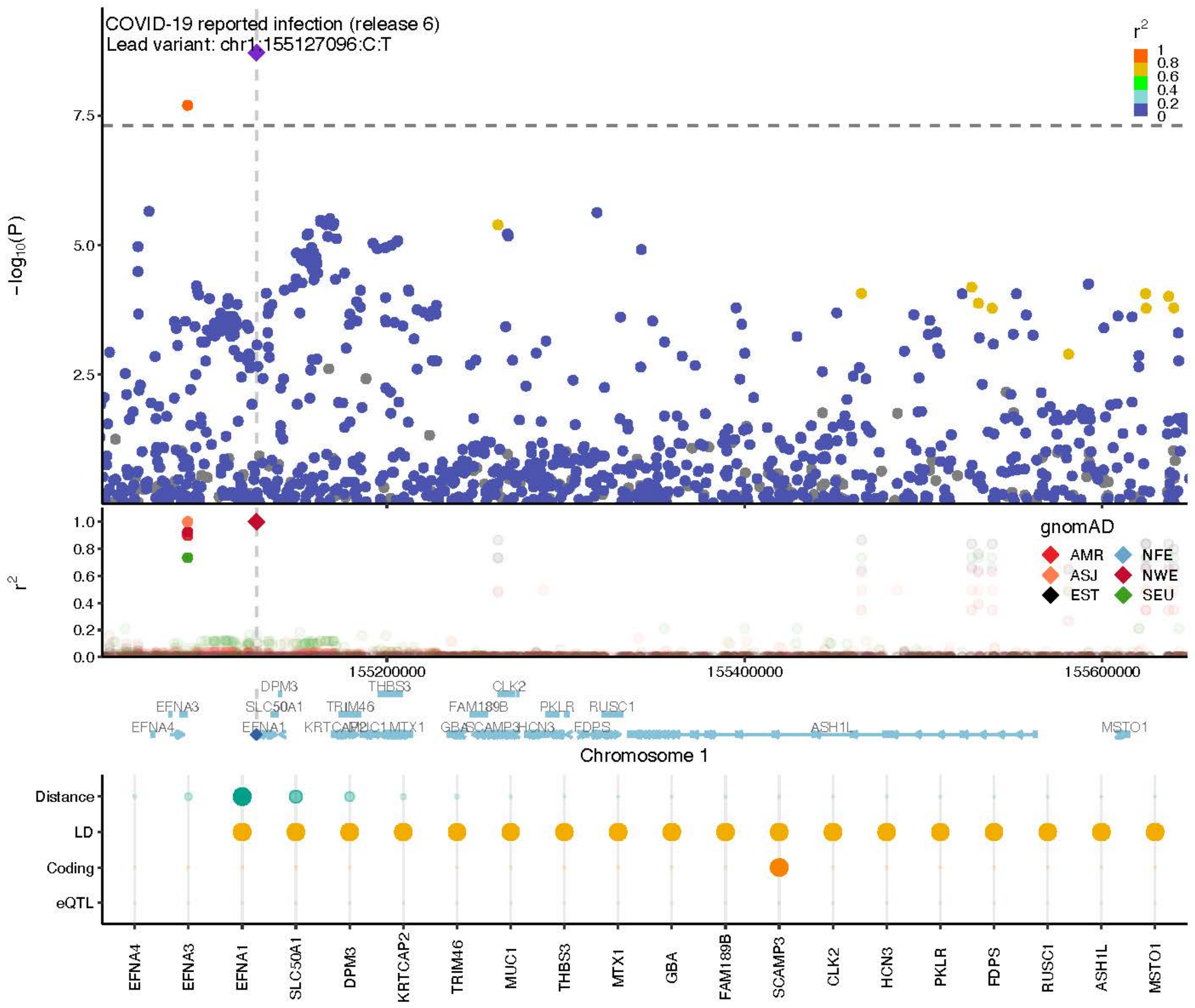
COVID-19 reported infection (release 6)
Lead variant: chr1:155127096:C:T
−log10(P)
r2
gnomAD
AMR
ASJ
EST
NFE
NWE
SEU
155200000
155400000
155600000
Chromosome 1
Distance
LD
Coding
eQTL
EFNA4
EFNA3
EFNA1
SLC50A1
DPM3
KRTCAP2
TRIM46
MUC1
THBS3
MTX1
GBA
FAM189B
SCAMP3
CLK2
HCN3
PKLR
FDPS
RUSC1
ASH1L
MSTO1

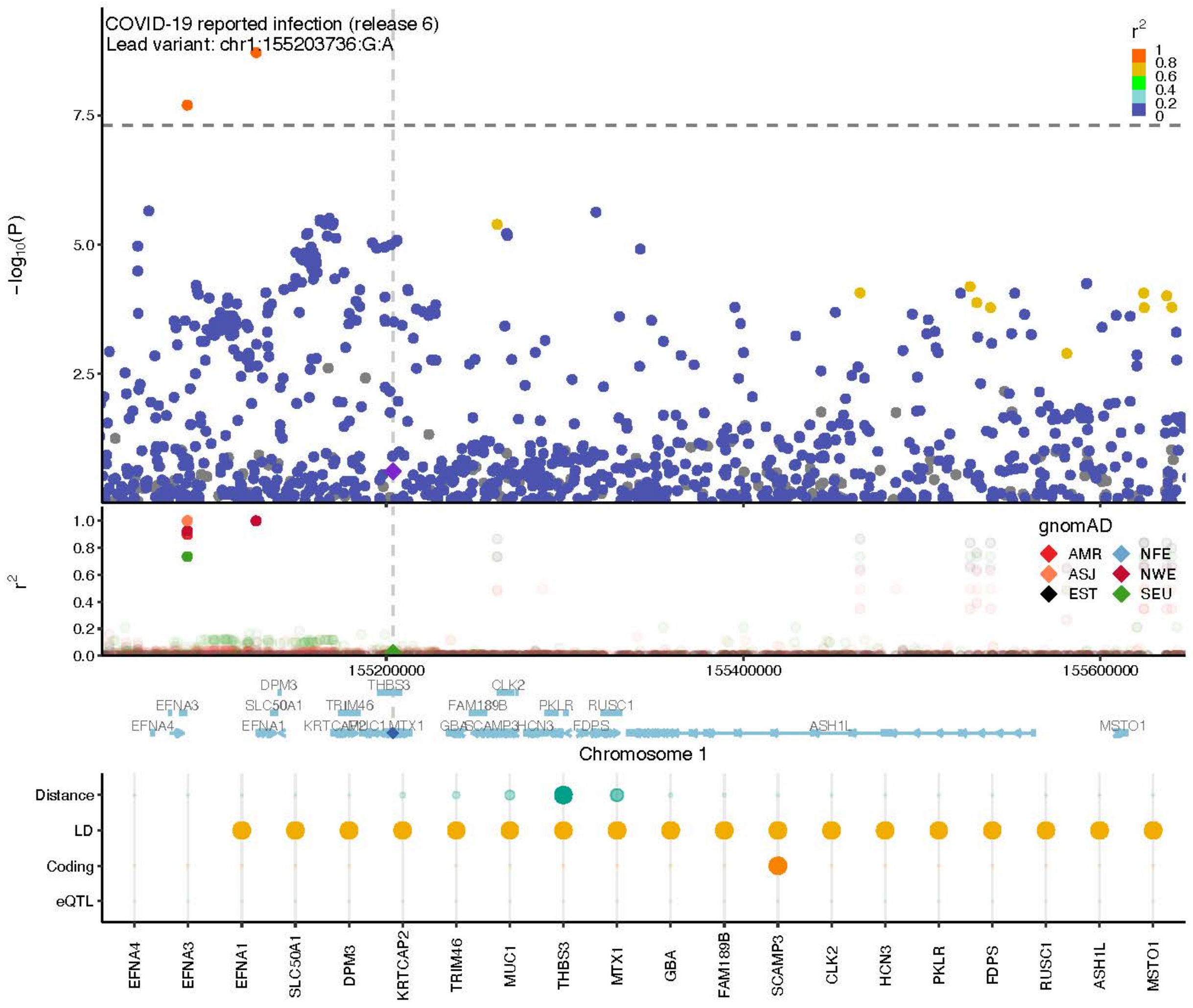
COVID-19 reported infection (release 6)
Lead variant: chr1:155203736:G:A
-log10(P)
r2
gnomAD
AMR
ASJ
EST
NFE
NWE
SEU
155200000
155400000
155600000
Chromosome 1
Distance
LD
Coding
eQTL
EFNA4
EFNA3
EFNA1
SLC50A1
DPM3
KRTCAP2
TRIM46
MUC1
THBS3
MTX1
GBA
FAM189B
SCAMP3
CLK2
HCN3
PKLR
FDPS
RUSC1
ASH1L
MSTO1

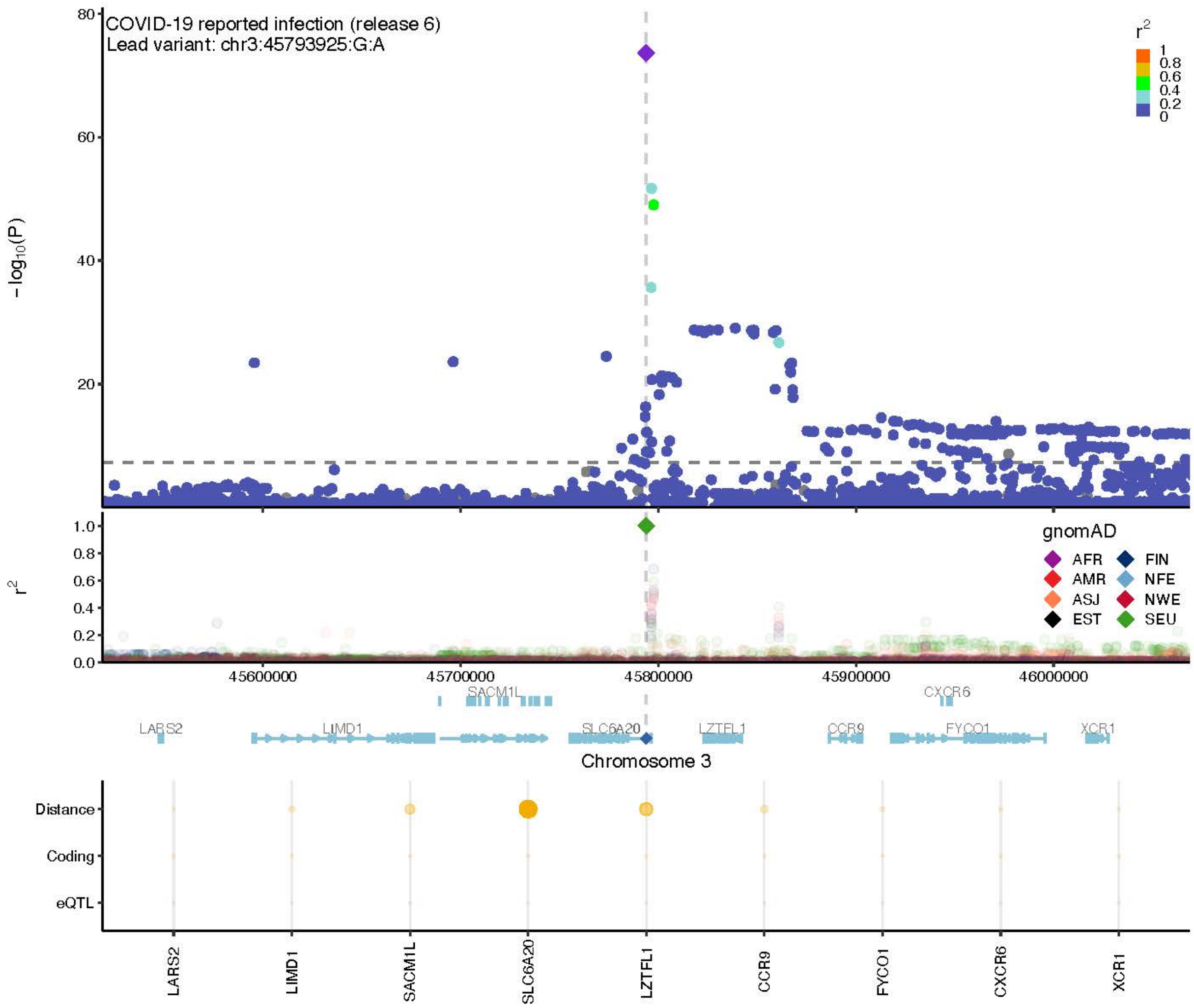
COVID-19 reported infection (release 6)
Lead variant: chr3:45793925:G:A
-log10(P)
r2
gnomAD
AFR
AMR
ASJ
EST
FIN
NFE
NWE
SEU
45600000
45700000
45800000
45900000
46000000
Chromosome 3
LARS2
LIMD1
SACM1L
SLC6A20
LZTFL1
CCR9
FYCO1
CXCR6
XCR1
Distance
Coding
eQTL

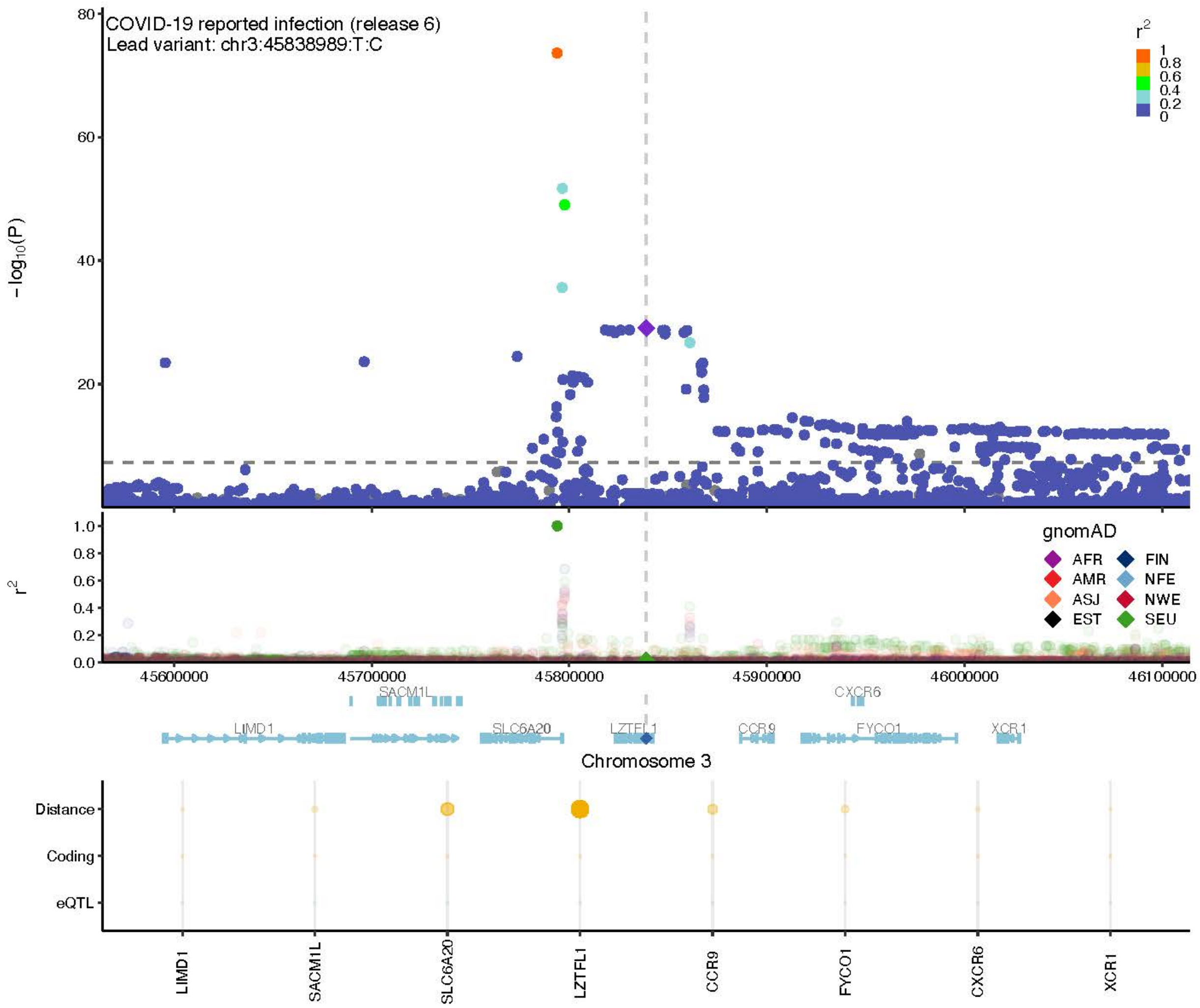
COVID-19 reported infection (release 6)
Lead variant: chr3:45838989:T:C
$-\log_{10}(P)$
r^2
gnomAD
AFR
AMR
ASJ
EST
FIN
NFE
NWE
SEU
SACM1L
CXCR6
LIMD1
SLC6A20
LZTFL1
CCR9
FYCO1
XCR1
Chromosome 3
Distance
Coding
eQTL
LIMD1
SACM1L
SLC6A20
LZTFL1
CCR9
FYCO1
CXCR6
XCR1

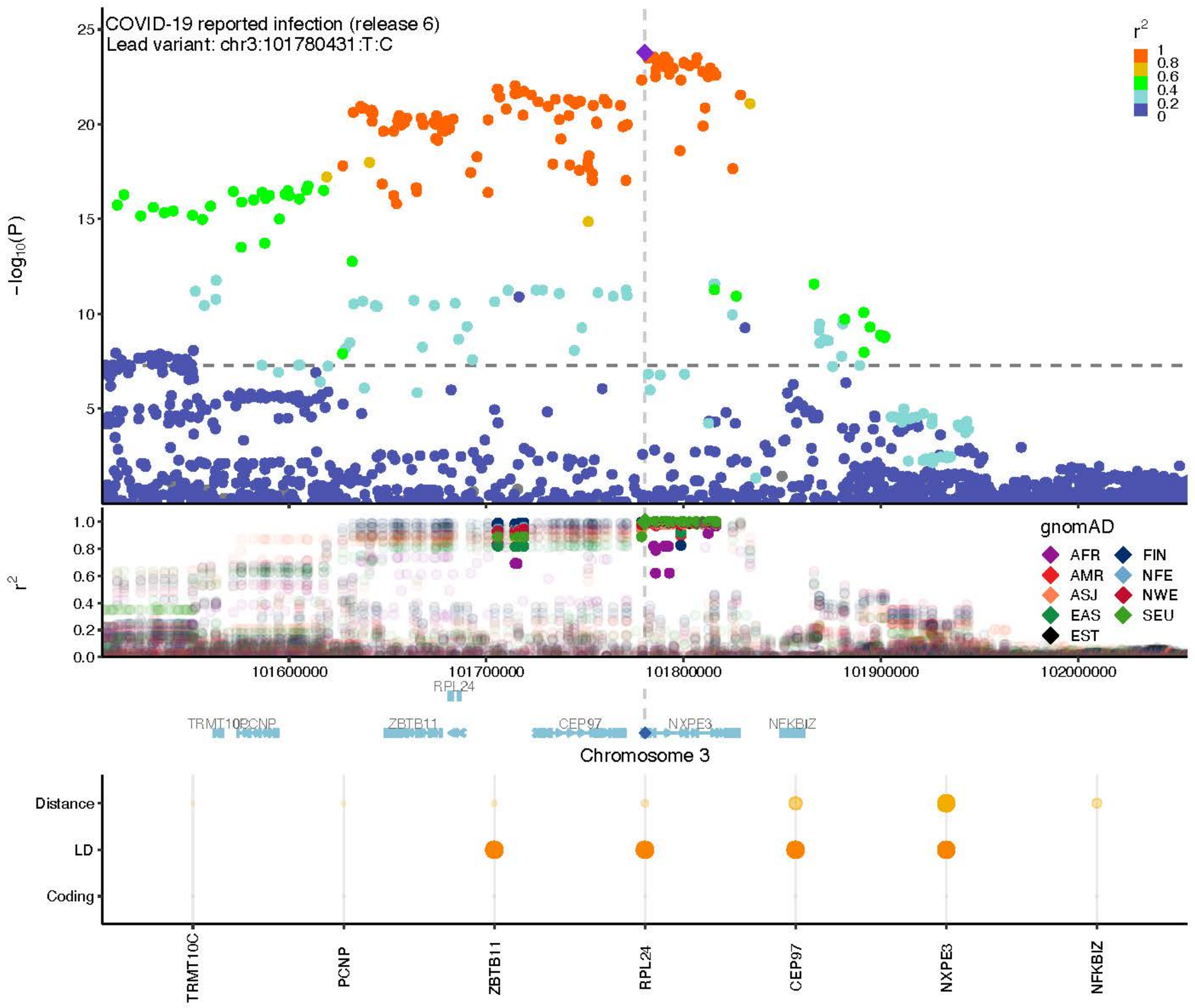
COVID-19 reported infection (release 6)
Lead variant: chr3:101780431:T:C
−log10(P)
r2
gnomAD
AFR
AMR
ASJ
EAS
EST
FIN
NFE
NWE
SEU
101600000
101700000
101800000
101900000
102000000
Chromosome 3
RPL24
TRMT10C
PCNP
ZBTB11
CEP97
NXPE3
NFKBIZ
Distance
LD
Coding

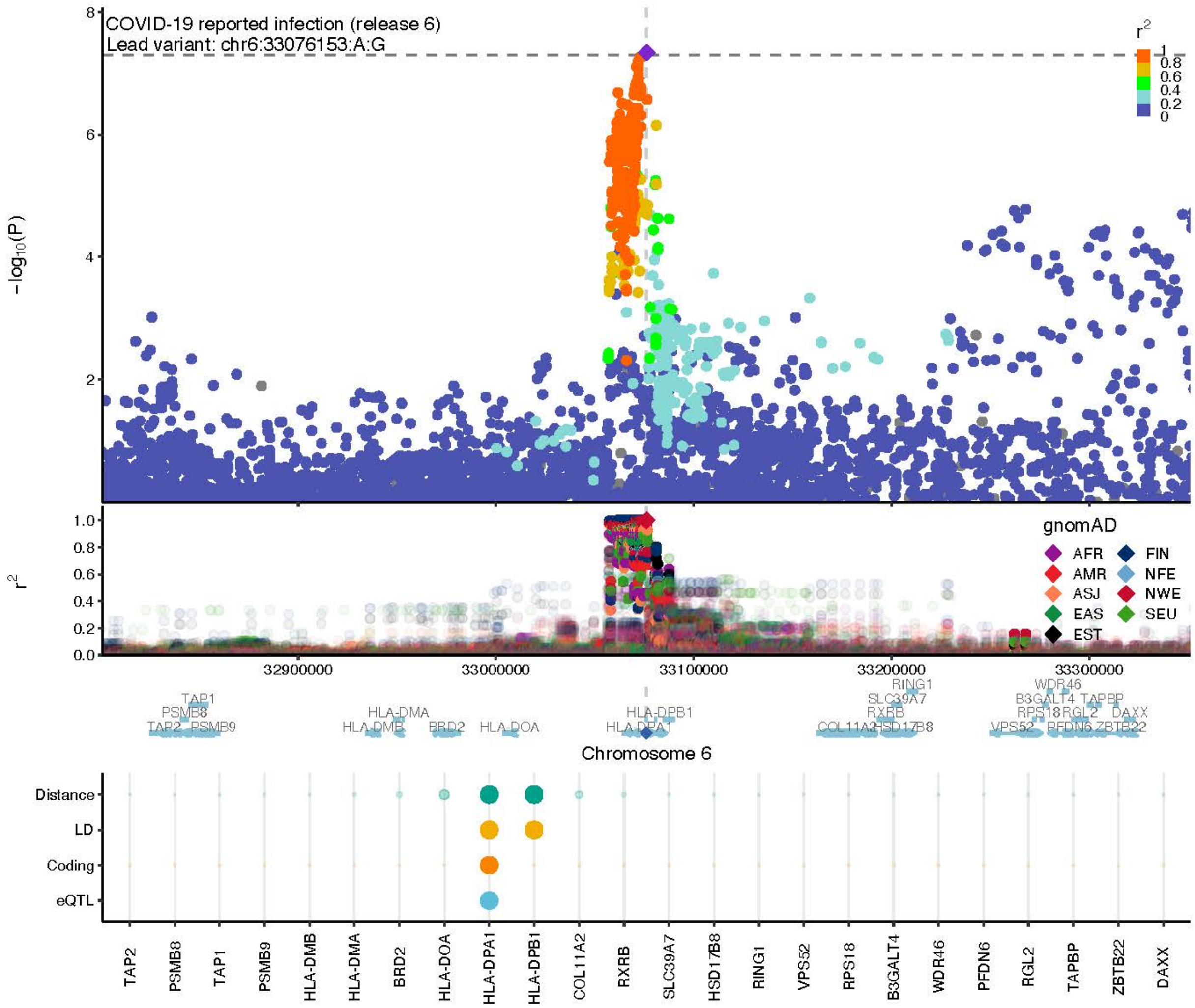
COVID-19 reported infection (release 6)
Lead variant: chr6:33076153:A:G
−log10(P)
r2
gnomAD
AFR
AMR
ASJ
EAS
EST
FIN
NFE
NWE
SEU
32900000
33000000
33100000
33200000
33300000
Chromosome 6
Distance
LD
Coding
eQTL
TAP2
PSMB8
TAP1
PSMB9
HLA-DMB
HLA-DMA
BRD2
HLA-DOA
HLA-DPA1
HLA-DPB1
COL11A2
RXRB
SLC39A7
HSD17B8
RING1
VPS52
RPS18
B3GALT4
WDR46
PFDN6
RGL2
TAPBP
ZBTB22
DAXX

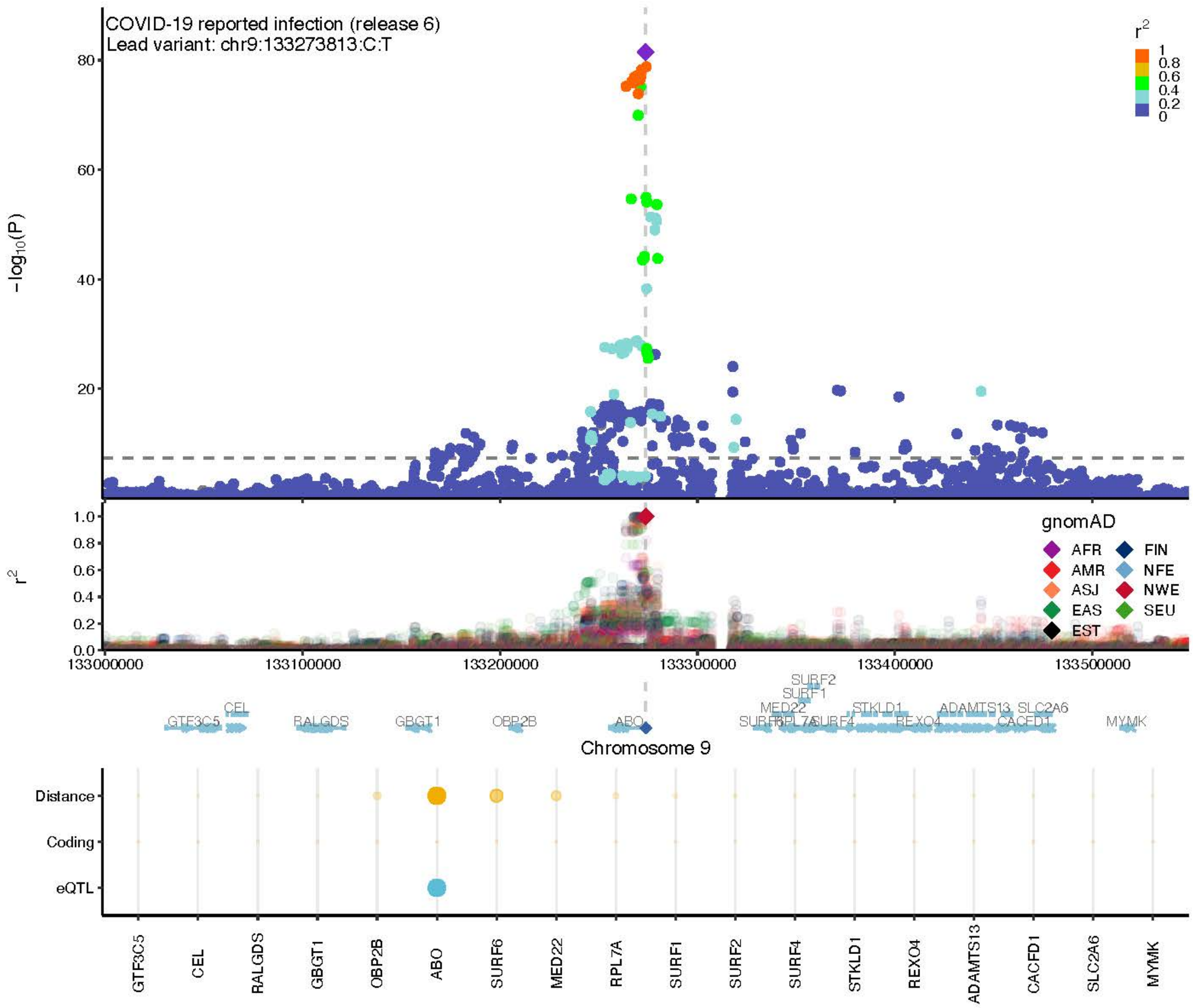
COVID-19 reported infection (release 6)
Lead variant: chr9:133273813:C:T
-log10(P)
r2
gnomAD
AFR
AMR
ASJ
EAS
EST
FIN
NFE
NWE
SEU
Chromosome 9
Distance
Coding
eQTL
GTF3C5
CEL
RALGDS
GBGT1
OBP2B
ABO
SURF6
MED22
RPL7A
SURF1
SURF2
SURF4
STKLD1
REXO4
ADAMTS13
CACFD1
SLC2A6
MYMK

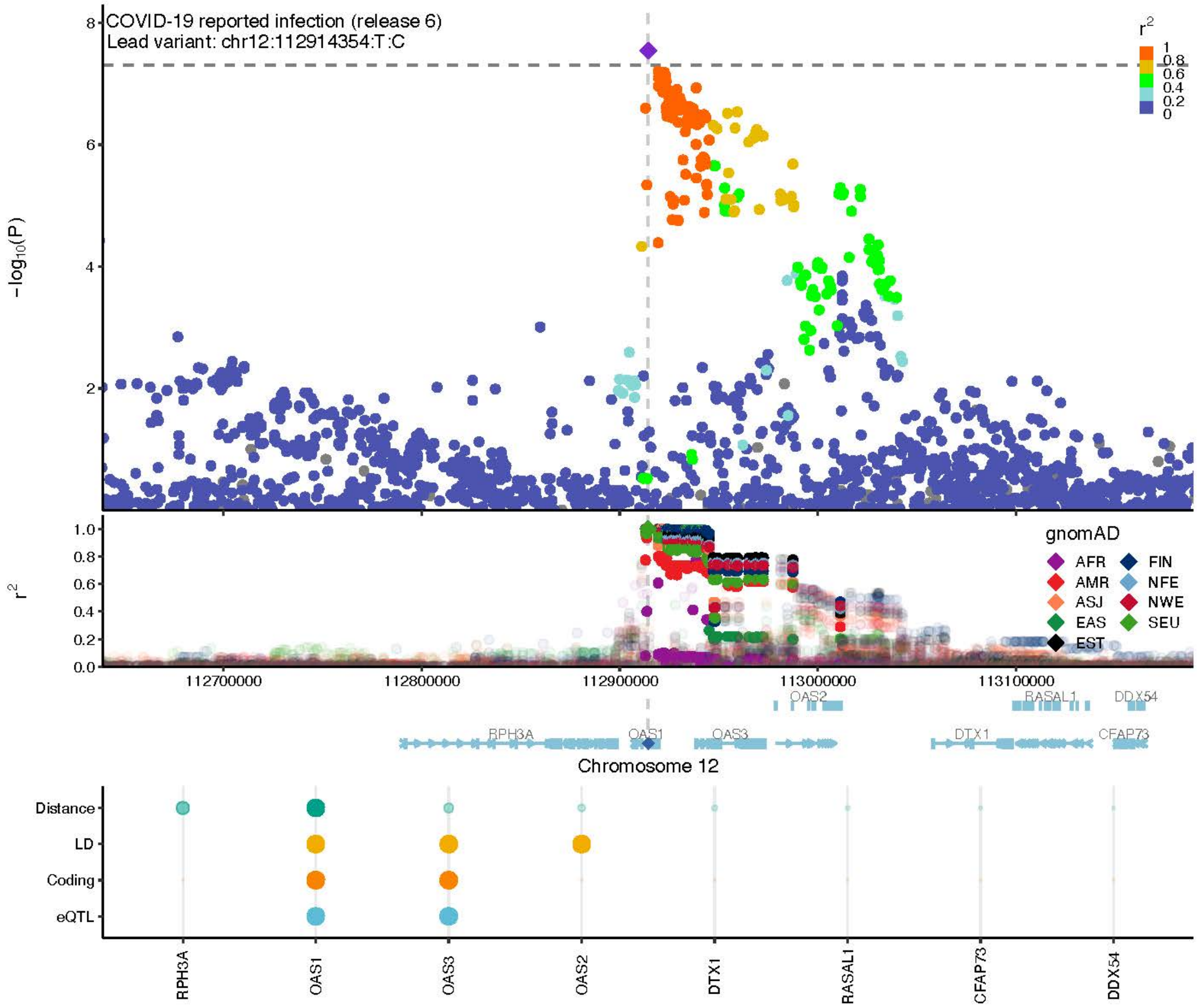
COVID-19 reported infection (release 6)
Lead variant: chr12:112914354:T:C
-log10(P)
r2
1
0.8
0.6
0.4
0.2
0
gnomAD
AFR
AMR
ASJ
EAS
EST
FIN
NFE
NWE
SEU
112700000
112800000
112900000
113000000
113100000
OAS2
RASAL1
DDX54
RPH3A
OAS1
OAS3
DTX1
CFAP73
Chromosome 12
Distance
LD
Coding
eQTL
RPH3A
OAS1
OAS3
OAS2
DTX1
RASAL1
CFAP73
DDX54

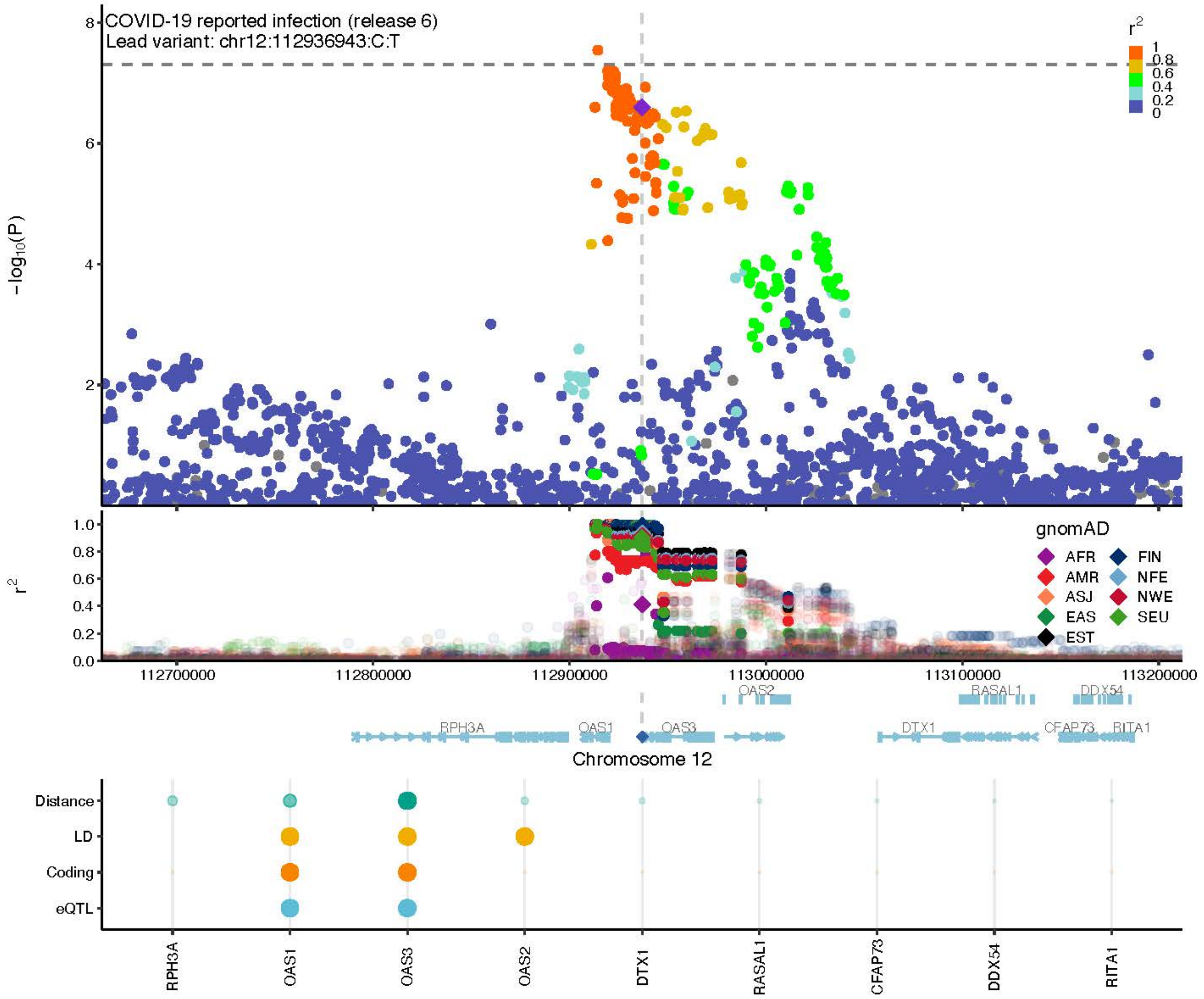
COVID-19 reported infection (release 6)
Lead variant: chr12:112936943:C:T
-log10(P)
r2
gnomAD
AFR
AMR
ASJ
EAS
EST
FIN
NFE
NWE
SEU
112700000
112800000
112900000
113000000
113100000
113200000
Chromosome 12
Distance
LD
Coding
eQTL
RPH3A
OAS1
OAS3
OAS2
DTX1
RASAL1
CFAP73
DDX54
RITA1

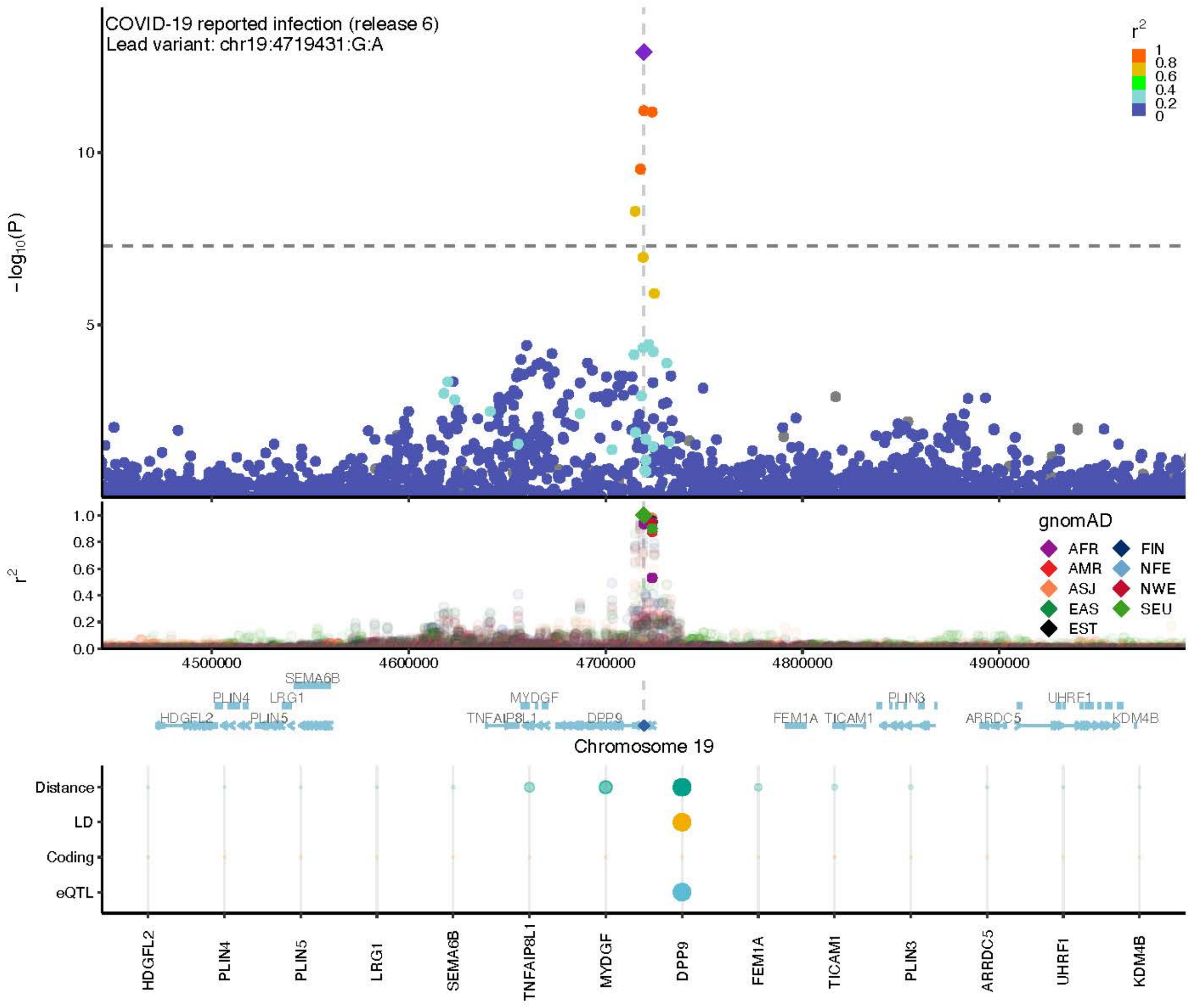
COVID-19 reported infection (release 6)
Lead variant: chr19:4719431:G:A
r2
1
0.8
0.6
0.4
0.2
0
−log10(P)
10
5
r2
1.0
0.8
0.6
0.4
0.2
0.0
gnomAD
AFR
AMR
ASJ
EAS
EST
FIN
NFE
NWE
SEU
4500000
4600000
4700000
4800000
4900000
SEMA6B
PLIN4
LRG1
HDGFL2
PLIN5
MYDGF
TNFAIP8L1
DPP9
FEM1A
TICAM1
PLIN3
UHRF1
ARRDC5
KDM4B
Chromosome 19
Distance
LD
Coding
eQTL
HDGFL2
PLIN4
PLIN5
LRG1
SEMA6B
TNFAIP8L1
MYDGF
DPP9
FEM1A
TICAM1
PLIN3
ARRDC5
UHRF1
KDM4B

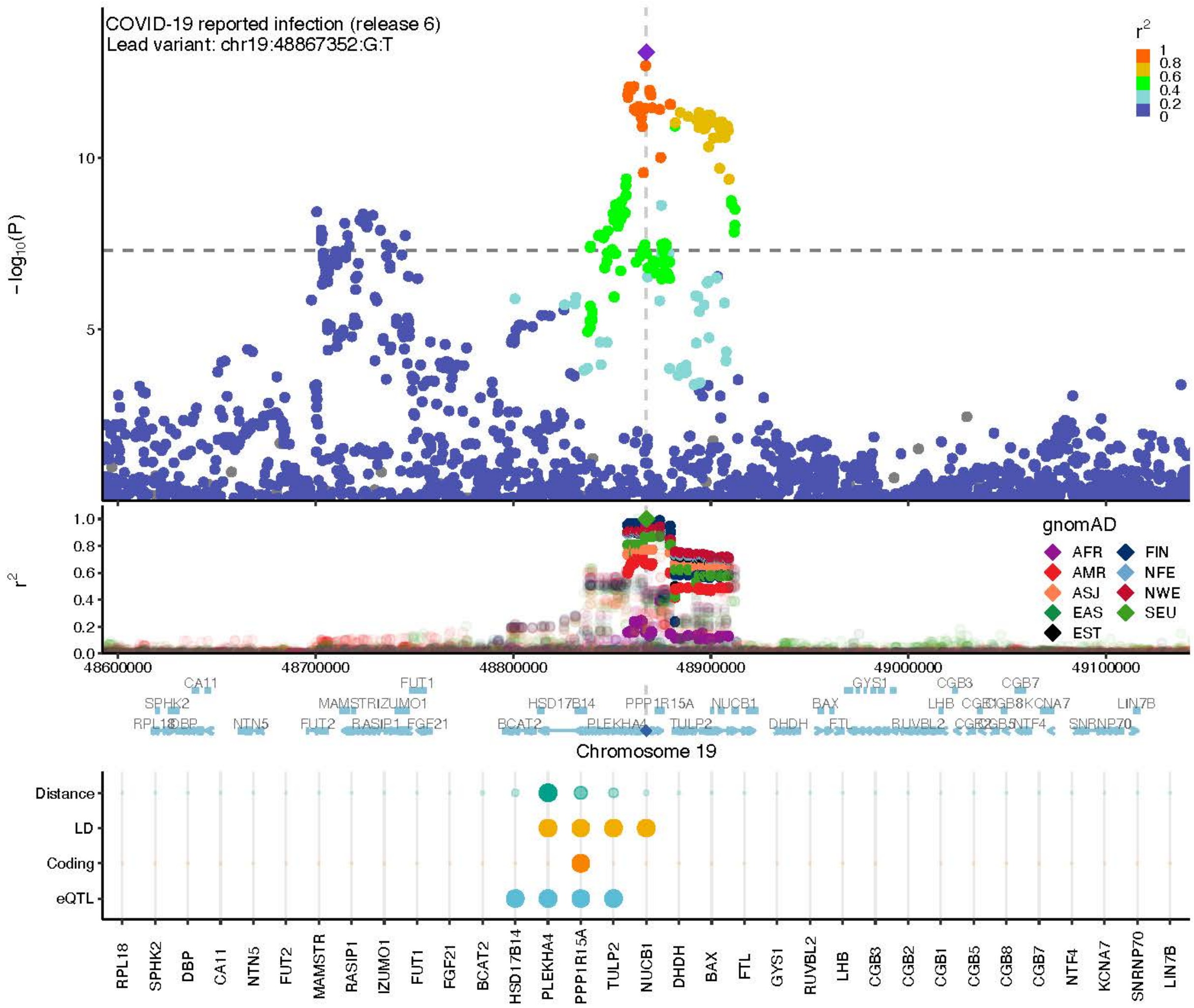
COVID-19 reported infection (release 6)
Lead variant: chr19:48867352:G:T
−log10(P)
r2
gnomAD
AFR
AMR
ASJ
EAS
EST
FIN
NFE
NWE
SEU
Chromosome 19
Distance
LD
Coding
eQTL
RPL18
SPHK2
DBP
CA11
NTN5
FUT2
MAMSTR
RASIP1
IZUMO1
FUT1
FGF21
BCAT2
HSD17B14
PLEKHA4
PPP1R15A
TULP2
NUCB1
DHDH
BAX
FTL
GYS1
RUVBL2
LHB
CGB3
CGB2
CGB1
CGB5
CGB8
CGB7
NTF4
KCNA7
SNRNP70
LIN7B

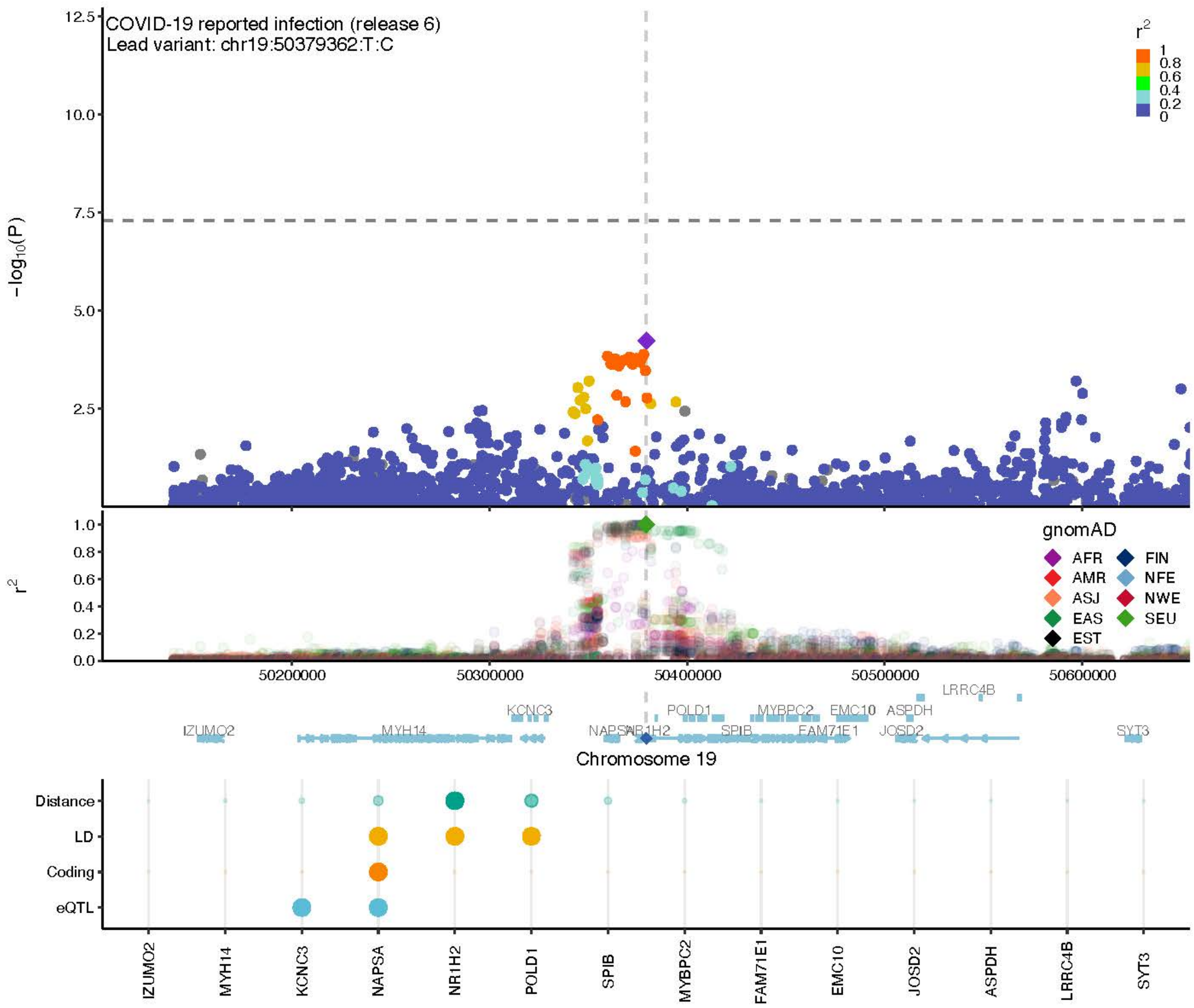
COVID-19 reported infection (release 6)
Lead variant: chr19:50379362:T:C
-log10(P)
r2
gnomAD
AFR
AMR
ASJ
EAS
EST
FIN
NFE
NWE
SEU
Chromosome 19
Distance
LD
Coding
eQTL
IZUMO2
MYH14
KCNC3
NAPSA
NR1H2
POLD1
SPIB
MYBPC2
FAM71E1
EMC10
JOSD2
ASPDH
LRRC4B
SYT3

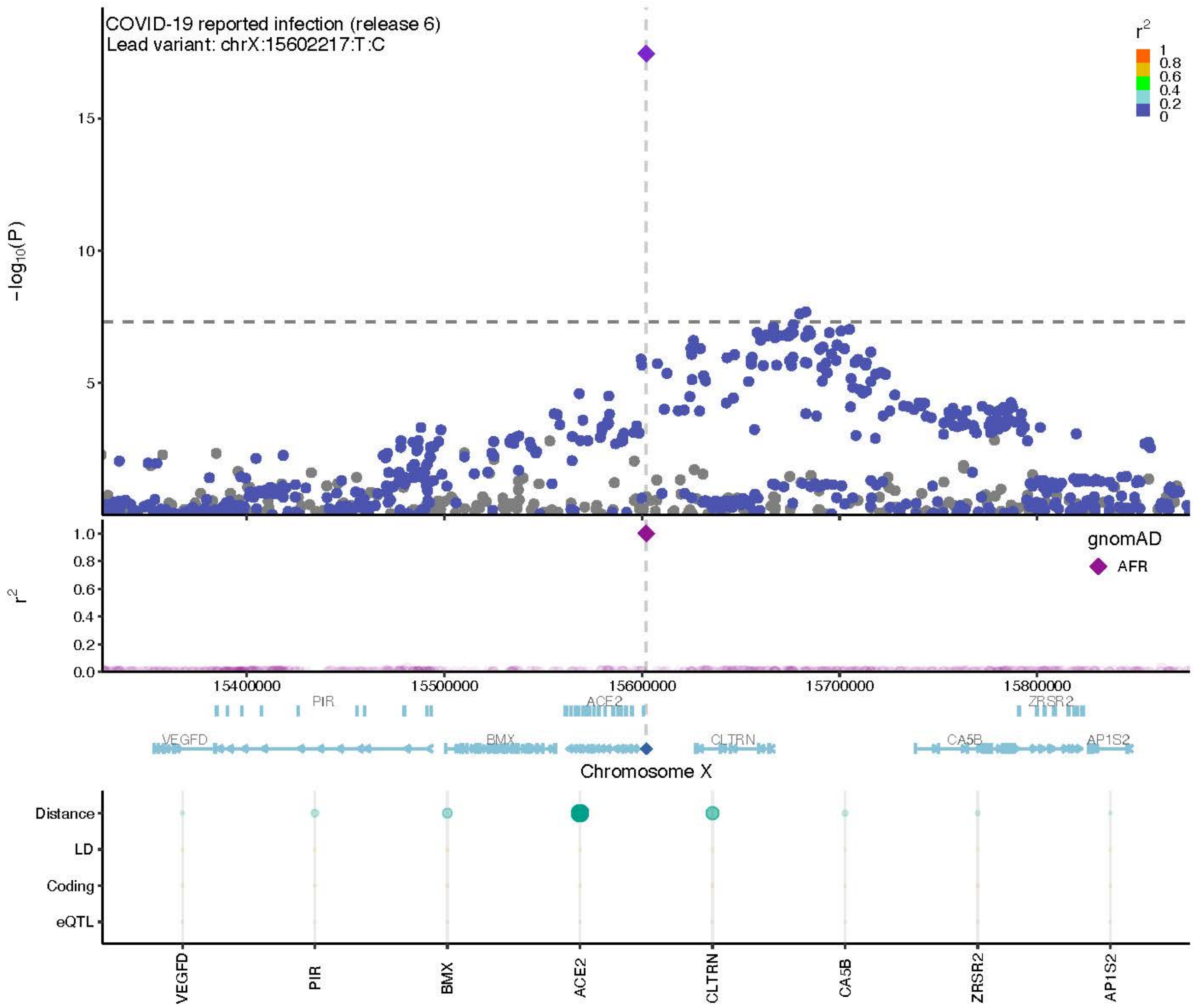
COVID-19 reported infection (release 6)
Lead variant: chrX:15602217:T:C
-log10(P)
r2
gnomAD
AFR
15400000
15500000
15600000
15700000
15800000
PIR
ACE2
ZRSR2
VEGFD
BMX
CLTRN
CA5B
AP1S2
Chromosome X
Distance
LD
Coding
eQTL
VEGFD
PIR
BMX
ACE2
CLTRN
CA5B
ZRSR2
AP1S2

Supplementary Figure 8

Genetic correlations and Mendelian randomization causal estimates between 38 traits and COVID-19 critical illness, hospitalization, and SARS-CoV-2 reported infection. Larger squares correspond to more significant P-values, with genetic correlations or MR causal estimates significantly different from zero at a P < 0.05 shown as a full-sized square. Genetic correlations or causal estimates that are significantly different from zero at a false discovery rate (FDR) of 5% are marked with an asterisk. Two-sided *P*-values were calculated using LDSC for genetic correlations and Inverse variance weighted analysis for MR.

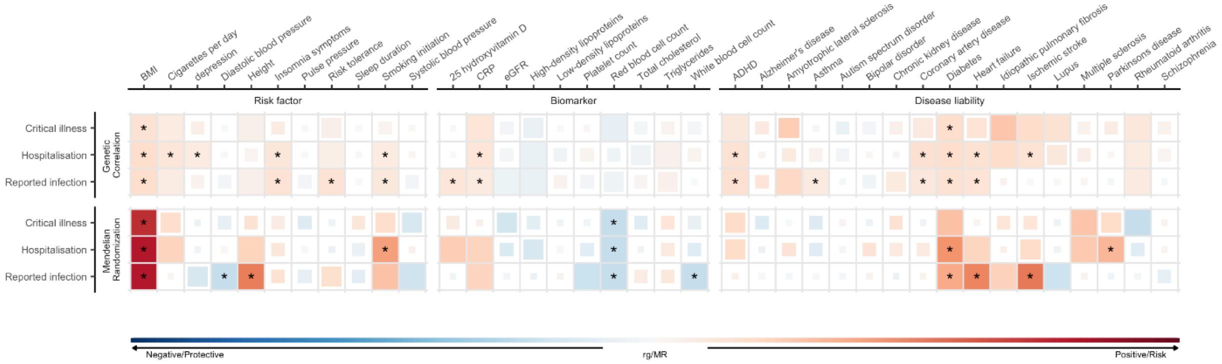

Supplement: Supplementary file 1 — Supplementary Methods with additional references, and Supplementary Figs. 1–8. [file 41586_2022_4826_MOESM1_ESM.pdf]
